# Supplementary material for: Drug repurposing for aging research using model organisms
Source: Aging Cell. 2017 Jun 16;16(5):1006–15. doi: 10.1111/acel.12626 (PMC5595691; doi:10.1111/acel.12626)
Supplement: Supplementary file 7 — Data S1 Zip‐Archive of all report cards. [file ACEL-16-1006-s007.zip › RC_ACT.pdf]

ACT

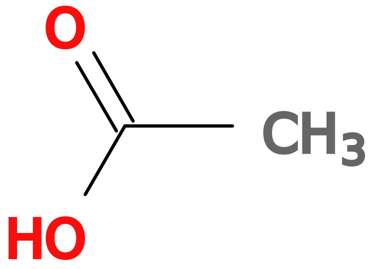

Database identifiers

CHEBI	30089
ZINC	ZINC05224164
eMolecules	883719

Ranking

	Rank	Score
Drosophila	453/697	0.318
C. elegans	496/591	0.072

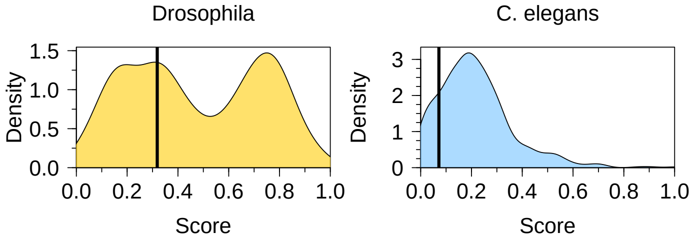

	Ageing implication	Domain conservation	Binding site conservation	Binding affinity	Bioavailability	Lipinski	Promiscuity	Purchasability	Drug approval	**Total**
Drosophila	1.0	0.977	1.0	0.391	(0.9)	0.0	-0.2	0.1	0.075	0.318
C. elegans	1.0	0.982	1.0	0.391	0.252	0.0	-0.2	0.1	0.075	0.072

Names

- **acetate**
- Acetate ion
- acetic acid, ion(1-)
- Ch3-coo(-)
- Ethanoat
- MeCO2 anion

Roles

human metabolite

Status

Number of Rule of 5 violations	0
Binding affinity to original target in log units (RF-Score prediction)	4.56
Burns *C. elegans* bioavailability prediction	-1.99

Compound Target Characteristics

Chloride intracellular channel protein 1

Best gene implication in ageing for this target family came from gene Q9VY78 via mapping the annotation from FlyBase FBgn0030529 annotated in FlyBase 2014-03-11. Annotation GO 8340 (determination of adult lifespan) was Inferred from Mutant Phenotype

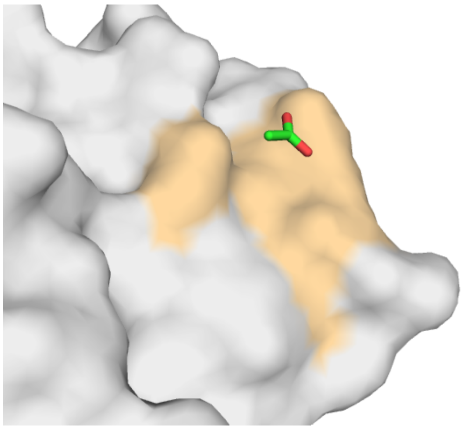

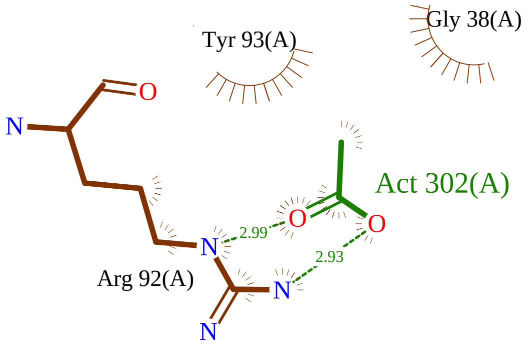

protein	amino acids contacts (binding site)
PDB:4k0g:chainA:O00299	G R Y
tr:A6PVS0:A6PVS0_HUMAN	G R Y
tr:Q5SRT3:Q5SRT3_HUMAN	G R Y
sp:O15247:CLIC2_HUMAN	G R Y
sp:O00299:CLIC1_HUMAN	G R Y
sp:Q96NY7:CLIC6_HUMAN	G R Y
sp:Q6MG61:CLIC1_RAT	G R Y
sp:Q5M883:CLIC2_RAT	G R Y
tr:F1M9X4:F1M9X4_RAT	G R Y
sp:Q811Q2:CLIC6_RAT	G R Y
sp:Q9Z1Q5:CLIC1_MOUSE	G R Y
tr:Q542F1:Q542F1_MOUSE	G R Y
sp:Q8BHB9:CLIC6_MOUSE	G R Y
tr:Q9VY78:Q9VY78_DROME	T G G
sp:Q8WQA4:EXC4_CAEEL	V - -

protein	whole protein ident	simil	domain-based ident	simil	contact-based ident	simil
PDB:4k0g:chainA:O00299	1.0	1.0	0.99	1.0	1.0	1.0
tr:A6PVS0:A6PVS0_HUMAN	0.32	0.45	0.43	0.58	1.0	1.0
tr:Q5SRT3:Q5SRT3_HUMAN	1.0	1.0	1.0	1.0	1.0	1.0
sp:O15247:CLIC2_HUMAN	0.59	0.84	0.6	0.85	1.0	1.0
sp:O00299:CLIC1_HUMAN	1.0	1.0	1.0	1.0	1.0	1.0
sp:Q96NY7:CLIC6_HUMAN	0.22	0.3	0.52	0.72	1.0	1.0
sp:Q6MG61:CLIC1_RAT	0.99	1.0	0.99	1.0	1.0	1.0
sp:Q5M883:CLIC2_RAT	0.59	0.83	0.6	0.85	1.0	1.0
tr:F1M9X4:F1M9X4_RAT	0.25	0.34	0.6	0.82	1.0	1.0
sp:Q811Q2:CLIC6_RAT	0.26	0.35	0.6	0.82	1.0	1.0
sp:Q9Z1Q5:CLIC1_MOUSE	0.98	1.0	0.98	1.0	1.0	1.0
tr:Q542F1:Q542F1_MOUSE	0.98	1.0	0.98	1.0	1.0	1.0
sp:Q8BHB9:CLIC6_MOUSE	0.26	0.35	0.59	0.81	1.0	1.0
tr:Q9VY78:Q9VY78_DROME	0.21	0.57	0.24	0.67	0.0	0.32
sp:Q8WQA4:EXC4_CAEEL	0.2	0.51	0.21	0.58	0.0	0.0

Clic (FBgn0030529) associated phenotypes
gravitaxis defective, heat sensitive, partially lethal - majority die

(Information from FlyBase)

exc-4 (WBGene00001365) associated phenotypes
excretory canal morphology variant, lethal, locomotion variant, pale

(Information from WormBase)

exc-4 (UniProt:Q8WQA4) annotation
Function: May insert into membranes and form chloride ion channels. Involved in the formation of the excretory canal. Required to prevent cystic lumenal expansions in the excretory cell. Not required for formation of the initial tube, but is required for regulating the size of the tube lumen as it grows. (PubMed:17985355).
Subunit: Monomer. (PubMed:17985355).
Subcellular location: Cytoplasm. Membrane ; Single- pass membrane protein Note=Exists both as soluble cytoplasmic protein and as membrane protein with probably a single transmembrane domain. Localizes to various tubular membranes in distinct cell types, including the lumenal membrane of the excretory tubes.
Tissue specificity: Expressed in the secretory system, hypodermis, vulva, pharyngeal muscle, rectal gland, tubular rectal epithelium cells, and tubular neuronal support cells in the head and tail. (PubMed:14684823).
Domain: The N-terminal part (1-55) is necessary and sufficient for translocation from the cytosol to the membrane. (PubMed:14684823).
Domain: Members of this family may change from a globular, soluble state to a state where the N-terminal domain is inserted into the membrane and functions as chloride channel. A conformation change of the N-terminal domain is thought to expose hydrophobic surfaces that trigger membrane insertion (By similarity).

(Information from UniProt)

Histone deacetylase 3

Best gene implication in ageing for this target family came from gene Q94517 annotated in UniProt release 2014_02. Annotation GO 8340 (determination of adult lifespan) was Inferred from Mutant Phenotype

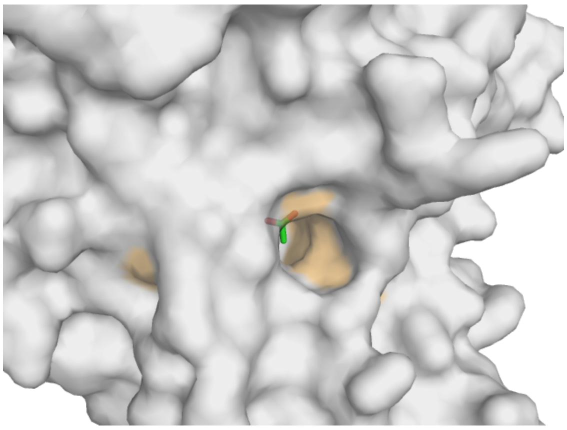

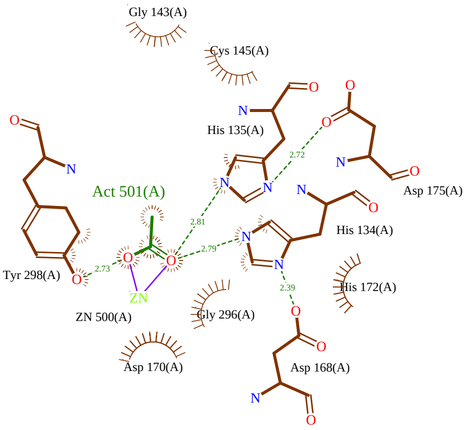

protein	amino acids contacts (binding site)
PDB:4a69:chainA:O15379	H H G C D H D G Y
sp:O15379:HDAC3_HUMAN	H H G C D H D G Y
tr:B3KRS5:B3KRS5_HUMAN	H H G C D H D G Y
sp:Q13547:HDAC1_HUMAN	H H G C D H D G Y
tr:Q6IT96:Q6IT96_HUMAN	H H G C D H D G Y
sp:Q92769:HDAC2_HUMAN	H H G C D H D G Y
tr:J3KPW7:J3KPW7_HUMAN	H H G C D H D G Y
tr:D4AEB0:D4AEB0_RAT	H H G C D H D G Y
sp:O88895:HDAC3_MOUSE	H H G C D H D G Y
tr:Q3UM33:Q3UM33_MOUSE	H H G C D H D G Y
tr:Q7KTS4:Q7KTS4_DROME	H H G C D H D G Y
tr:Q9VNC2:Q9VNC2_DROME	H H G C D H D G Y
sp:Q94517:HDAC1_DROME	H H G C D H D G Y
tr:I1V501:I1V501_DROME	H H G C D H D G Y
sp:P32561:RPD3_YEAST	H H G C D H D G Y

	whole protein		domain-based		contact-based	
protein	ident	simil	ident	simil	ident	simil
PDB:4a69:chainA:O15379	1.0	1.0	1.0	1.0	1.0	1.0
sp:O15379:HDAC3_HUMAN	1.0	1.0	1.0	1.0	1.0	1.0
tr:B3KRS5:B3KRS5_HUMAN	0.48	0.72	0.59	0.84	1.0	1.0
sp:Q13547:HDAC1_HUMAN	0.51	0.77	0.63	0.89	1.0	1.0
tr:Q6IT96:Q6IT96_HUMAN	0.51	0.77	0.63	0.89	1.0	1.0
sp:Q92769:HDAC2_HUMAN	0.5	0.75	0.63	0.89	1.0	1.0
tr:J3KPW7:J3KPW7_HUMAN	0.42	0.63	0.63	0.89	1.0	1.0
tr:D4AEB0:D4AEB0_RAT	0.91	0.96	0.9	0.96	1.0	1.0
sp:O88895:HDAC3_MOUSE	0.99	0.99	1.0	1.0	1.0	1.0
tr:Q3UM33:Q3UM33_MOUSE	1.0	1.0	1.0	1.0	1.0	1.0
tr:Q7KTS4:Q7KTS4_DROME	0.65	0.88	0.7	0.91	1.0	1.0
tr:Q9VNC2:Q9VNC2_DROME	0.65	0.88	0.7	0.91	1.0	1.0
sp:Q94517:HDAC1_DROME	0.46	0.7	0.61	0.88	1.0	1.0
tr:I1V501:I1V501_DROME	0.46	0.7	0.61	0.88	1.0	1.0
sp:P32561:RPD3_YEAST	0.52	0.79	0.6	0.88	1.0	1.0

Hdac3 (FBgn0025825) associated phenotypes

decreased cell growth, dominant, increased cell death, large body, lethal - all die before end of pupal stage, lethal - all die during larval stage, neuroanatomy defective, partially lethal, small body, somatic clone, some die during pupal stage

(Information from FlyBase)

Hdac3 (UniProt:Q7KTS4) annotation

Subcellular location: Nucleus ECO:0000256—PIRNR:PIRNR037913.

(Information from UniProt)

Hdac3 (UniProt:Q9VNC2) annotation
Subcellular location: Nucleus ECO:0000256—PIRNR:PIRNR037913.

(Information from UniProt)

Rpd3 (FBgn0015805) associated phenotypes
decreased cell number, dominant, drug conditional, enhancer of variegation, germline clone, increased cell death, lethal - all die before end of pupal stage, lethal - all die before end of second instar larval stage, long lived, maternal effect, mitotic cell cycle defective, neuroanatomy defective, pair rule phenotype, partially lethal - majority die, polyphasic, poor, somatic clone, some die during pupal stage, some die during second instar larval stage, suppressor of variegation

(Information from FlyBase)

Rpd3 (UniProt:Q94517) annotation
Function: Catalyzes the deacetylation of lysine residues on the N- terminal part of the core histones (H2A, H2B, H3 and H4). Histone deacetylation may constitute a tag for epigenetic repression and plays an important role in transcriptional regulation, cell cycle progression and developmental events. For instance, deacetylation of histone H3 may be a prerequisite for the subsequent recruitment of the histone methyltransferase Su(var)3-9 to histones. Involved in position-effect variegation (PEV).
Subunit: Interacts with Su(var)3-9. Component of a form of the Esc/E(z) complex present specifically during early embryogenesis which is composed of Caf1, esc, E(z), Su(z)12, Pcl and Rpd3. The Esc/E(z) complex may also associate with Pcl and Rpd3 during early embryogenesis. This complex is distinct from the PRC1 complex, which contains many other PcG proteins like Pc, Ph, Psc, Su(z)2. The 2 complexes however cooperate and interact together during the first 3 hours of development to establish PcG silencing. Interacts with the histone methyltransferase Su(var)3-9. Component of a complex that contains at least Rpd3, CoRest and Su(var)3-3/Hdm. Component of the DREAM complex at least composed of Myb, Caf1, mip40, mip120, mip130, E2f2, Dp, Rbf, Rbf2, lin-52, Rpd3 and l(3)mbt. Interacts with neuronal repressor Ttk88. (PubMed:11571273, PubMed:12408863, PubMed:12533794, PubMed:12697833, PubMed:15306652, PubMed:15545624).
Subcellular location: Nucleus ECO:0000305.

(Information from UniProt)

Rpd3 (UniProt:I1V501) annotation
Subcellular location: Nucleus ECO:0000256—PIRNR:PIRNR037913.

(Information from UniProt)

Superoxide dismutase [Cu-Zn]

Best gene implication in ageing for this target family came from gene P61851 annotated in UniProt release 2014_02. Annotation GO 8340 (determination of adult lifespan) was Inferred from Mutant Phenotype

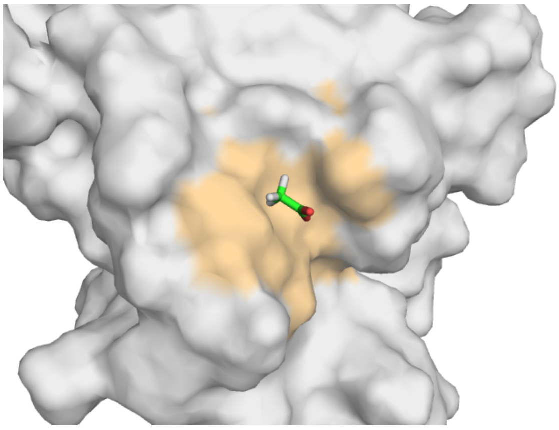

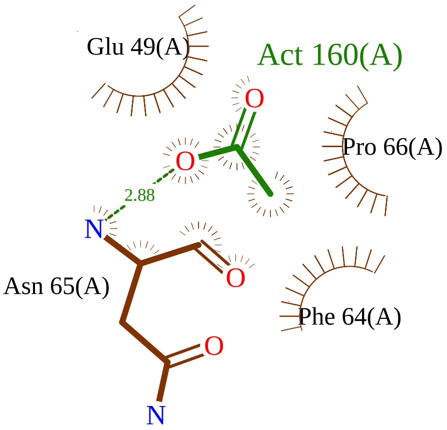

protein	amino acids contacts (binding site)																			
PDB:2c9s:chainA:P00441	E	N	G	P	K	L	T	E	E	H	F	N	P	L	R	K	S	R	N	E
PDB:2wyt:chainA:P00441	E	N	G	P	K	V	T	E	E	H	F	N	P	L	R	K	S	R	N	E
PDB:2xjl:chainA:P00441	E	N	G	P	K	L	T	E	E	H	F	N	P	L	R	K	S	R	N	E
PDB:4a7g:chainA:P00441	E	N	G	P	K	L	T	E	E	H	F	N	P	L	R	K	S	R	N	E
PDB:4a7s:chainA:P00441	E	N	G	P	K	L	T	E	E	H	F	N	P	L	R	K	S	R	N	E
PDB:4a7t:chainA:P00441	E	N	G	P	K	L	T	E	E	H	F	N	P	L	R	K	S	R	N	E
PDB:4a7u:chainA:P00441	E	N	G	P	K	L	T	E	E	H	F	N	P	L	R	K	S	R	N	E
sp:P00441:SODC_HUMAN	E	N	G	P	K	L	T	E	E	H	F	N	P	L	R	K	S	R	N	E
sp:P07632:SODC_RAT	E	G	E	P	V	L	T	E	Q	H	F	N	P	H	K	K	R	R	N	E
tr:Q6LDS4:Q6LDS4_RAT	E	G	E	P	V	L	T	E	Q	H	F	N	P	H	K	K	R	R	N	E
sp:P08228:SODC_MOUSE	E	G	E	P	V	L	T	E	Q	H	F	N	P	H	K	K	R	R	N	E
sp:P61851:SODC_DROME	E	G	T	P	K	L	A	K	E	H	F	N	P	Y	K	G	S	R	H	E
tr:G5ECR5:G5ECR5_CAEEL	D	G	S	F	K	L	A	A	E	H	Y	N	P	H	L	A	S	R	S	D
sp:P34461:SODE_CAEEL	D	G	S	F	K	L	A	A	E	H	Y	N	P	H	L	A	S	R	S	D
sp:P00445:SODC_YEAST	E	S	E	P	T	N	S	N	E	H	F	N	P	F	K	E	S	R	T	E

	whole protein		domain-based		contact-based	
protein	ident	simil	ident	simil	ident	simil
PDB:2c9s:chainA:P00441	0.99	0.99	0.99	0.99	1.0	1.0
PDB:2wyt:chainA:P00441	0.99	1.0	0.99	1.0	0.95	0.99
PDB:2xjl:chainA:P00441	0.95	0.97	0.95	0.97	1.0	1.0
PDB:4a7g:chainA:P00441	0.99	0.99	0.99	0.99	1.0	1.0
PDB:4a7s:chainA:P00441	0.99	1.0	0.99	1.0	1.0	1.0
PDB:4a7t:chainA:P00441	0.99	1.0	0.99	1.0	1.0	1.0
PDB:4a7u:chainA:P00441	0.99	1.0	0.99	1.0	1.0	1.0
sp:P00441:SODC_HUMAN	1.0	1.0	1.0	1.0	1.0	1.0
sp:P07632:SODC_RAT	0.83	0.94	0.83	0.94	0.65	0.75
tr:Q6LDS4:Q6LDS4_RAT	0.82	0.93	0.82	0.93	0.65	0.75
sp:P08228:SODC_MOUSE	0.84	0.94	0.83	0.94	0.65	0.75
sp:P61851:SODC_DROME	0.61	0.84	0.6	0.85	0.6	0.76
tr:G5ECR5:G5ECR5_CAEEL	0.35	0.57	0.44	0.71	0.4	0.62
sp:P34461:SODE_CAEEL	0.35	0.57	0.44	0.71	0.4	0.62
sp:P00445:SODC_YEAST	0.54	0.83	0.55	0.84	0.5	0.7

Sod (FBgn0003462) associated phenotypes

calorie restriction conditional, chemical conditional, chemical sensitive, heat sensitive, increased cell number, locomotor behavior defective, long lived, male limited, oxidative stress response defective, partially, partially lethal - majority die, progressive, radiation sensitive, reduced, short lived

(Information from FlyBase)

Sod (UniProt:P61851) annotation

Function: Destroys radicals which are normally produced within the cells and which are toxic to biological systems.
Cofactor:Cu cationNote=Binds 1 copper ion per subunit.;
Cofactor: Zn(2+)Note=Binds 1 zinc ion per subunit.;

Subunit: Homodimer.
Subcellular location: Cytoplasm.

(Information from UniProt)

sod-4 (UniProt:G5ECR5) annotation
Function: Destroys radicals which are normally produced within the cells and which are toxic to biological systems. (RuleBase:RU000393).
Cofactor: Cu cation Evidence=(RuleBase:RU000393); Note=Binds 1 copper ion per subunit. (RuleBase:RU000393);
Cofactor: Zn(2+) Evidence=(RuleBase:RU000393); Note=Binds 1 zinc ion per subunit. (RuleBase:RU000393);

(Information from UniProt)

sod-4 (UniProt:P34461) annotation
Function: Destroys radicals which are normally produced within the cells and which are toxic to biological systems.
Cofactor:Cu cationNote=Binds 1 copper ion per subunit. ;
Cofactor: Zn(2+)Note=Binds 1 zinc ion per subunit. ;
Subcellular location: Isoform 1: Secreted, extracellular space.
Subcellular location: Isoform 2: Membrane ; Peripheral membrane protein
Tissue specificity: Isoform 2 is preferentially expressed in eggs.

(Information from UniProt)

GTPase HRas

Best gene implication in ageing for this target family came from gene P01112 annotated in UniProt release 2014_02. Annotation GO subterm of 7568 (aging) was Inferred from Direct Assay

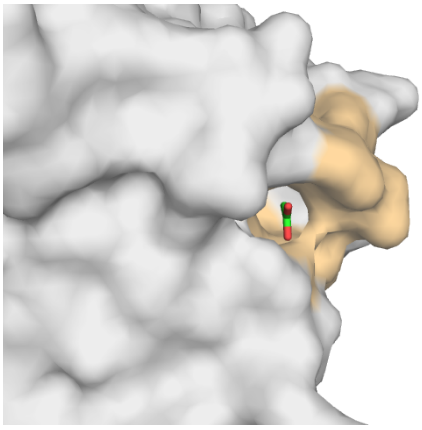

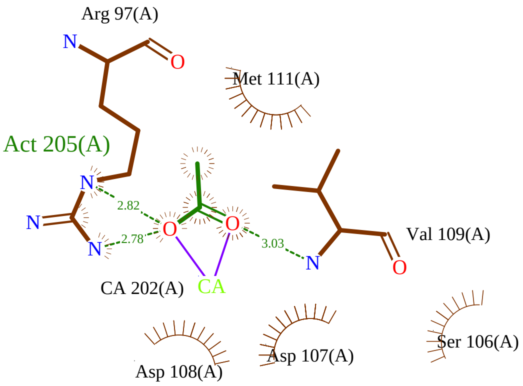

protein	amino acids contacts (binding site)
PDB:3k8y:chainA:P01112	R K S D D V M Y
PDB:3lbh:chainA:P01112	R K S D D V M Y
PDB:3lbi:chainA:P01112	R K S D D V M Y
PDB:3oiu:chainA:P01112	R K S D D V M Y
PDB:3oiw:chainA:P01112	R K S D D V M Y
PDB:4dlt:chainA:P01112	R K S D D V M Y
PDB:4dlu:chainA:P01112	R K S D D V M Y
PDB:4dlw:chainA:P01112	R K S D D V M Y
PDB:4g0n:chainA:P01112	R K S D D V M Y
sp:P01111:RASN_HUMAN	R K S D D V M Y
tr:Q5U091:Q5U091_HUMAN	R K S D D V M Y
sp:P01112:RASH_HUMAN	R K S D D V M Y
sp:P20171:RASH_RAT	R K S D D V M Y
sp:Q04970:RASN_RAT	R K S D D V M Y
sp:Q61411:RASH_MOUSE	R K S D D V M Y
tr:Q9D091:Q9D091_MOUSE	R K S D D V M Y
tr:Q4FJP3:Q4FJP3_MOUSE	R K S D D V M Y
sp:P08646:RAS1_DROME	R K A E E V M Y
sp:P22981:LET60_CAEEL	R R S D D V M Y
sp:P01119:RAS1_YEAST	Y Q S D Y I V L
sp:P01120:RAS2_YEAST	Y L T D Y V I M

	whole protein		domain-based		contact-based	
protein	ident	simil	ident	simil	ident	simil
PDB:3k8y:chainA:P01112	1.0	1.0	1.0	1.0	1.0	1.0
PDB:3lbh:chainA:P01112	1.0	1.0	1.0	1.0	1.0	1.0
PDB:3lbi:chainA:P01112	1.0	1.0	1.0	1.0	1.0	1.0
PDB:3oiu:chainA:P01112	0.99	1.0	0.99	1.0	1.0	1.0
PDB:3oiw:chainA:P01112	0.99	1.0	0.99	1.0	1.0	1.0
PDB:4dlt:chainA:P01112	1.0	1.0	1.0	1.0	1.0	1.0
PDB:4dlu:chainA:P01112	1.0	1.0	1.0	1.0	1.0	1.0
PDB:4dlw:chainA:P01112	1.0	1.0	1.0	1.0	1.0	1.0
PDB:4g0n:chainA:P01112	1.0	1.0	1.0	1.0	1.0	1.0
sp:P01111:RASN_HUMAN	0.85	0.96	0.9	0.98	1.0	1.0
tr:Q5U091:Q5U091_HUMAN	0.85	0.96	0.9	0.98	1.0	1.0
sp:P01112:RASH_HUMAN	1.0	1.0	1.0	1.0	1.0	1.0
sp:P20171:RASH_RAT	1.0	1.0	1.0	1.0	1.0	1.0
sp:Q04970:RASN_RAT	0.85	0.96	0.9	0.98	1.0	1.0
sp:Q61411:RASH_MOUSE	1.0	1.0	1.0	1.0	1.0	1.0
tr:Q9D091:Q9D091_MOUSE	0.86	0.96	0.9	0.98	1.0	1.0
tr:Q4FJP3:Q4FJP3_MOUSE	0.84	0.94	0.9	0.98	1.0	1.0
sp:P08646:RAS1_DROME	0.77	0.91	0.85	0.95	0.63	0.78
sp:P22981:LET60_CAEEL	0.74	0.89	0.81	0.94	0.88	0.97
sp:P01119:RAS1_YEAST	0.34	0.52	0.61	0.87	0.25	0.62
sp:P01120:RAS2_YEAST	0.34	0.5	0.62	0.88	0.25	0.53

Ras85D (FBgn0003205) associated phenotypes

RU486 conditional, cell autonomous, cell death defective, cell non-autonomous, cell polarity defective, cell size defective, conditional, decreased cell death, decreased cell number, decreased cell size, developmental rate defective, dominant, endocytosis defective, female semi-sterile, germline clone, heat sensitive, hyperplasia, increased cell death, increased cell growth, increased cell number, increased cell size, large body, learning defective, lethal - all die before end of P-stage, lethal - all die before end of pupal stage, majority die during P-stage, maternal effect, melanotic mass phenotype, mitotic cell cycle defective, neoplasia, neuroanatomy defective, neurophysiology defective, partially, partially lethal - majority die, short lived, size defective, small body, somatic clone, some die during P-stage, some die during pharate adult stage, some die during pupal stage, temperature conditional, terminal phenotype, tumorigenic

(Information from FlyBase)

Ras85D (UniProt:P08646) annotation

Function: May mediate a signal that determines the fate of photoreceptor cells in the developing compound eye. Ras proteins bind GDP/GTP and possess intrinsic GTPase activity.

Enzyme regulation: Alternates between an inactive form bound to GDP and an active form bound to GTP. Activated by a guanine nucleotide-exchange factor (GEF) and inactivated by a GTPase- activating protein (GAP).

Subcellular location: Cell membrane (PubMed:18503409); Lipid-anchor (PubMed:18503409); Cytoplasmic side (PubMed:18503409). Note=Loss of prenylation causes protein location to the cytoplasm.

Tissue specificity: Expressed in the posterior termini of the embryo, restricted mainly to the embryonic central nervous system, and in the eye imaginal disk. (PubMed:7873789).

(Information from UniProt)

let-60 (WBGene00002335) associated phenotypes

AWA odorant chemotaxis defective, AWC odorant chemotaxis defective, L2 arrest, L3 arrest, antibody staining reduced, antibody staining variant, axon outgrowth variant, bloated, cell differentiation variant, egg laying defective, egg laying imipramine resistant, egg laying variant, germ cell development variant, gonad arm morphology variant, lethal, locomotion variant, male mating defective, mid larval lethal, multivulva, neuron degeneration, oocyte accumulation, oocytes small, organism starvation response variant, protein expression absent, reduced brood size, rod like larval lethal, sterile, unfertilized oocytes laid, vulval cell induction increased, vulvaless

(Information from WormBase)

let-60 (UniProt:P22981) annotation

Function: The level of let-60 controls the switch between vulval and hypodermal cell fates during C.elegans vulval induction. May stimulate the guanine nucleotide exchange factor (GEF) activity of rap-1.

Subunit: Interacts with soc-2. (PubMed:9674433).

Subcellular location: Cell membrane; Lipid-anchor.

(Information from UniProt)

GTPase KRas

Best gene implication in ageing for this target family came from gene P01112 annotated in UniProt release 2014_02. Annotation GO subterm of 7568 (aging) was Inferred from Direct Assay

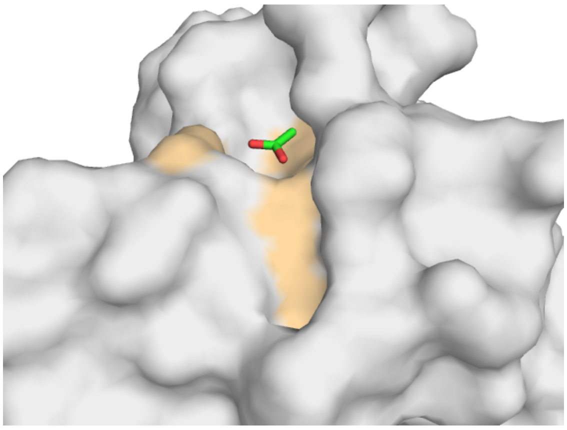

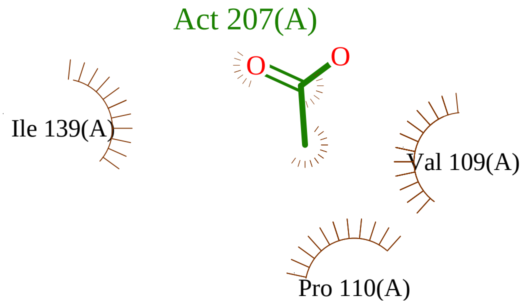

protein	amino acids contacts (binding site)							
PDB:1d8d:chainP:P01116	-	-	-	-	-	K	K	C
PDB:4dst:chainA:P01116	R	V	P	G	I	K	-	-
tr:L7RSL8:L7RSL8_HUMAN	R	V	P	G	I	V	K	C
sp:P01112:RASH_HUMAN	R	V	P	G	I	M	K	C
sp:P01116:RASK_HUMAN	R	V	P	G	I	V	K	C
tr:I1SRC5:I1SRC5_HUMAN	R	V	P	G	I	V	K	C
sp:P20171:RASH_RAT	R	V	P	G	I	M	K	C
sp:P08644:RASK_RAT	R	V	P	G	I	V	K	C
tr:A0JN17:A0JN17_RAT	R	V	P	G	I	K	R	C
sp:Q61411:RASH_MOUSE	R	V	P	G	I	M	K	C
sp:P32883:RASK_MOUSE	R	V	P	G	I	V	K	C
tr:Q0VDV7:Q0VDV7_MOUSE	R	V	P	G	I	V	K	C
tr:Q5J7N1:Q5J7N1_MOUSE	R	V	P	G	I	K	R	C
sp:P08646:RAS1_DROME	R	V	P	G	I	-	K	C
sp:P22981:LET60_CAEEL	R	V	P	G	I	-	K	C
sp:P01119:RAS1_YEAST	Y	I	P	N	A	S	C	C
sp:P01120:RAS2_YEAST	Y	V	P	N	A	G	C	C

	whole protein		domain-based		contact-based	
protein	ident	simil	ident	simil	ident	simil
PDB:1d8d:chainP:P01116	0.36	0.68	0.36	0.68	0.38	0.0
PDB:4dst:chainA:P01116	0.91	0.96	0.93	0.97	0.75	0.5
tr:L7RSL8:L7RSL8_HUMAN	1.0	1.0	1.0	1.0	0.88	0.89
sp:P01112:RASH_HUMAN	0.86	0.95	0.87	0.96	0.88	0.89
sp:P01116:RASK_HUMAN	1.0	1.0	1.0	1.0	0.88	0.89
tr:I1SRC5:I1SRC5_HUMAN	0.64	0.64	1.0	1.0	0.88	0.89
sp:P20171:RASH_RAT	0.86	0.95	0.87	0.96	0.88	0.89
sp:P08644:RASK_RAT	0.99	1.0	0.99	1.0	0.88	0.89
tr:A0JN17:A0JN17_RAT	0.89	0.95	0.9	0.96	0.88	0.97
sp:Q61411:RASH_MOUSE	0.86	0.95	0.87	0.96	0.88	0.89
sp:P32883:RASK_MOUSE	0.99	1.0	0.99	1.0	0.88	0.89
tr:Q0VDV7:Q0VDV7_MOUSE	0.99	1.0	0.99	1.0	0.88	0.89
tr:Q5J7N1:Q5J7N1_MOUSE	0.88	0.95	0.89	0.95	0.88	0.97
sp:P08646:RAS1_DROME	0.74	0.88	0.75	0.88	0.88	0.75
sp:P22981:LET60_CAEEL	0.75	0.89	0.75	0.89	0.88	0.75
sp:P01119:RAS1_YEAST	0.34	0.52	0.45	0.66	0.25	0.42
sp:P01120:RAS2_YEAST	0.34	0.5	0.46	0.67	0.38	0.42

Ras85D (FBgn0003205) associated phenotypes

RU486 conditional, cell autonomous, cell death defective, cell non-autonomous, cell polarity defective, cell size defective, conditional, decreased cell death, decreased cell number, decreased cell size, developmental rate defective, dominant, endocytosis defective, female semi-sterile, germline clone, heat sensitive, hyperplasia, increased cell death, increased cell growth, increased cell number, increased cell size, large body, learning defective, lethal - all die before end of P-stage, lethal - all die before end of pupal stage, majority die during P-stage, maternal effect, melanotic mass phenotype, mitotic cell cycle defective, neoplasia, neuroanatomy defective, neurophysiology defective, partially, partially lethal - majority die, short lived, size defective, small body, somatic clone, some die during P-stage, some die during pharate adult stage, some die during pupal stage, temperature conditional, terminal phenotype, tumorigenic

(Information from FlyBase)

Ras85D (UniProt:P08646) annotation

Function: May mediate a signal that determines the fate of photoreceptor cells in the developing compound eye. Ras proteins bind GDP/GTP and possess intrinsic GTPase activity.
Enzyme regulation: Alternates between an inactive form bound to GDP and an active form bound to GTP. Activated by a guanine nucleotide-exchange factor (GEF) and inactivated by a GTPase- activating protein (GAP).
Subcellular location: Cell membrane (PubMed:18503409); Lipid-anchor (PubMed:18503409); Cytoplasmic side (PubMed:18503409). Note=Loss of prenylation causes protein location to the cyto-

plasm.
Tissue specificity: Expressed in the posterior termini of the embryo, restricted mainly to the embryonic central nervous system, and in the eye imaginal disk. (PubMed:7873789).

(Information from UniProt)

let-60 (WBGene00002335) associated phenotypes

AWA odorant chemotaxis defective, AWC odorant chemotaxis defective, L2 arrest, L3 arrest, antibody staining reduced, antibody staining variant, axon outgrowth variant, bloated, cell differentiation variant, egg laying defective, egg laying imipramine resistant, egg laying variant, germ cell development variant, gonad arm morphology variant, lethal, locomotion variant, male mating defective, mid larval lethal, multivulva, neuron degeneration, oocyte accumulation, oocytes small, organism starvation response variant, protein expression absent, reduced brood size, rod like larval lethal, sterile, unfertilized oocytes laid, vulval cell induction increased, vulvaless

(Information from WormBase)

let-60 (UniProt:P22981) annotation

Function: The level of let-60 controls the switch between vulval and hypodermal cell fates during C.elegans vulval induction. May stimulate the guanine nucleotide exchange factor (GEF) activity of rap-1.
Subunit: Interacts with soc-2. (PubMed:9674433).
Subcellular location: Cell membrane; Lipid-anchor.

(Information from UniProt)

High affinity nerve growth factor receptor

Best gene implication in ageing for this target family came from gene P35739 annotated in UniProt release 2014_02. Annotation GO 7568 (aging) was Inferred from Expression Pattern

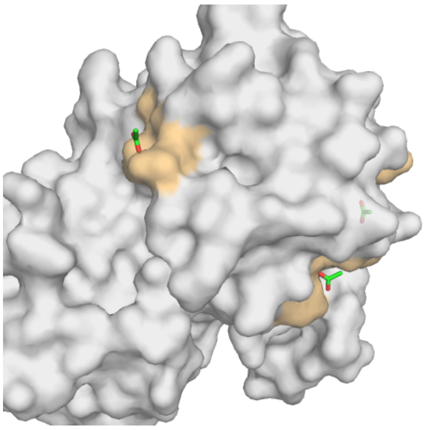

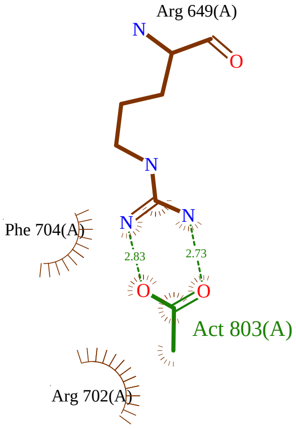

protein	amino acids contacts (binding site)											
PDB:4pmm:chainA:P04629	R	R	Y	R	F	W	T	K	Q	Y	E	P
PDB:4pmp:chainA:P04629	R	R	Y	R	F	W	T	K	Q	Y	E	P
PDB:4pms:chainA:P04629	R	R	Y	R	F	W	T	K	Q	Y	E	P
PDB:4pmt:chainA:P04629	R	R	Y	R	F	W	T	K	Q	Y	E	P
sp:P04629:NTRK1_HUMAN	R	R	Y	R	F	W	T	K	Q	Y	E	P
tr:J3KP20:J3KP20_HUMAN	R	R	Y	R	F	W	T	K	Q	Y	E	P
sp:P35739:NTRK1_RAT	R	R	Y	R	F	W	T	K	Q	Y	E	P
sp:Q3UFB7:NTRK1_MOUSE	R	R	Y	R	F	W	T	K	Q	Y	E	P
tr:G4RZJ9:G4RZJ9_CAEEL	E	R	Y	H	F	W	S	R	Q	D	L	P

protein	whole protein ident	simil	domain-based ident	simil	contact-based ident	simil
PDB:4pmm:chainA:P04629	0.99	0.99	1.0	1.0	1.0	1.0
PDB:4pmp:chainA:P04629	0.99	0.99	1.0	1.0	1.0	1.0
PDB:4pms:chainA:P04629	0.99	0.99	1.0	1.0	1.0	1.0
PDB:4pmt:chainA:P04629	0.99	0.99	1.0	1.0	1.0	1.0
sp:P04629:NTRK1_HUMAN	1.0	1.0	1.0	1.0	1.0	1.0
tr:J3KP20:J3KP20_HUMAN	0.99	0.99	1.0	1.0	1.0	1.0
sp:P35739:NTRK1_RAT	0.86	0.95	0.97	0.99	1.0	1.0
sp:Q3UFB7:NTRK1_MOUSE	0.87	0.95	0.97	0.99	1.0	1.0
tr:G4RZJ9:G4RZJ9_CAEEL	0.16	0.48	0.38	0.75	0.5	0.64

Amyloid beta A4 protein

Best gene implication in ageing for this target family came from gene P05067 via mapping the annotation from Ensembl ENSG00000142192 via mapping the annotation from EntrezGene 351 via mapping the annotation from GenAgeHuman 0137 annotated in GenAge release 17.

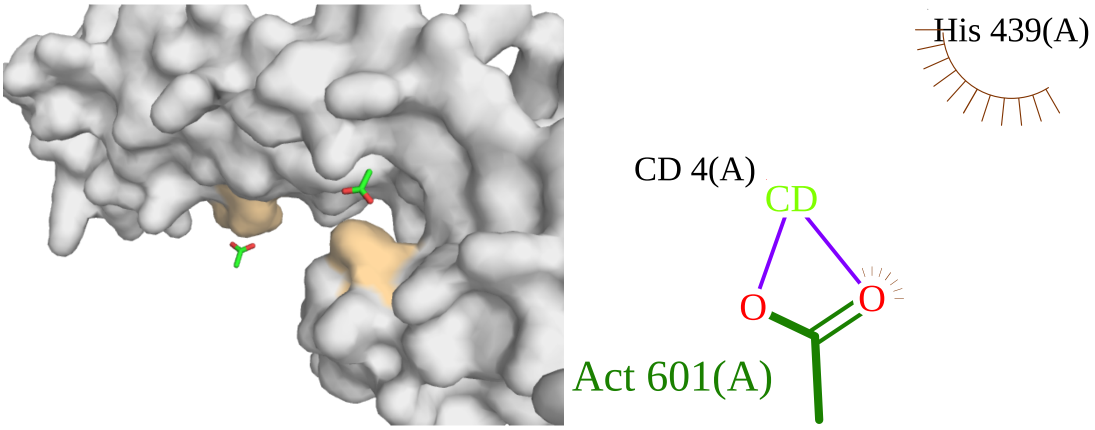

protein	amino acids contacts (binding site)		
PDB:3umh:chainA:P05067	E	E	H
PDB:3umi:chainA:P05067	E	E	H
PDB:3umk:chainA:P05067	E	E	H
tr:H7C104:H7C104_HUMAN	E	E	H
tr:E9PEV0:E9PEV0_HUMAN	E	E	H
tr:E9PG40:E9PG40_HUMAN	E	E	H
sp:P05067:A4_HUMAN	E	E	H
sp:P08592:A4_RAT	E	E	H
tr:Q6GR78:Q6GR78_MOUSE	E	E	H
sp:P12023:A4_MOUSE	E	E	H
sp:P14599:A4_DROME	T	Q	H
sp:Q10651:A4_CAEEL	K	S	H

protein	whole protein ident	simil	domain-based ident	simil	contact-based ident	simil
PDB:3umh:chainA:P05067	1.0	1.0	1.0	1.0	1.0	1.0
PDB:3umi:chainA:P05067	1.0	1.0	1.0	1.0	1.0	1.0
PDB:3umk:chainA:P05067	1.0	1.0	1.0	1.0	1.0	1.0
tr:H7C104:H7C104_HUMAN	0.36	0.36	0.8	0.8	1.0	1.0
tr:E9PEV0:E9PEV0_HUMAN	0.86	0.86	1.0	1.0	1.0	1.0
tr:E9PG40:E9PG40_HUMAN	0.93	0.93	1.0	1.0	1.0	1.0
sp:P05067:A4_HUMAN	1.0	1.0	1.0	1.0	1.0	1.0
sp:P08592:A4_RAT	0.97	0.99	0.99	1.0	1.0	1.0
tr:Q6GR78:Q6GR78_MOUSE	0.88	0.9	0.99	1.0	1.0	1.0
sp:P12023:A4_MOUSE	0.97	0.99	0.99	1.0	1.0	1.0
sp:P14599:A4_DROME	0.2	0.61	0.32	0.73	0.33	0.7
sp:Q10651:A4_CAEEL	0.21	0.54	0.34	0.75	0.33	0.63

Appl (FBgn0000108) associated phenotypes
increased cell death, learning defective, memory defective, neuroanatomy defective, neurophysiology defective, partially lethal - majority die, phototaxis defective, planar polarity defective, progressive, short lived, visual behavior defective

(Information from FlyBase)

Appl (UniProt:P14599) annotation
Function: During development, plays a role in the regulation of the neddylation pathway. Appl and APP-BP1 interact antagonistically during development. (PubMed:16628230).
Subunit: Interacts (via the intracellular domain, ICD) with APP- BP1. (PubMed:16628230).
Subcellular location: Membrane; Single-pass type I membrane protein.
Tissue specificity: Expressed in postmitotic neurons in the central and peripheral nervous systems. Within the nervous system, transcripts are not observed in neuroblasts, newly generated neurons and at least one class of presumed glial cells. (PubMed:2127912, PubMed:2494667).
Developmental stage: Expressed in all developmental stages. (PubMed:2127912, PubMed:2494667).
Domain: The clathrin-binding site is essential for its association with X11-alpha, -beta, and -gamma. The sequence specific recognition extends to peptide residues that are C-terminal to the NPXY motif. This interaction appears to be independent of phosphorylation (By similarity).
Disruption phenotype: Overexpression inhibits the Nedd8 conjugation pathway, disrupts the normal bristle pattern in the fly thorax, and induces apoptosis in wing imaginal disks. (PubMed:16628230).
Caution: Was originally (PubMed:2494667) thought to be vnd butfurther analysis (PubMed:2127912) has clearly shown that it corresponds to Appl. (PubMed:2127912, PubMed:2494667).

(Information from UniProt)

apl-1 (WBGene00000149) associated phenotypes
L1 lethal, body vacuole, cuticle attachment variant, embryonic lethal, larval body morphology variant, larval lethal, molt defect, organism physiology variant, posterior body morphology variant, sodium chloride chemotaxis defective

(Information from WormBase)

apl-1 (UniProt:Q10651) annotation
Subcellular location: Membrane ; Single-pass type I membrane protein

(Information from UniProt)

Myeloperoxidase

Best gene implication in ageing for this target family came from gene D3ZGE2 annotated in UniProt release 2014_02. Annotation GO 7568 (aging) was Inferred from Expression Pattern

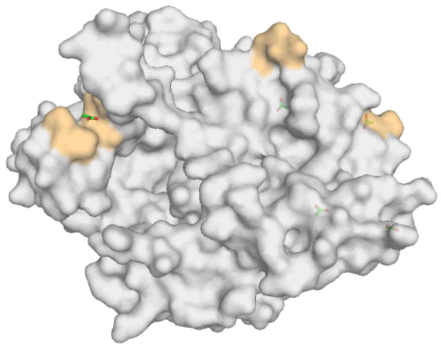

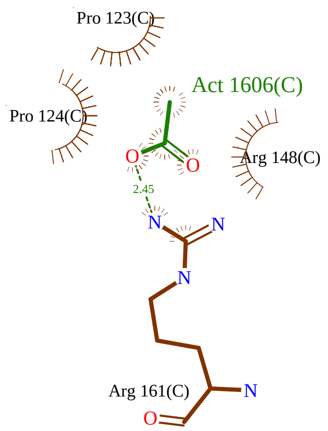

protein	amino acids contacts (binding site)
PDB:1cxp:chainC:P05164	- - - - P P R R M P R D S V R K M R I S R I D L N
PDB:1d2v:chainC:P05164	- - - - P P R R M P R D S V R K M R I S R I D L N
PDB:1d5l:chainC:P05164	- - - - P P R R M P R D S V R K M R I S R I D L N
PDB:1d7w:chainA:P05164	N S L P -
PDB:1d7w:chainC:P05164	- - - - P P R R M P R D S V R K M R I S R I D L N
PDB:3f9p:chainC:P05164	- - - - P P R R M P R D S V R K M R I S R I D L N
PDB:3zs0:chainC:P05164	- - - - P P R R M P R D S V R K M R I S R I D L N
PDB:3zs1:chainC:P05164	- - - - P P R R M P R D S V R K M R I S R I D L N
PDB:4c1m:chainC:P05164	- - - - P P R R M P R D S V R K M R I S R I D L N
sp:P05164:PERM_HUMAN	N S L P P P R R M P R D S V R K M R I S R I D L N
tr:D3ZGE2:D3ZGE2_RAT	- - - - - - - - M P K D S V P N K R I S R I D L N
tr:D3ZYH8:D3ZYH8_RAT	N S L P Q P R R M P K D S V P N - - - - - - E - -
sp:P11247:PERM_MOUSE	N S L P P P R R M P K D S V P N K R I S R I D L N
tr:Q9VEJ9:Q9VEJ9_DROME	N H W G P Y R R I K E S T V P G P L A K R L D I D
sp:Q9VEG6:PERC_DROME	N R W P F A R G E P K V N V E G P L V T R L D V N
tr:O01892:O01892_CAEEL	N K Q T T N R R Q D T T S M A G P I V K K I E I D

	whole protein		domain-based		contact-based	
protein	ident	simil	ident	simil	ident	simil
PDB:1cxp:chainC:P05164	1.0	1.0	1.0	1.0	0.84	0.67
PDB:1d2v:chainC:P05164	1.0	1.0	1.0	1.0	0.84	0.67
PDB:1d5l:chainC:P05164	1.0	1.0	1.0	1.0	0.84	0.67
PDB:1d7w:chainA:P05164	1.0	1.0	1.0	1.0	0.16	0.0
PDB:1d7w:chainC:P05164	1.0	1.0	1.0	1.0	0.84	0.67
PDB:3f9p:chainC:P05164	1.0	1.0	1.0	1.0	0.84	0.67
PDB:3zs0:chainC:P05164	1.0	1.0	1.0	1.0	0.84	0.67
PDB:3zs1:chainC:P05164	1.0	1.0	1.0	1.0	0.84	0.67
PDB:4c1m:chainC:P05164	1.0	1.0	1.0	1.0	0.84	0.67
sp:P05164:PERM_HUMAN	1.0	1.0	1.0	1.0	1.0	1.0
tr:D3ZGE2:D3ZGE2_RAT	0.54	0.59	0.53	0.57	0.52	0.21
tr:D3ZYH8:D3ZYH8_RAT	0.62	0.68	0.79	0.87	0.48	0.21
sp:P11247:PERM_MOUSE	0.83	0.93	0.87	0.96	0.84	0.88
tr:Q9VEJ9:Q9VEJ9_DROME	0.25	0.61	0.33	0.73	0.28	0.57
sp:Q9VEG6:PERC_DROME	0.22	0.52	0.3	0.69	0.32	0.55
tr:O01892:O01892_CAEEL	0.21	0.53	0.28	0.68	0.2	0.52

Pxt (FBgn0261987) associated phenotypes

female semi-sterile

(Information from FlyBase)

Pxt (UniProt:Q9VEG6) annotation

Function: Required for ovarian follicle maturation. Involved in the formation of a rigid and insoluble egg chorion by catalyzing chorion protein cross-linking through dityrosine formation and phenol oxidase-catalyzed chorion melanization. (PubMed:18216169).
Cofactor: heme bNote=Binds 1 heme b (iron(II)-protoporphyrin IX) group per heterodimer. ;
Subunit: Heterodimer. (UniProtKB:P05164,).
Subcellular location: Secreted (PubMed:18216169). Note=In the chorion layer of the mature eggs.
Tissue specificity: Expressed at low levels in the germarium and early follicles. Expression becomes progressively stronger during vitellogenesis, and is highly expressed in germ cells and somatic cells. A subset of follicle cells, termed border cells (BC), exhibit a high level of expression. (PubMed:18216169).
Developmental stage: Expressed both maternally and zygotically. (PubMed:12459925).
Disruption phenotype: Females are sterile, and maturing follicles show defects in actin filament formation, nurse cell membrane stability and border cell migration. (PubMed:18216169).

(Information from UniProt)

Neprilysin

Best gene implication in ageing for this target family came from gene P08473 annotated in UniProt release 2014_02. Annotation GO subterm of 7568 (aging) was Inferred from Expression Pattern

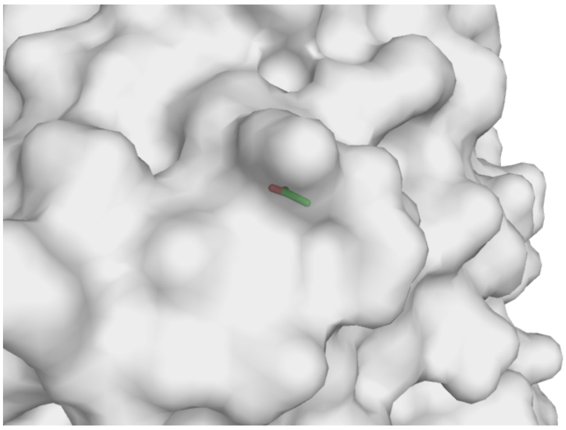

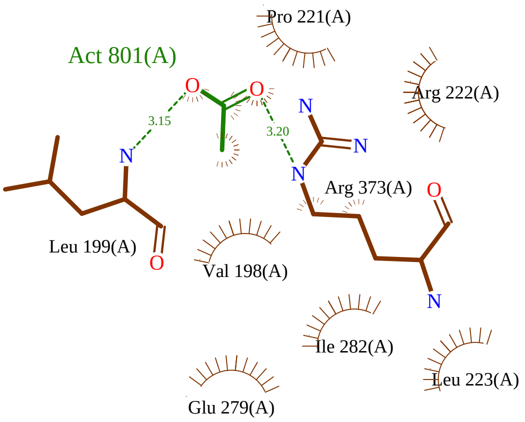

protein	amino acids contacts (binding site)
PDB:1y8j:chainA:P08473	V L P R L E I R
sp:P08473:NEP_HUMAN	V L P R L E I R
sp:P07861:NEP_RAT	V L P R L E I R
sp:Q61391:NEP_MOUSE	V L P R L E I R
tr:Q9XZ01:Q9XZ01_DROME	Y I S S L E L R
tr:Q9W436:Q9W436_DROME	V M L Q Y E L R
tr:Q9I7I4:Q9I7I4_DROME	I L T G L E L R
tr:Q8T062:Q8T062_DROME	I L T G L E L R
tr:O44857:O44857_CAEEL	S L P Q L E L W
sp:Q18673:NEPL1_CAEEL	V F G S L E W R
sp:O16796:NPL11_CAEEL	V F S T L E L R

	whole protein		domain-based		contact-based	
protein	ident	simil	ident	simil	ident	simil
PDB:1y8j:chainA:P08473	1.0	1.0	1.0	1.0	1.0	1.0
sp:P08473:NEP_HUMAN	1.0	1.0	1.0	1.0	1.0	1.0
sp:P07861:NEP_RAT	0.94	0.99	0.92	0.98	1.0	1.0
sp:Q61391:NEP_MOUSE	0.94	0.99	0.92	0.98	1.0	1.0
tr:Q9XZ01:Q9XZ01_DROME	0.32	0.71	0.25	0.72	0.38	0.72
tr:Q9W436:Q9W436_DROME	0.34	0.67	0.34	0.75	0.38	0.78
tr:Q9I7I4:Q9I7I4_DROME	0.27	0.56	0.23	0.55	0.5	0.77
tr:Q8T062:Q8T062_DROME	0.26	0.54	0.23	0.55	0.5	0.77
tr:O44857:O44857_CAEEL	0.35	0.74	0.29	0.71	0.5	0.68
sp:Q18673:NEPL1_CAEEL	0.3	0.7	0.2	0.7	0.5	0.73
sp:O16796:NPL11_CAEEL	0.29	0.67	0.25	0.74	0.5	0.8

Nep4 (FBgn0038818) associated phenotypes

locomotor behavior defective

(Information from FlyBase)

nep-2 (UniProt:O44857) annotation

Function: Required for olfactory plasticity, which is the change from positive chemotaxis to dispersal after prolonged exposure to an odorant. Thought to antagonise snet-1 by degrading excess snet- 1 peptides and thus enabling olfactory plasticity. (PubMed:20929849, PubMed:20929849).
Cofactor: Zn(2+) Evidence=(UniProtKB:P08473); Note=Binds 1 zinc ion per subunit. (UniProtKB:P08473);
Subcellular location: Membrane ; Single-pass type II membrane protein
Tissue specificity: Expressed in muscle cells, GLR cells, SMB motor neurons and AIM interneurons. (PubMed:20929849).

Disruption phenotype: Severe defect in olfactory plasticity in response to the odorants benzaldehyde, isoamylalcohol, diacetyl and pyrazine. (PubMed:20929849).

(Information from UniProt)

nep-1 (UniProt:Q18673) annotation

Function: Probable cell surface protease. Required to control the neuronal innervation of pharyngeal pumping. (PubMed:16081104).
Cofactor: Zn(2+)Note=Binds 1 zinc ion per subunit. ;
Subcellular location: Membrane; Single-pass type II membrane protein.
Tissue specificity: Specifically expressed in pharyngeal cells and a single head neuron. (PubMed:16081104).

(Information from UniProt)

nep-2 (UniProt:O16796) annotation

Function: Probable cell surface protease.
Cofactor: Zn(2+)Note=Binds 1 zinc ion per subunit. ;
Subcellular location: Cell membrane ; Single-pass type II membrane protein

(Information from UniProt)

Heat shock cognate 71 kDa protein

Best gene implication in ageing for this target family came from gene O97125 annotated in UniProt release 2014_02. Annotation GO 8340 (determination of adult lifespan) was Inferred from Mutant Phenotype

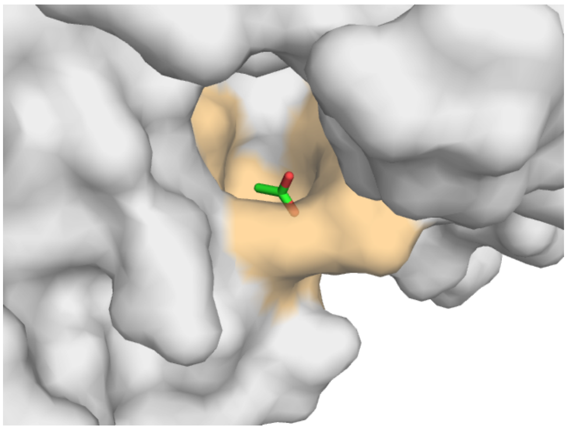

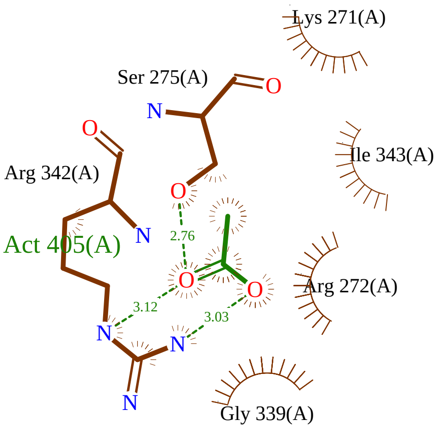

protein	amino acids contacts (binding site)
PDB:4h5v:chainA:P11142	G D T K R S G R I
PDB:4h5w:chainA:P11142	G D T K R S G R I
tr:E9PN89:E9PN89_HUMAN	G D T K R S G R I
tr:E7EP94:E7EP94_HUMAN	G D T K R S G R I
tr:Q96IS6:Q96IS6_HUMAN	G D T K R S G R I
tr:E9PKE3:E9PKE3_HUMAN	G D T K R S G R I
sp:P08107:HSP71_HUMAN	G D T K R S G R I
sp:P11142:HSP7C_HUMAN	G D T K R S G R I
tr:A8K5I0:A8K5I0_HUMAN	G D T K R S G R I
tr:M0R8M9:M0R8M9_RAT	G D T K R S G R I
tr:F1LZI1:F1LZI1_RAT	G D T K R S G R I
tr:M0RCB1:M0RCB1_RAT	G D T K R S G R I
tr:Q3TB63:Q3TB63_MOUSE	G D T K R S G R I
tr:Q3KQJ4:Q3KQJ4_MOUSE	G D T K R S G R I
tr:Q504P4:Q504P4_MOUSE	G D T K R S G R I
sp:P63017:HSP7C_MOUSE	G D T K R S G R I
tr:Q8I0E9:Q8I0E9_DROME	G D T K R S G R I
sp:O97125:HSP68_DROME	G D T K R S G R I
sp:P29843:HSP7A_DROME	G D T K R S G R I
tr:F3YDI6:F3YDI6_DROME	G D T K R S G R I
sp:P82910:HSP70_DROME	G D T K R S G R I
sp:P02825:HSP71_DROME	G D T K R S G R I
tr:C7LA76:C7LA76_DROME	G D T K R S G R I
sp:P29844:HSP7C_DROME	G D T K R S G R I
sp:P11147:HSP7D_DROME	G D T K R S G R I
tr:C7LA75:C7LA75_DROME	G D T K R S G R I
tr:F3YDH0:F3YDH0_DROME	G D T K R S G R I
tr:F3YDH5:F3YDH5_DROME	G D T K R S G R I
tr:Q8IH88:Q8IH88_DROME	G D T K R S G R I
sp:P09446:HSP7A_CAEEL	G D T K R S G R I
sp:P10592:HSP72_YEAST	G D T K R S G R I

protein	whole protein ident	simil	domain-based ident	simil	contact-based ident	simil
PDB:4h5v:chainA:P11142	1.0	1.0	1.0	1.0	1.0	1.0
PDB:4h5w:chainA:P11142	1.0	1.0	1.0	1.0	1.0	1.0
tr:E9PN89:E9PN89_HUMAN	0.48	0.48	0.93	0.93	1.0	1.0
tr:E7EP94:E7EP94_HUMAN	0.72	0.81	0.9	0.97	1.0	1.0
tr:Q96IS6:Q96IS6_HUMAN	0.91	0.91	1.0	1.0	1.0	1.0
tr:E9PKE3:E9PKE3_HUMAN	0.97	0.97	1.0	1.0	1.0	1.0
sp:P08107:HSP71_HUMAN	0.84	0.94	0.9	0.97	1.0	1.0
sp:P11142:HSP7C_HUMAN	1.0	1.0	1.0	1.0	1.0	1.0
tr:A8K5I0:A8K5I0_HUMAN	0.84	0.94	0.9	0.97	1.0	1.0
tr:M0R8M9:M0R8M9_RAT	0.99	1.0	0.98	0.99	1.0	1.0
tr:F1LZI1:F1LZI1_RAT	1.0	1.0	1.0	1.0	1.0	1.0
tr:M0RCB1:M0RCB1_RAT	0.98	0.99	0.99	1.0	1.0	1.0
tr:Q3TB63:Q3TB63_MOUSE	0.71	0.71	1.0	1.0	1.0	1.0
tr:Q3KQJ4:Q3KQJ4_MOUSE	0.87	0.87	1.0	1.0	1.0	1.0
tr:Q504P4:Q504P4_MOUSE	0.97	0.97	1.0	1.0	1.0	1.0
sp:P63017:HSP7C_MOUSE	1.0	1.0	1.0	1.0	1.0	1.0
tr:Q8I0E9:Q8I0E9_DROME	0.68	0.8	0.88	0.98	1.0	1.0
sp:O97125:HSP68_DROME	0.7	0.89	0.81	0.94	1.0	1.0
sp:P29843:HSP7A_DROME	0.8	0.93	0.88	0.98	1.0	1.0
tr:F3YDI6:F3YDI6_DROME	0.73	0.9	0.8	0.93	1.0	1.0
sp:P82910:HSP70_DROME	0.73	0.9	0.8	0.93	1.0	1.0
sp:P02825:HSP71_DROME	0.73	0.9	0.8	0.93	1.0	1.0
tr:C7LA76:C7LA76_DROME	0.73	0.9	0.79	0.93	1.0	1.0
sp:P29844:HSP7C_DROME	0.59	0.83	0.64	0.9	1.0	1.0
sp:P11147:HSP7D_DROME	0.84	0.95	0.9	0.98	1.0	1.0
tr:C7LA75:C7LA75_DROME	0.84	0.95	0.9	0.98	1.0	1.0
tr:F3YDH0:F3YDH0_DROME	0.59	0.83	0.64	0.9	1.0	1.0
tr:F3YDH5:F3YDH5_DROME	0.77	0.9	0.88	0.98	1.0	1.0
tr:Q8IH88:Q8IH88_DROME	0.75	0.88	0.88	0.98	1.0	1.0
sp:P09446:HSP7A_CAEEL	0.84	0.94	0.9	0.97	1.0	1.0
sp:P10592:HSP72_YEAST	0.75	0.92	0.85	0.96	1.0	1.0

Hsc70-1 (FBgn0001216) associated phenotypes
partially lethal - majority die, some die during pupal stage

(Information from FlyBase)

Hsp68 (FBgn0001230) associated phenotypes
chemical resistant, long lived, neuroanatomy defective, partially lethal - majority die, some die during pupal stage, starvation stress response defective

(Information from FlyBase)

Hsc70-1 (UniProt:P29843) annotation
Developmental stage: Heat shock cognate proteins are expressed constitutively during normal development.

(Information from UniProt)

Hsp70Aa (UniProt:P82910) annotation
Induction: Heat shock induces the synthesis of seven proteins at five otherwise inactive sites in the polytene chromosomes of fruit fly larvae. Two separate sites, producing two and three copies, respectively, code for the 70 kDa protein.
Miscellaneous: There are two copies of the gene coding for this protein at chromosome locus 87A7.

(Information from UniProt)

Hsp70Ab (UniProt:P02825) annotation
Induction: Heat shock induces the synthesis of seven proteins at five otherwise inactive sites in the polytene chromosomes of fruit fly larvae. Two separate sites, producing two and three copies, respectively, code for the 70 kDa protein.
Miscellaneous: There are two copies of the gene coding for this protein at chromosome locus 87A7.

(Information from UniProt)

Hsc70-3 (FBgn0001218) associated phenotypes
dominant, neuroanatomy defective, partially lethal - majority die

(Information from FlyBase)

Hsc70-3 (UniProt:P29844) annotation
Function: Probably plays a role in facilitating the assembly of multimeric protein complexes inside the ER.
Subcellular location: Endoplasmic reticulum lumen.

(Information from UniProt)

Hsc70-4 (UniProt:P11147) annotation
Subcellular location: Cytoplasm, perinuclear region. Nucleus. Note=Localized to a meshwork of cytoplasmic fibers around the nucleus. Translocates to the nucleus after thermal stress.
Developmental stage: Heat shock cognate proteins are expressed constitutively during normal development.

(Information from UniProt)

hsp-1 (WBGene00002005) associated phenotypes
lethal, sterile

(Information from WormBase)

Fibroblast growth factor receptor 1

Best gene implication in ageing for this target family came from gene P11362 via mapping the annotation from Ensembl ENSG00000077782 via mapping the annotation from EntrezGene 2260 via mapping the annotation from GenAgeHuman 0169 annotated in GenAge release 17.

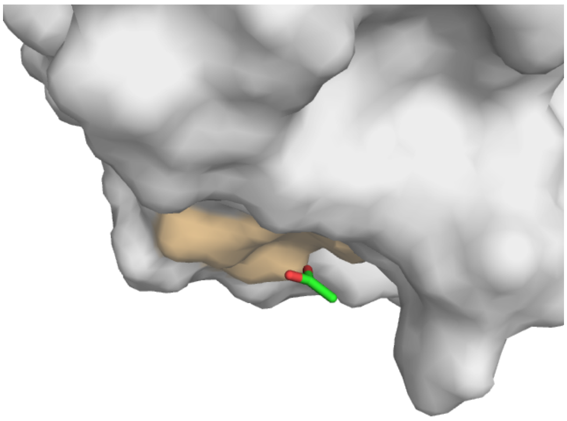

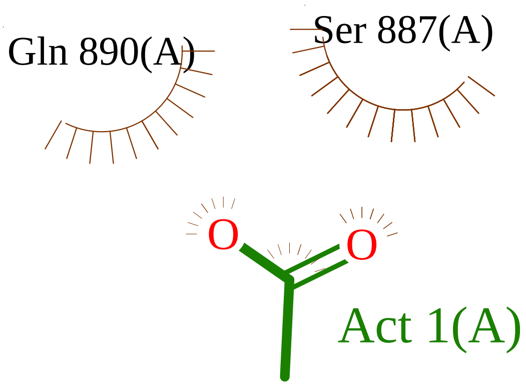

protein	amino acids contacts (binding site)		
PDB:3krj:chainA:P11362	S	Q	A
tr:D3DSX2:D3DSX2_HUMAN	M	R	D
tr:E7EU09:E7EU09_HUMAN	M	R	D
tr:J3KNT4:J3KNT4_HUMAN	M	R	D
sp:P11362:FGFR1_HUMAN	M	R	D
tr:Q63827:Q63827_RAT	M	R	D
tr:F1LM54:F1LM54_RAT	M	R	D
tr:Q8CBY7:Q8CBY7_MOUSE	M	R	D
sp:P16092:FGFR1_MOUSE	M	R	D
tr:Q8CIM9:Q8CIM9_MOUSE	M	R	D
tr:J3QN85:J3QN85_MOUSE	M	R	D
sp:Q07407:FGFR1_DROME	I	R	Q
sp:Q09147:FGFR2_DROME	V	R	Q
sp:Q10656:EGL15_CAEEL	H	C	S

	whole protein		domain-based		contact-based	
protein	ident	simil	ident	simil	ident	simil
PDB:3krj:chainA:P11362	0.5	0.83	0.54	0.84	1.0	1.0
tr:D3DSX2:D3DSX2_HUMAN	0.47	0.47	1.0	1.0	0.0	0.37
tr:E7EU09:E7EU09_HUMAN	0.85	0.87	1.0	1.0	0.0	0.37
tr:J3KNT4:J3KNT4_HUMAN	0.96	0.97	1.0	1.0	0.0	0.37
sp:P11362:FGFR1_HUMAN	1.0	1.0	1.0	1.0	0.0	0.37
tr:Q63827:Q63827_RAT	0.88	0.88	1.0	1.0	0.0	0.37
tr:F1LM54:F1LM54_RAT	0.98	1.0	1.0	1.0	0.0	0.37
tr:Q8CBY7:Q8CBY7_MOUSE	0.42	0.42	1.0	1.0	0.0	0.37
sp:P16092:FGFR1_MOUSE	0.98	1.0	1.0	1.0	0.0	0.37
tr:Q8CIM9:Q8CIM9_MOUSE	0.98	0.99	1.0	1.0	0.0	0.37
tr:J3QN85:J3QN85_MOUSE	0.97	0.98	1.0	1.0	0.0	0.37
sp:Q07407:FGFR1_DROME	0.3	0.61	0.57	0.85	0.0	0.34
sp:Q09147:FGFR2_DROME	0.26	0.54	0.57	0.84	0.0	0.42
sp:Q10656:EGL15_CAEEL	0.22	0.49	0.5	0.78	0.0	0.28

htl (FBgn0010389) associated phenotypes

cell migration defective, increased cell death, increased cell number, neuroanatomy defective, somatic clone

(Information from FlyBase)

htl (UniProt:Q07407) annotation

Function: May be required for patterning of muscle precursor cells. May be essential for generation of mesodermal and endodermal layers, invaginations of various types of cells and CNS formation. (PubMed:8330538).
Subcellular location: Membrane; Single-pass type I membrane protein.
Tissue specificity: In early embryos, expression is specific to mesodermal primordium and invaginated mesodermal cells. At later stages, expression is seen in putative muscle precursor cells and in the CNS. (PubMed:8330538).
Developmental stage: Embryogenesis. (PubMed:8330538).
Caution: It is uncertain whether Met-1 or Met-16 is the initiator.

(Information from UniProt)

btl (FBgn0005592) associated phenotypes

cell migration defective, neuroanatomy defective, somatic clone

(Information from FlyBase)

btl (UniProt:Q09147) annotation

Function: May be required for patterning of muscle precursor cells: generation of mesodermal and endodermal layers, invaginations of various types of cells, and CNS formation. Essential for the ability of the migrating tracheal and midline cells to recognize external guiding cues. (PubMed:1325393, PubMed:1849109, PubMed:8330538).

Subcellular location: Membrane; Single-pass type I membrane protein.
Tissue specificity: During embryogenesis, expression is seen in mesoderm, endodermal precursor cells, CNS midline cells and trachea and salivary duct ectodermal cells. (PubMed:1325393, PubMed:1849109, PubMed:8330538).
Developmental stage: All stages of development. (PubMed:1849109).

(Information from UniProt)

egl-15 (WBGene00001184) associated phenotypes

L1 arrest, SM migration variant, axon midline crossing variant, bag of worms, bloated, egg laying defective, egg laying imipramine resistant, egg laying levamisole resistant, egg laying phentolamine resistant, egg laying serotonin resistant, egg laying variant, hermaphrodite sex muscle development variant, hermaphrodite sex muscle morphology variant, hermaphrodite sexual development variant, imipramine resistant, larval arrest, larval lethal, scrawny, serotonin resistant, ventral nerve cord maintenance variant

(Information from WormBase)

egl-15 (UniProt:Q10656) annotation

Function: Activation leads to the phosphorylation of SOC-1 on Tyr- 408 triggering PTP-2 recruitment and/or activation. Activates the SEM-5/SOS-1/RAS/MAPK pathway (PubMed:11689700). Acts in the hypodermis to regulate axon growth (PubMed:12835392). Activates protein degradation in muscles (PubMed:14517244). Plays a role in fluid homeostasis by acting in the hypodermis and also has a role in larval development (PubMed:7585964). Isoform b has no effect on axon growth but affects the maintenance of axon position. Isoform b interaction with egl-17 is required for the guidance of sex myoblast migration during gonad development (PubMed:12835392). Isoforms a, c, d and e interaction with let-756 appears to play a role in maintaining body morphology at higher temperatures (PubMed:12835392). (PubMed:11689700, PubMed:12835392, PubMed:14517244, PubMed:7585964).
Cofactor: Mn(2+)
Subcellular location: Membrane; Single-pass type I membrane protein.
Developmental stage: Expressed in the hypodermal membrane during the larval stage. (PubMed:11689700).
Ptm: Activity is regulated by the phosphatase clr-1, however it is not known whether clr-1 acts directly on egl-15. (PubMed:9585503).
Disruption phenotype: Early arrest in larval development. Impaired guided migration of sex myoblasts. (PubMed:7585964).

(Information from UniProt)

Angiotensin-converting enzyme

Best gene implication in ageing for this target family came from gene M0RB66 via mapping the annotation from Ensembl ENSRNOG00000007467 via mapping the annotation from RGD 2493 annotated in RGD 2014-03-11. Annotation GO 7568 (aging) was Inferred from Expression Pattern

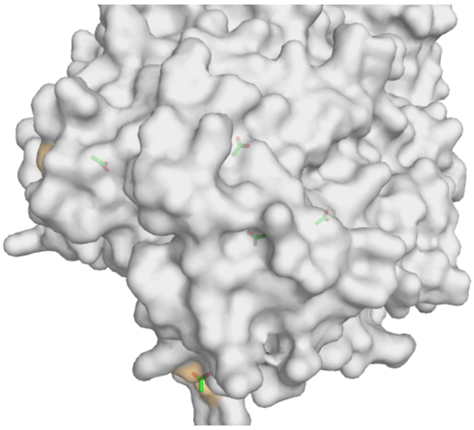

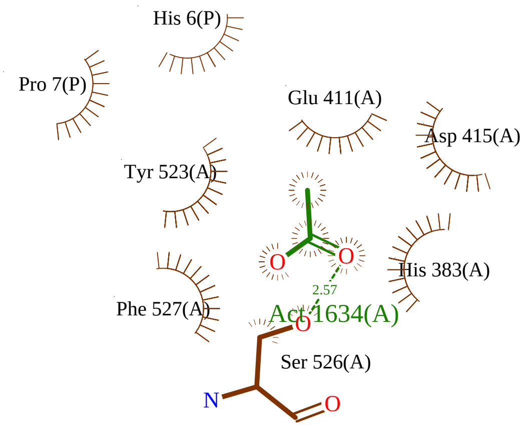

protein	amino acids contacts (binding site)																												
PDB:2c6f:chainA:P12821	S	A	S	N	L	Y	L	L	M	F	W	Q	Y	H	H	E	D	S	N	Y	R	D	K	F	H	Y	Y	S	F
PDB:4aph:chainA:P12821	Y	N	R	N	L	Y	H	L	D	W	W	Q	Y	H	H	E	D	H	N	F	S	E	K	F	H	Y	Y	S	F
tr:L7MUH0:L7MUH0_HUMAN	Y	N	R	N	L	Y	H	L	D	W	W	Q	Y	H	H	E	D	H	N	F	S	E	K	F	H	Y	Y	S	F
sp:P12821:ACE_HUMAN	Y	N	R	N	L	Y	H	L	D	W	W	Q	Y	H	H	E	D	H	N	F	S	E	K	F	H	Y	Y	S	F
tr:M0RB66:M0RB66_RAT	L	E	K	N	L	Y	L	L	E	W	W	Q	L	H	H	E	S	E	N	F	T	E	K	F	H	Y	Y	S	L
tr:Q8K233:Q8K233_MOUSE	Y	S	R	N	L	Y	Y	L	E	W	W	Q	Y	H	H	E	D	Y	N	F	S	E	K	F	H	Y	Y	S	F
tr:Q3TU20:Q3TU20_MOUSE	Y	S	R	N	L	Y	Y	L	E	W	W	Q	Y	H	H	E	D	Y	N	F	S	E	K	F	H	Y	Y	S	F
sp:P09470:ACE_MOUSE	Y	S	R	N	L	Y	Y	L	E	W	W	Q	Y	H	H	E	D	Y	N	F	S	E	K	F	H	Y	Y	S	F
sp:Q10714:ACE_DROME	E	S	Y	L	L	F	D	L	E	Y	W	Q	A	H	H	E	D	A	N	Q	E	A	K	Y	H	Y	Y	S	F
sp:Q9VLJ6:ACER_DROME	E	Y	A	Q	I	Y	S	L	E	W	W	Q	L	H	H	E	D	S	N	Q	E	G	K	Y	H	Y	Y	A	H
sp:Q18581:ACN1_CAEEL	V	E	M	S	Q	S	D	Q	R	H	D	G	Y	H	S	D	N	D	N	K	T	R	H	N	S	A	T	S	Y

	whole protein		domain-based		contact-based	
protein	ident	simil	ident	simil	ident	simil
PDB:2c6f:chainA:P12821	0.55	0.84	0.56	0.85	1.0	1.0
PDB:4aph:chainA:P12821	1.0	1.0	1.0	1.0	0.66	0.69
tr:L7MUH0:L7MUH0_HUMAN	0.45	0.49	1.0	1.0	0.66	0.69
sp:P12821:ACE_HUMAN	1.0	1.0	1.0	1.0	0.66	0.69
tr:M0RB66:M0RB66_RAT	0.26	0.42	0.55	0.86	0.59	0.71
tr:Q8K233:Q8K233_MOUSE	0.65	0.74	0.82	0.96	0.66	0.71
tr:Q3TU20:Q3TU20_MOUSE	0.83	0.95	0.82	0.96	0.66	0.71
sp:P09470:ACE_MOUSE	0.83	0.95	0.82	0.96	0.66	0.71
sp:Q10714:ACE_DROME	0.2	0.37	0.45	0.8	0.52	0.56
sp:Q9VLJ6:ACER_DROME	0.18	0.37	0.41	0.79	0.48	0.62
sp:Q18581:ACN1_CAEEL	0.13	0.41	0.26	0.72	0.14	0.42

Ance (FBgn0012037) associated phenotypes

partially lethal - majority live

(Information from FlyBase)

Ance (UniProt:Q10714) annotation

Function: May be involved in the specific maturation or degradation of a number of bioactive peptides. May play a role in the contractions of the heart, gut and testes, and in spermatid differentiation. (PubMed:12591244).

Cofactor: Zn(2+)Note=Binds 1 zinc ion per subunit.;

Enzyme regulation: Inhibited by captopril and, to a lesser extent, by lisinopril, trandolaprilat, fosinoprilat and enalaprilat. (PubMed:7775412, PubMed:8761461).

Biophysicochemical properties: Kinetic parameters: KM=33.5 uM for angiotensin I (PubMed:7775412, PubMed:8761461, PubMed:9839949); KM=53.4 uM for N-acetyl-Ser-Asp-Lys-Pro (PubMed:7775412, PubMed:8761461, PubMed:9839949); KM=2.59 mM for Hip-His-Leu (PubMed:7775412, PubMed:8761461, PubMed:9839949); KM=10.26 mM for Hip-His-Leu-NH(2) (PubMed:7775412, PubMed:8761461, PubMed:9839949); KM=372 uM for (Leu5)enkephalin (PubMed:7775412, PubMed:8761461, PubMed:9839949); KM=1.88 mM for (Leu5)enkephalinamide (PubMed:7775412, PubMed:8761461, PubMed:9839949);

Subcellular location: Secreted, extracellular space.
Tissue specificity: Expressed in vesicular structures in spermatocytes and early spermatids (at protein level). (PubMed:12591244).
Developmental stage: Expressed in the amnioserosa during germ band elongation, shortening and heart morphogenesis. Expressed in midgut throughout embryogenesis.
Ptm: Glycosylated. (PubMed:8761461).
Disruption phenotype: Male flies are sterile. (PubMed:12591244).

(Information from UniProt)

Acer (FBgn0016122) associated phenotypes
circadian behavior defective, short lived, sleep defective

(Information from FlyBase)

Acer (UniProt:Q9VLJ6) annotation
Function: May be involved in the specific maturation or degradation of a number of bioactive peptides. May have a role in the specification of heart progenitors. (PubMed:12075344).
Cofactor: Zn(2+)Note=Binds 1 zinc ion per subunit. ;
Enzyme regulation: Inhibited by captopril, lisinopril, trandolaprilat, fosinoprilat and enalaprilat. (PubMed:9839949).
Biophysicochemical properties: Kinetic parameters: KM=1.2 mM for Hip-His-Leu (PubMed:9839949); KM=4.35 mM for Hip-His-Leu-NH(2) (PubMed:9839949); KM=40.6 uM for (Leu5)enkephalin (PubMed:9839949); KM=949 uM for (Leu5)enkephalinamide (PubMed:9839949); pH dependence: Optimum pH is 8.6. (PubMed:9839949);
Subcellular location: Secreted, extracellular space (PubMed:9839949).
Developmental stage: Expressed in presumptive heart cells during dorsal closure. (PubMed:8973330).
Ptm: Glycosylated. (PubMed:9839949).
Disruption phenotype: Defective heart morphogenesis leading to lethality. (PubMed:12075344).
Miscellaneous: In contrast to ance, does not hydrolyze angiotensin I.

(Information from UniProt)

acn-1 (WBGene00000039) associated phenotypes
embryonic lethal, lethal, sterile

(Information from WormBase)

acn-1 (UniProt:Q18581) annotation
Function: Required for larval molting, male tail development, and formation of adult alae. Inactive as a metallopeptidase, due to a lack of active site residues. (PubMed:14559923).
Tissue specificity: Expressed in embryonic and larval hypodermis, in the vulva during organogenesis, and in the ray papillae of the male tail. (PubMed:14559923).

(Information from UniProt)

Insulin-degrading enzyme

Best gene implication in ageing for this target family came from gene P14735 annotated in UniProt release 2014_02. Annotation GO 8340 (determination of adult lifespan) was Inferred from Direct Assay

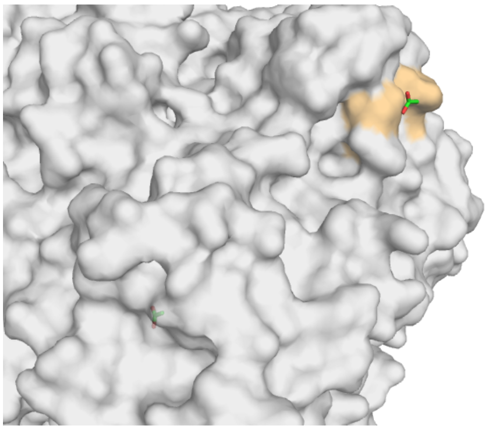

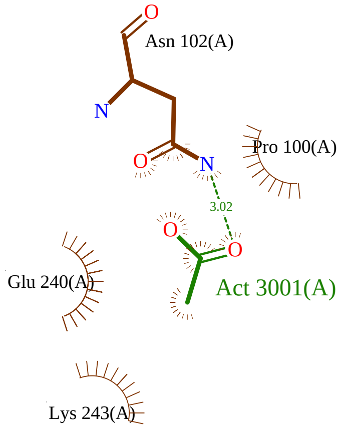

protein	amino acids contacts (binding site)
PDB:3cww:chainA:P14735	P N E K N T V N
sp:P14735:IDE_HUMAN	P N E K N T V N
sp:P35559:IDE_RAT	P N E K N T V N
sp:P22817:IDE_DROME	P N E K S D M V
tr:Q17593:Q17593_CAEEL	P E A E L S K T
sp:Q10040:YQA4_CAEEL	P E V D Q S E T
tr:Q6A598:Q6A598_CAEEL	P E A Q L S Q T
tr:G5ED87:G5ED87_CAEEL	P E A Q Q S Q G
tr:Q8IA89:Q8IA89_CAEEL	P E A Q L S Q T
tr:Q8MXK1:Q8MXK1_CAEEL	P E A Q L S Q T
tr:O16249:O16249_CAEEL	P E A Q L S Q T
sp:Q06010:STE23_YEAST	P N E K K E S D

	whole protein		domain-based		contact-based	
protein	ident	simil	ident	simil	ident	simil
PDB:3cww:chainA:P14735	0.97	0.98	0.98	0.99	1.0	1.0
sp:P14735:IDE_HUMAN	1.0	1.0	1.0	1.0	1.0	1.0
sp:P35559:IDE_RAT	0.95	0.99	0.96	0.99	1.0	1.0
sp:P22817:IDE_DROME	0.44	0.79	0.53	0.85	0.5	0.67
tr:Q17593:Q17593_CAEEL	0.32	0.61	0.47	0.81	0.13	0.51
sp:Q10040:YQA4_CAEEL	0.31	0.65	0.39	0.69	0.13	0.53
tr:Q6A598:Q6A598_CAEEL	0.37	0.71	0.5	0.83	0.13	0.51
tr:G5ED87:G5ED87_CAEEL	0.37	0.75	0.48	0.82	0.13	0.6
tr:Q8IA89:Q8IA89_CAEEL	0.38	0.74	0.5	0.83	0.13	0.51
tr:Q8MXK1:Q8MXK1_CAEEL	0.38	0.76	0.5	0.83	0.13	0.51
tr:O16249:O16249_CAEEL	0.38	0.75	0.5	0.83	0.13	0.51
sp:Q06010:STE23_YEAST	0.36	0.76	0.46	0.83	0.5	0.64

Ide (FBgn0001247) associated phenotypes

decreased cell number, decreased cell size, developmental rate defective, large body, long lived, nutrition conditional, partially lethal - majority die, short lived, size defective, small body

(Information from FlyBase)

Ide (UniProt:P22817) annotation

Function: Can cleave insulin and TGF-alpha. (PubMed:3139025).
Cofactor: Zn(2+)Note=Binds 1 zinc ion per subunit. ;
Biophysicochemical properties: pH dependence: Optimum pH is 7-8. (PubMed:3139025); Temperature dependence: Optimum temperature is 37 degrees Celsius. (PubMed:3139025);
Induction: Inhibited by bacitracin and sulfhydryl-specific reagents. (PubMed:3139025).

(Information from UniProt)

DNA polymerase delta catalytic subunit

Best gene implication in ageing for this target family came from gene M0QZR8 via mapping the annotation from Ensembl ENSG00000062822 via mapping the annotation from EntrezGene 5424 via mapping the annotation from GenAgeHuman 0118 annotated in GenAge release 17.

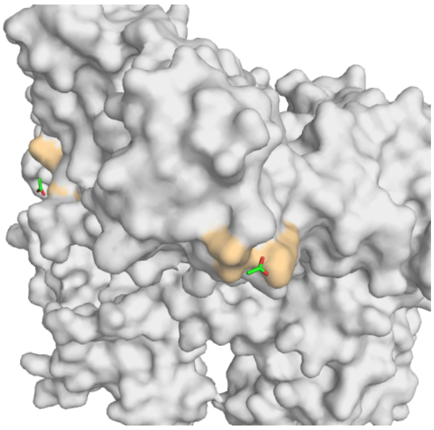

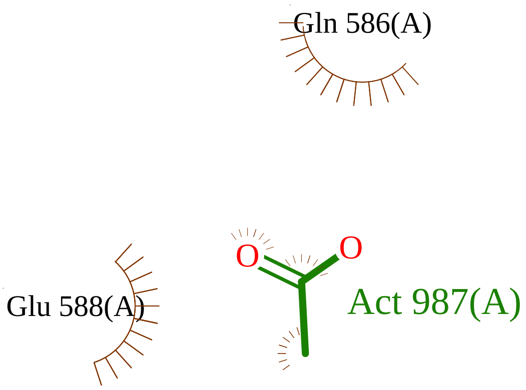

protein	amino acids contacts (binding site)
PDB:3iay:chainA:P15436	E H Q E
tr:M0QZR8:M0QZR8_HUMAN	E P D T
sp:P28340:DPOD1_HUMAN	E P D T
tr:M0R2B7:M0R2B7_HUMAN	E P D T
tr:G3V8M1:G3V8M1_RAT	E P D T
tr:D6RFB8:D6RFB8_MOUSE	E P D T
sp:P52431:DPOD1_MOUSE	E P D T
sp:P54358:DPOD1_DROME	D E Q E
sp:P90829:DPOD1_CAEEL	Q K G E
sp:P15436:DPOD_YEAST	E H Q E

	whole protein		domain-based		contact-based	
protein	ident	simil	ident	simil	ident	simil
PDB:3iay:chainA:P15436	1.0	1.0	1.0	1.0	1.0	1.0
tr:M0QZR8:M0QZR8_HUMAN	0.38	0.64	0.42	0.75	0.25	0.67
sp:P28340:DPOD1_HUMAN	0.46	0.76	0.42	0.75	0.25	0.67
tr:M0R2B7:M0R2B7_HUMAN	0.45	0.74	0.38	0.68	0.25	0.67
tr:G3V8M1:G3V8M1_RAT	0.45	0.76	0.4	0.75	0.25	0.67
tr:D6RFB8:D6RFB8_MOUSE	0.38	0.65	0.41	0.75	0.25	0.67
sp:P52431:DPOD1_MOUSE	0.45	0.76	0.41	0.75	0.25	0.67
sp:P54358:DPOD1_DROME	0.48	0.76	0.42	0.75	0.5	0.8
sp:P90829:DPOD1_CAEEL	0.42	0.73	0.32	0.7	0.25	0.72
sp:P15436:DPOD_YEAST	1.0	1.0	1.0	1.0	1.0	1.0

DNApol-delta (FBgn0263600) associated phenotypes

neuroanatomy defective, partially lethal - majority die, some die during pupal stage

(Information from FlyBase)

DNApol-delta (UniProt:P54358) annotation

Function: Possesses two enzymatic activities: DNA synthesis (polymerase) and an exonucleolytic activity that degrades single stranded DNA in the 3'- to 5'-direction. Required with its accessory proteins (proliferating cell nuclear antigen (PCNA) and replication factor C (RFC) or activator 1) for leading strand synthesis. Also involved in completing Okazaki fragments initiated by the DNA polymerase alpha/primase complex (By similarity).
Cofactor: [4Fe-4S] cluster Evidence=; Note=Binds 1 [4Fe-4S] cluster. ;
Subunit: Heterodimer with subunits of 125 kDa and 50 kDa. The 125 kDa subunit contains the polymerase active site and most likely the active site for the 3'-5' exonuclease activity (By similarity).

Subcellular location: Nucleus.
Domain: The CysB motif binds 1 4Fe-4S cluster and is required for the formation of polymerase complexes.
Miscellaneous: In eukaryotes there are five DNA polymerases: alpha, beta, gamma, delta, and epsilon which are responsible for different reactions of DNA synthesis.

(Information from UniProt)

F10C2.4 (UniProt:P90829) annotation
Function: Possesses two enzymatic activities: DNA synthesis (polymerase) and an exonucleolytic activity that degrades single stranded DNA in the 3'- to 5'-direction. Required with its accessory proteins (proliferating cell nuclear antigen (PCNA) and replication factor C (RFC) or activator 1) for leading strand synthesis. Also involved in completing Okazaki fragments initiated by the DNA polymerase alpha/primase complex (By similarity).
Cofactor: [4Fe-4S] cluster Evidence=; Note=Binds 1 [4Fe-4S] cluster. ;
Subunit: Heterodimer with subunits of 125 kDa and 50 kDa. The 125 kDa subunit contains the polymerase active site and most likely the active site for the 3'-5' exonuclease activity (By similarity).
Subcellular location: Nucleus
Domain: The CysB motif binds 1 4Fe-4S cluster and is required for the formation of polymerase complexes.
Miscellaneous: In eukaryotes there are five DNA polymerases: alpha, beta, gamma, delta, and epsilon which are responsible for different reactions of DNA synthesis.

(Information from UniProt)

2',3'-cyclic-nucleotide 3'-phosphodiesterase

Best gene implication in ageing for this target family came from gene P13233 annotated in UniProt release 2014_02. Annotation GO 7568 (aging) was Inferred from Expression Pattern

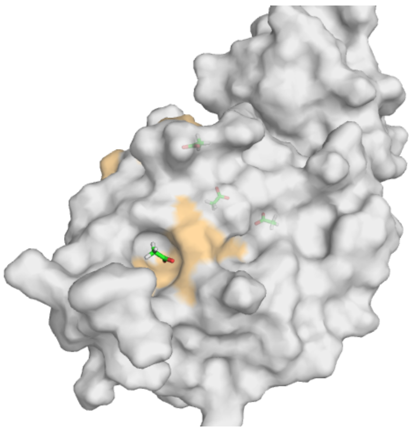

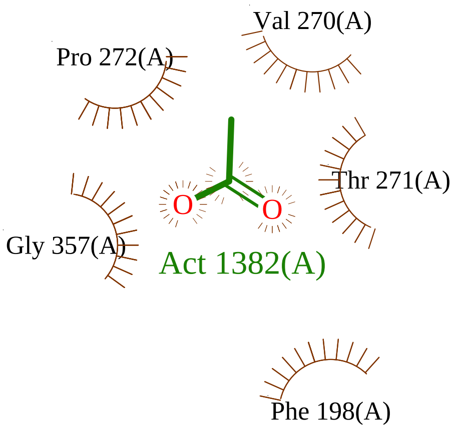

protein	amino acids contacts (binding site)													
PDB:2yoz:chainA:P16330	F	K	R	P	A	K	V	T	P	T	G	C	G	K
PDB:2yph:chainA:P16330	F	K	R	P	A	K	V	T	P	T	G	C	G	K
sp:P09543:CN37_HUMAN	F	K	R	P	A	T	V	T	P	T	G	C	G	R
sp:P13233:CN37_RAT	F	K	R	P	A	K	V	T	P	T	G	C	G	K
tr:Q3TYL9:Q3TYL9_MOUSE	F	K	R	P	A	K	V	T	P	T	G	C	G	K
sp:P16330:CN37_MOUSE	F	K	R	P	A	K	V	T	P	T	G	C	G	K
tr:Q3TYV5:Q3TYV5_MOUSE	F	K	R	P	A	K	V	T	P	T	G	C	G	K
tr:Q19804:Q19804_CAEEL	-	-	-	-	-	-	-	-	-	-	-	-	-	-

protein	whole protein ident	simil	domain-based ident	simil	contact-based ident	simil
PDB:2yoz:chainA:P16330	1.0	1.0	1.0	1.0	1.0	1.0
PDB:2yph:chainA:P16330	1.0	1.0	0.99	1.0	1.0	1.0
sp:P09543:CN37_HUMAN	0.85	0.96	0.8	0.95	0.86	0.93
sp:P13233:CN37_RAT	0.95	0.99	0.95	0.99	1.0	1.0
tr:Q3TYL9:Q3TYL9_MOUSE	0.95	0.95	1.0	1.0	1.0	1.0
sp:P16330:CN37_MOUSE	1.0	1.0	1.0	1.0	1.0	1.0
tr:Q3TYV5:Q3TYV5_MOUSE	1.0	1.0	1.0	1.0	1.0	1.0
tr:Q19804:Q19804_CAEEL	0.11	0.28	0.02	0.05	0.0	0.0

Tyrosine-protein phosphatase non-receptor type 1

Best gene implication in ageing for this target family came from gene P18031 via mapping the annotation from Ensembl ENSG00000196396 via mapping the annotation from EntrezGene 5770 via mapping the annotation from GenAgeHuman 0033 annotated in GenAge release 17.

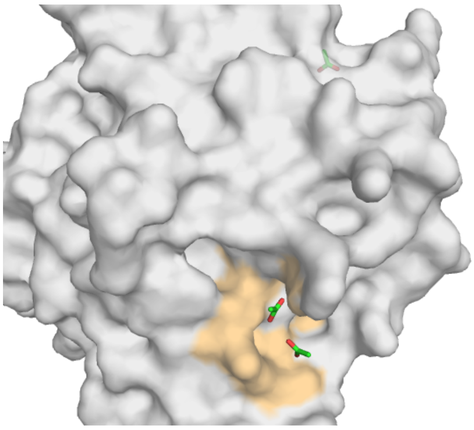

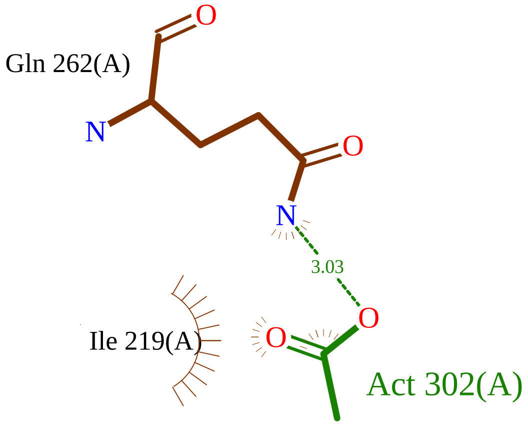

protein	amino acids contacts (binding site)							
PDB:1ecv:chainA:P18031	R	D	H	W	E	I	R	Q
sp:P18031:PTN1_HUMAN	R	D	H	W	E	I	R	Q
tr:A8K3M3:A8K3M3_HUMAN	R	D	H	W	E	I	R	Q
sp:P20417:PTN1_RAT	R	D	H	W	E	I	R	Q
tr:Q3TB93:Q3TB93_MOUSE	R	D	H	W	E	I	R	Q
tr:Q3TZW9:Q3TZW9_MOUSE	R	D	H	W	E	I	R	Q
sp:P35821:PTN1_MOUSE	R	D	H	W	E	I	R	Q
sp:Q9W0G1:PTP61_DROME	E	D	K	W	E	I	R	Q
tr:D3DMI1:D3DMI1_DROME	E	D	K	W	E	I	R	Q
tr:I0B1P6:I0B1P6_DROME	E	D	K	W	E	I	R	Q

protein	whole protein ident	simil	domain-based ident	simil	contact-based ident	simil
PDB:1ecv:chainA:P18031	0.99	1.0	0.99	1.0	1.0	1.0
sp:P18031:PTN1_HUMAN	1.0	1.0	1.0	1.0	1.0	1.0
tr:A8K3M3:A8K3M3_HUMAN	1.0	1.0	1.0	1.0	1.0	1.0
sp:P20417:PTN1_RAT	0.84	0.94	0.96	0.99	1.0	1.0
tr:Q3TB93:Q3TB93_MOUSE	0.7	0.73	0.95	0.99	1.0	1.0
tr:Q3TZW9:Q3TZW9_MOUSE	0.83	0.94	0.95	0.99	1.0	1.0
sp:P35821:PTN1_MOUSE	0.83	0.94	0.95	0.99	1.0	1.0
sp:Q9W0G1:PTP61_DROME	0.3	0.59	0.49	0.8	0.75	0.9
tr:D3DMI1:D3DMI1_DROME	0.3	0.59	0.49	0.8	0.75	0.9
tr:I0B1P6:I0B1P6_DROME	0.3	0.59	0.49	0.8	0.75	0.9

Ptp61F (UniProt:Q9W0G1) annotation

Function: Non-receptor protein tyrosine phosphatase required for maintaining Dock in its non-phosphorylated state. (PubMed:12014990, PubMed:8463208).

Subcellular location: Isoform A: Cytoplasm. Endomembrane system. Note=Associates with the

membranes of the reticular network and the mitochondria.
Subcellular location: Isoform B: Nucleus.
Tissue specificity: Expressed during oogenesis and embryogenesis. Isoform A and isoform B are expressed in distinct patterns. Isoform B is expressed in the mesoderm and neuroblast layer during germband extension and later in the gut epithelia. Isoform A accumulates in 16 segmentally repeated stripes in the ectoderm during germband extension. These stripes are flanked by, and adjacent to, the domains of engrailed and wingless gene expression in the anterior/posterior axis. In stage 10 embryos, isoform A colocalizes with the area lateral to the denticle belts that will give rise to naked cuticle. Isoform A is also expressed later in embryogenesis in the central nervous system. (PubMed:9256342).

(Information from UniProt)

Retinoic acid receptor RXR-alpha

Best gene implication in ageing for this target family came from gene Q0VJ96 via mapping the annotation from RGD 3610 annotated in RGD 2014-03-11. Annotation GO 7568 (aging) was Inferred from Expression Pattern

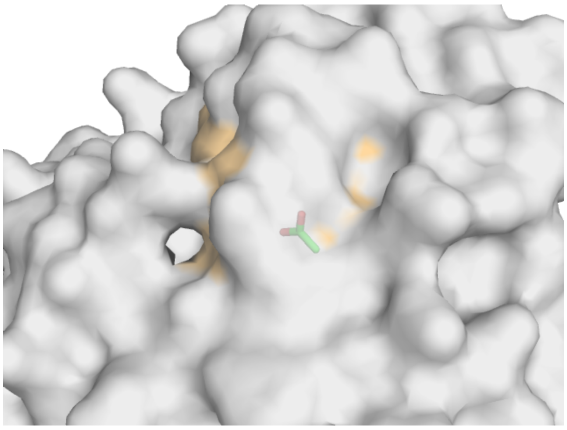

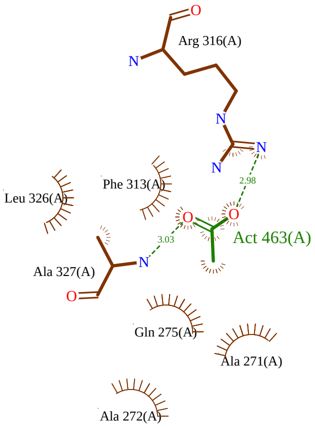

protein	amino acids contacts (binding site)
PDB:3e94:chainA:P19793	A A Q F R L A
PDB:3kwy:chainA:P19793	A A Q F R L A
tr:B3KY83:B3KY83_HUMAN	A A Q F R L A
sp:P19793:RXRA_HUMAN	A A Q F R L A
tr:F1D8Q5:F1D8Q5_HUMAN	A A Q F R L A
tr:Q6P3U7:Q6P3U7_HUMAN	A A Q F R L A
tr:Q0VJ96:Q0VJ96_RAT	A A Q F R L A
tr:Q6LC96:Q6LC96_MOUSE	A A Q F R L A
sp:P28700:RXRA_MOUSE	A A Q F R L A
tr:A2AJP1:A2AJP1_MOUSE	A A Q F R L A
sp:P20153:USP_DROME	V V Q F R L N

protein	whole protein ident	simil	domain-based ident	simil	contact-based ident	simil
PDB:3e94:chainA:P19793	1.0	1.0	1.0	1.0	1.0	1.0
PDB:3kwy:chainA:P19793	1.0	1.0	1.0	1.0	1.0	1.0
tr:B3KY83:B3KY83_HUMAN	0.79	0.79	1.0	1.0	1.0	1.0
sp:P19793:RXRA_HUMAN	1.0	1.0	1.0	1.0	1.0	1.0
tr:F1D8Q5:F1D8Q5_HUMAN	1.0	1.0	1.0	1.0	1.0	1.0
tr:Q6P3U7:Q6P3U7_HUMAN	0.9	0.9	1.0	1.0	1.0	1.0
tr:Q0VJ96:Q0VJ96_RAT	0.97	0.99	1.0	1.0	1.0	1.0
tr:Q6LC96:Q6LC96_MOUSE	0.92	0.93	1.0	1.0	1.0	1.0
sp:P28700:RXRA_MOUSE	0.97	0.99	1.0	1.0	1.0	1.0
tr:A2AJP1:A2AJP1_MOUSE	0.97	0.99	1.0	1.0	1.0	1.0
sp:P20153:USP_DROME	0.37	0.61	0.4	0.66	0.57	0.63

usp (FBgn0003964) associated phenotypes

circadian rhythm defective, germline clone, heat sensitive, hypoactive, lethal - all die before end of pupal stage, lethal - all die during P-stage, lethal - all die during first instar larval stage, maternal effect, neuroanatomy defective, partially, rescuable maternal effect, somatic clone, some die during pupal stage, touch sensitivity defective

(Information from FlyBase)

usp (UniProt:P20153) annotation

Function: Receptor for ecdysone. May be an important modulator of insect metamorphosis. Plays an important part in embryonic and post-embryonic development. Binds to ecdysone response elements (ECRES) such as in the promoter region of s15 chorion gene.
Subunit: Heterodimer of USP and ECR. Only the heterodimer is capable of high-affinity binding to ecdysone. (PubMed:8247157).
Subcellular location: Nucleus.
Domain: Composed of three domains: a modulating N-terminal domain, a DNA-binding domain and a C-terminal ligand-binding domain.

(Information from UniProt)

Filamin-A

Best gene implication in ageing for this target family came from gene Q9VEN1 annotated in UniProt release 2014_02. Annotation GO 8340 (determination of adult lifespan) was Inferred from Mutant Phenotype

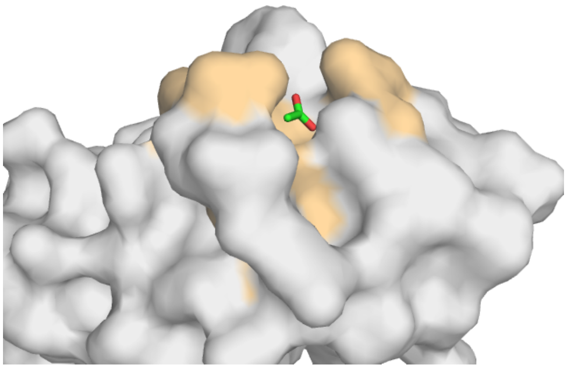

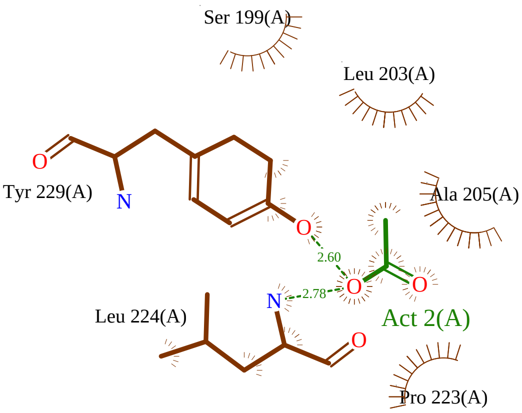

protein	amino acids contacts (binding site)						
PDB:3rgh:chainA:P21333	S	L	P	A	P	L	Y
tr:H0Y5C6:H0Y5C6_HUMAN	-	S	K	A	V	Q	Y
tr:E9PHF0:E9PHF0_HUMAN	-	S	K	A	V	Q	Y
tr:Q96C61:Q96C61_HUMAN	-	S	K	A	V	Q	Y
tr:Q6NXF2:Q6NXF2_HUMAN	-	S	K	A	V	Q	Y
tr:Q59H94:Q59H94_HUMAN	-	S	K	A	V	Q	Y
tr:Q8TES4:Q8TES4_HUMAN	-	S	K	A	V	Q	Y
tr:E7EN95:E7EN95_HUMAN	-	S	K	A	A	Q	Y
sp:O75369:FLNB_HUMAN	-	S	K	A	A	Q	Y
tr:Q5HY54:Q5HY54_HUMAN	-	S	K	A	V	Q	Y
tr:Q60FE6:Q60FE6_HUMAN	-	S	K	A	V	Q	Y
tr:Q60FE5:Q60FE5_HUMAN	-	S	K	A	V	Q	Y
sp:P21333:FLNA_HUMAN	-	S	K	A	V	Q	Y
sp:Q14315:FLNC_HUMAN	-	S	K	A	V	Q	Y
tr:D3ZD13:D3ZD13_RAT	-	S	K	A	A	Q	Y
tr:D4A8D5:D4A8D5_RAT	-	S	K	A	A	Q	Y
tr:C0JPT7:C0JPT7_RAT	-	S	K	A	V	Q	Y
sp:D3ZHA0:FLNC_RAT	-	S	K	A	V	Q	Y
tr:J3JS91:J3JS91_MOUSE	-	S	K	A	V	Q	Y
tr:Q3UXJ8:Q3UXJ8_MOUSE	-	S	K	A	V	Q	Y
tr:Q3U7N9:Q3U7N9_MOUSE	-	C	E	A	P	T	Y
tr:B7FAV1:B7FAV1_MOUSE	-	S	K	A	V	Q	Y
sp:Q80X90:FLNB_MOUSE	-	S	K	A	A	Q	Y
tr:B7FAU9:B7FAU9_MOUSE	-	S	K	A	V	Q	Y
sp:Q8BTM8:FLNA_MOUSE	-	S	K	A	V	Q	Y
tr:D3YW87:D3YW87_MOUSE	-	S	K	A	V	Q	Y
sp:Q8VHX6:FLNC_MOUSE	-	S	K	A	V	Q	Y
tr:D3Z576:D3Z576_MOUSE	-	S	K	A	V	Q	Y
tr:Q6NN54:Q6NN54_DROME	-	S	K	A	V	T	Y
tr:C7LAD0:C7LAD0_DROME	-	S	K	A	V	T	Y
tr:A4V310:A4V310_DROME	-	S	K	A	V	T	Y
tr:C7LAD1:C7LAD1_DROME	-	S	K	A	V	T	Y
tr:B7Z0L2:B7Z0L2_DROME	-	S	K	A	V	T	Y
tr:Q8T3K7:Q8T3K7_DROME	-	S	K	A	V	T	Y
tr:Q29R34:Q29R34_DROME	-	S	K	A	V	T	Y
sp:Q9VEN1:FLNA_DROME	-	S	K	A	V	T	Y
tr:Q9GQV2:Q9GQV2_DROME	-	S	K	A	V	T	Y
tr:Q7KSF4:Q7KSF4_DROME	-	S	K	A	V	T	Y
tr:D0IMZ6:D0IMZ6_CAEEL	-	S	K	A	V	A	Y
tr:H2KZT3:H2KZT3_CAEEL	-	T	R	T	P	K	Y
tr:Q3V5H2:Q3V5H2_CAEEL	-	S	K	A	V	A	Y
tr:Q9TYW4:Q9TYW4_CAEEL	-	T	R	T	P	K	Y
tr:D0IMZ8:D0IMZ8_CAEEL	-	S	K	A	V	A	Y
tr:D0IMZ7:D0IMZ7_CAEEL	-	S	K	A	V	A	Y
tr:D0IMZ5:D0IMZ5_CAEEL	-	S	K	A	V	A	Y

protein	whole protein ident	simil	domain-based ident	simil	contact-based ident	simil
PDB:3rgh:chainA:P21333	0.32	0.73	0.31	0.74	1.0	1.0
tr:H0Y5C6:H0Y5C6_HUMAN	0.11	0.11	1.0	1.0	0.29	0.27
tr:E9PHF0:E9PHF0_HUMAN	0.29	0.29	1.0	1.0	0.29	0.27
tr:Q96C61:Q96C61_HUMAN	0.32	0.32	1.0	1.0	0.29	0.27
tr:Q6NXF2:Q6NXF2_HUMAN	0.37	0.37	1.0	1.0	0.29	0.27
tr:Q59H94:Q59H94_HUMAN	0.33	0.42	0.89	0.96	0.29	0.27
tr:Q8TES4:Q8TES4_HUMAN	0.55	0.55	1.0	1.0	0.29	0.27
tr:E7EN95:E7EN95_HUMAN	0.63	0.82	0.81	0.95	0.29	0.27
sp:O75369:FLNB_HUMAN	0.69	0.89	0.81	0.95	0.29	0.27
tr:Q5HY54:Q5HY54_HUMAN	0.98	0.98	1.0	1.0	0.29	0.27
tr:Q60FE6:Q60FE6_HUMAN	0.99	0.99	1.0	1.0	0.29	0.27
tr:Q60FE5:Q60FE5_HUMAN	0.99	0.99	1.0	1.0	0.29	0.27
sp:P21333:FLNA_HUMAN	1.0	1.0	1.0	1.0	0.29	0.27
sp:Q14315:FLNC_HUMAN	0.7	0.88	0.89	0.96	0.29	0.27
tr:D3ZD13:D3ZD13_RAT	0.3	0.39	0.81	0.95	0.29	0.27
tr:D4A8D5:D4A8D5_RAT	0.68	0.88	0.81	0.95	0.29	0.27
tr:C0JPT7:C0JPT7_RAT	0.97	0.99	0.99	1.0	0.29	0.27
sp:D3ZHA0:FLNC_RAT	0.7	0.89	0.89	0.96	0.29	0.27
tr:J3JS91:J3JS91_MOUSE	0.1	0.1	0.98	0.99	0.29	0.27
tr:Q3UXJ8:Q3UXJ8_MOUSE	0.19	0.23	0.89	0.96	0.29	0.27
tr:Q3U7N9:Q3U7N9_MOUSE	0.38	0.39	0.39	0.66	0.43	0.22
tr:B7FAV1:B7FAV1_MOUSE	0.95	0.97	0.98	0.99	0.29	0.27
sp:Q80X90:FLNB_MOUSE	0.68	0.89	0.81	0.95	0.29	0.27
tr:B7FAU9:B7FAU9_MOUSE	0.97	0.99	0.98	0.99	0.29	0.27
sp:Q8BTM8:FLNA_MOUSE	0.97	0.99	0.98	0.99	0.29	0.27
tr:D3YW87:D3YW87_MOUSE	0.7	0.87	0.89	0.96	0.29	0.27
sp:Q8VHX6:FLNC_MOUSE	0.7	0.89	0.89	0.96	0.29	0.27
tr:D3Z576:D3Z576_MOUSE	0.7	0.88	0.89	0.96	0.29	0.27
tr:Q6NN54:Q6NN54_DROME	0.17	0.27	0.65	0.88	0.29	0.28
tr:C7LAD0:C7LAD0_DROME	0.17	0.27	0.65	0.88	0.29	0.28
tr:A4V310:A4V310_DROME	0.17	0.27	0.65	0.88	0.29	0.28
tr:C7LAD1:C7LAD1_DROME	0.17	0.29	0.65	0.88	0.29	0.28
tr:B7Z0L2:B7Z0L2_DROME	0.17	0.29	0.65	0.88	0.29	0.28
tr:Q8T3K7:Q8T3K7_DROME	0.24	0.43	0.65	0.88	0.29	0.28
tr:Q29R34:Q29R34_DROME	0.24	0.43	0.65	0.88	0.29	0.28
sp:Q9VEN1:FLNA_DROME	0.41	0.68	0.65	0.88	0.29	0.28
tr:Q9GQV2:Q9GQV2_DROME	0.41	0.68	0.65	0.88	0.29	0.28
tr:Q7KSF4:Q7KSF4_DROME	0.43	0.74	0.65	0.88	0.29	0.28
tr:D0IMZ6:D0IMZ6_CAEEL	0.14	0.26	0.54	0.86	0.29	0.28
tr:H2KZT3:H2KZT3_CAEEL	0.11	0.25	0.19	0.48	0.29	0.34
tr:Q3V5H2:Q3V5H2_CAEEL	0.14	0.27	0.54	0.86	0.29	0.28
tr:Q9TYW4:Q9TYW4_CAEEL	0.13	0.3	0.32	0.73	0.29	0.34
tr:D0IMZ8:D0IMZ8_CAEEL	0.26	0.6	0.54	0.86	0.29	0.28
tr:D0IMZ7:D0IMZ7_CAEEL	0.26	0.6	0.54	0.86	0.29	0.28
tr:D0IMZ5:D0IMZ5_CAEEL	0.26	0.61	0.54	0.86	0.29	0.28

cher (FBgn0014141) associated phenotypes

chemical sensitive, heat sensitive, learning defective, locomotor behavior defective, long lived, male fertile, memory defective, neuroanatomy defective, short lived, uncoordinated

(Information from FlyBase)

cher (UniProt:Q9VEN1) annotation

Function: Involved in the germline ring canal formation. May tether actin microfilament within the ovarian ring canal to the cell membrane. Contributes to actin microfilaments organization. (PubMed:10477759, PubMed:10556087).
Subunit: Interacts with Ten-m. (PubMed:21857973).
Subcellular location: Cytoplasm, cytoskeleton. Cell membrane. Note=Localizes to intercellular bridges corresponding to ring canals that connect cytoplasm of nurse cells to the developing oocyte. Localizes to nurse cell plasma membranes. Detected in both the inner and outer rims of the ring canal.
Tissue specificity: Germline-specific in females (at protein level). Expressed in ovary. (PubMed:10477759,

PubMed:10556087).
Developmental stage: Expressed throughout the egg-chamber development (at protein level). Expressed in the embryo. (PubMed:10477759, PubMed:10556087).
Disruption phenotype: Shows defect in ring canal assembly and female sterility. (PubMed:10477759).

(Information from UniProt)

fln-1 (WBGene00022048) associated phenotypes

reduced brood size

(Information from WormBase)

DNA-(apurinic or apyrimidinic site) lyase

Best gene implication in ageing for this target family came from gene P43138 annotated in UniProt release 2014_02. Annotation GO 7568 (aging) was Inferred from Direct Assay

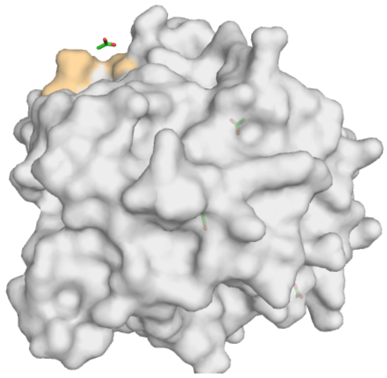

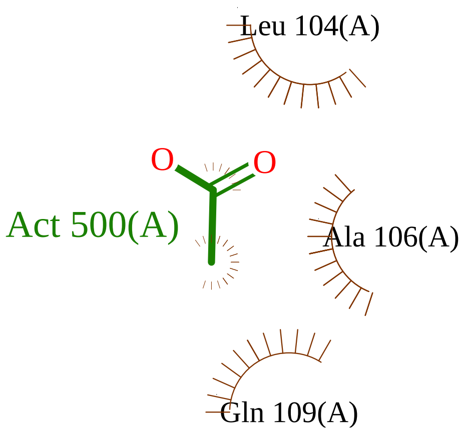

protein	amino acids contacts (binding site)
PDB:2o3h:chainA:P27695	M A E L A Q Y S Y N R S
tr:Q5TZP7:Q5TZP7_HUMAN	A A E L A Q Y S Y N R S
sp:P27695:APEX1_HUMAN	A A E L A Q Y S Y N R S
tr:Q99PF3:Q99PF3_RAT	A A E L A Q Y S Y N R S
sp:P43138:APEX1_RAT	A A E L A Q Y S Y N R S
tr:F6QA74:F6QA74_MOUSE	A A E L A Q Y S Y N R S
tr:Q544Z7:Q544Z7_MOUSE	A A E L A Q Y S Y N R S
sp:P28352:APEX1_MOUSE	A A E L A Q Y S Y N R S
sp:P27864:RRP1_DROME	L D E L E T Y A Y N R A
tr:B7YZZ9:B7YZZ9_DROME	L D E L E T Y A Y N R A
tr:G5EBR7:G5EBR7_CAEEL	- - E W P E Y A Y N R Q
sp:P38207:APN2_YEAST	- - E I S G Y S Y N R P

	whole protein		domain-based		contact-based	
protein	ident	simil	ident	simil	ident	simil
PDB:2o3h:chainA:P27695	0.99	0.99	0.99	0.99	1.0	1.0
tr:Q5TZP7:Q5TZP7_HUMAN	1.0	1.0	1.0	1.0	0.92	0.93
sp:P27695:APEX1_HUMAN	1.0	1.0	1.0	1.0	0.92	0.93
tr:Q99PF3:Q99PF3_RAT	0.86	0.9	0.88	0.91	0.92	0.93
sp:P43138:APEX1_RAT	0.93	0.98	0.95	0.99	0.92	0.93
tr:F6QA74:F6QA74_MOUSE	0.86	0.9	0.89	0.91	0.92	0.93
tr:Q544Z7:Q544Z7_MOUSE	0.94	0.98	0.96	0.99	0.92	0.93
sp:P28352:APEX1_MOUSE	0.94	0.98	0.96	0.99	0.92	0.93
sp:P27864:RRP1_DROME	0.23	0.37	0.49	0.79	0.5	0.62
tr:B7YZZ9:B7YZZ9_DROME	0.22	0.35	0.45	0.72	0.5	0.62
tr:G5EBR7:G5EBR7_CAEEL	0.38	0.7	0.39	0.71	0.42	0.47
sp:P38207:APN2_YEAST	0.14	0.35	0.2	0.51	0.5	0.46

Rrp1 (UniProt:P27864) annotation
Function: Could promote homologous recombination at sites of DNA damage. Has apurinic endonuclease and double-stranded DNA 3'- exonuclease activities and carries out single-stranded DNA renaturation in a Mg(2+)-dependent manner. Activity is more efficient in purine-rich regions of ds-DNA than in pyrimidine-rich regions. (PubMed:1713691, PubMed:8918793).
Cofactor: Mg(2+)Name=Mn(2+); Xref=ChEBI:CHEBI:29035; Evidence=; Note=Probably binds two magnesium or manganese ions per subunit. ;
Subcellular location: Nucleus (PROSITE- ProRule:PRU00764, PubMed:1713691, PubMed:8918793).
(Information from UniProt)

Aldehyde dehydrogenase, dimeric NADP-preferring

Best gene implication in ageing for this target family came from gene P11883 annotated in UniProt release 2014_02. Annotation GO 7568 (aging) was Inferred from Expression Pattern

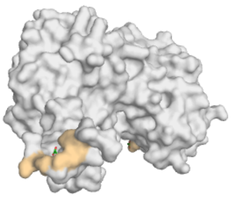

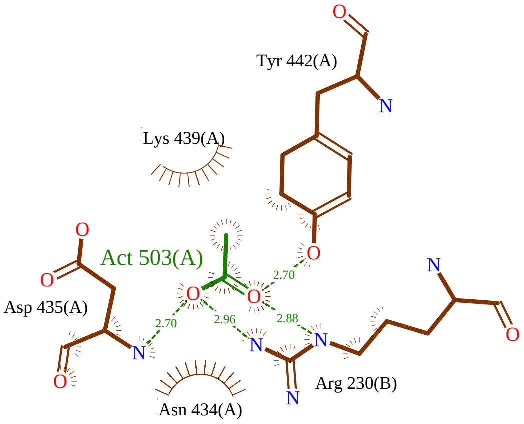

protein	amino acids contacts (binding site)
PDB:3sza:chainA:P30838	R M N D K Y
PDB:3szb:chainA:P30838	R M N D K Y
PDB:4l1o:chainA:P30838	R M N D K Y
PDB:4l2o:chainA:P30838	R M N D K Y
tr:A8MYB8:A8MYB8_HUMAN	R M N D K Y
tr:I3L3I9:I3L3I9_HUMAN	R M N D K Y
sp:P30838:AL3A1_HUMAN	R M N D K Y
sp:P11883:AL3A1_RAT	R L N E K Y
sp:P47739:AL3A1_MOUSE	R R N E K Y
tr:D5SHM9:D5SHM9_DROME	R L G E S Y
tr:Q7JR61:Q7JR61_DROME	R L G E S Y
tr:A1Z6Z3:A1Z6Z3_DROME	R L G E S Y
tr:Q8IGH3:Q8IGH3_DROME	R L G E S Y
tr:E3CTS3:E3CTS3_DROME	R L G E S Y
tr:A4UZ69:A4UZ69_DROME	R L G E S Y
tr:A1Z6Z4:A1Z6Z4_DROME	R L G E S Y
tr:F0JAM9:F0JAM9_DROME	R L G E S Y
tr:Q86S57:Q86S57_CAEEL	R F G E A Y
tr:Q8MXJ7:Q8MXJ7_CAEEL	R F G E A Y
tr:O16518:O16518_CAEEL	R F G E A Y
sp:Q04458:HFD1_YEAST	R W N D F Y

protein	whole protein ident	simil	domain-based ident	simil	contact-based ident	simil
PDB:3sza:chainA:P30838	1.0	1.0	1.0	1.0	1.0	1.0
PDB:3szb:chainA:P30838	1.0	1.0	1.0	1.0	1.0	1.0
PDB:4l1o:chainA:P30838	1.0	1.0	1.0	1.0	1.0	1.0
PDB:4l2o:chainA:P30838	1.0	1.0	1.0	1.0	1.0	1.0
tr:A8MYB8:A8MYB8_HUMAN	0.86	0.86	0.72	0.72	1.0	1.0
tr:I3L3I9:I3L3I9_HUMAN	0.84	0.84	1.0	1.0	1.0	1.0
sp:P30838:AL3A1_HUMAN	1.0	1.0	1.0	1.0	1.0	1.0
sp:P11883:AL3A1_RAT	0.82	0.95	0.86	0.96	0.67	0.91
sp:P47739:AL3A1_MOUSE	0.8	0.94	0.85	0.96	0.67	0.79
tr:D5SHM9:D5SHM9_DROME	0.4	0.66	0.42	0.71	0.33	0.62
tr:Q7JR61:Q7JR61_DROME	0.46	0.76	0.42	0.7	0.33	0.62
tr:A1Z6Z3:A1Z6Z3_DROME	0.46	0.77	0.42	0.71	0.33	0.62
tr:Q8IGH3:Q8IGH3_DROME	0.46	0.77	0.42	0.71	0.33	0.62
tr:E3CTS3:E3CTS3_DROME	0.46	0.76	0.42	0.71	0.33	0.62
tr:A4UZ69:A4UZ69_DROME	0.4	0.67	0.42	0.7	0.33	0.62
tr:A1Z6Z4:A1Z6Z4_DROME	0.4	0.68	0.42	0.71	0.33	0.62
tr:F0JAM9:F0JAM9_DROME	0.4	0.67	0.42	0.71	0.33	0.62
tr:Q86S57:Q86S57_CAEEL	0.45	0.77	0.42	0.71	0.33	0.64
tr:Q8MXJ7:Q8MXJ7_CAEEL	0.45	0.77	0.42	0.71	0.33	0.64
tr:O16518:O16518_CAEEL	0.45	0.77	0.42	0.71	0.33	0.64
sp:Q04458:HFD1_YEAST	0.3	0.67	0.28	0.65	0.67	0.74

Aldh-III (FBgn0010548) associated phenotypes

chemical sensitive, conditional, developmental rate defective, partially lethal, partially lethal - majority die

(Information from FlyBase)

Catenin alpha-1

Best gene implication in ageing for this target family came from gene Q5U302 annotated in UniProt release 2014_02. Annotation GO 7568 (aging) was Inferred from Expression Pattern

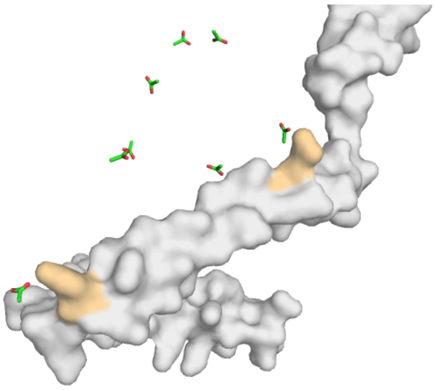

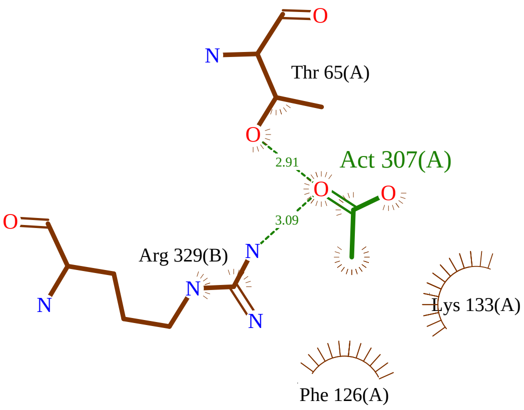

protein	amino acids contacts (binding site)		
PDB:4ehp:chainB:P35221	R	R	Y
tr:H0YBB8:H0YBB8_HUMAN	R	R	Y
tr:B4DKT9:B4DKT9_HUMAN	R	R	Y
tr:F8W845:F8W845_HUMAN	R	R	Y
tr:G3XAM7:G3XAM7_HUMAN	R	R	Y
tr:B4E2G8:B4E2G8_HUMAN	R	R	Y
sp:P35221:CTNA1_HUMAN	R	R	Y
tr:Q5U302:Q5U302_RAT	R	R	Y
tr:Q545R0:Q545R0_MOUSE	R	R	Y
sp:P26231:CTNA1_MOUSE	R	R	Y
sp:P35220:CTNA_DROME	R	R	Y
tr:I1WYI4:I1WYI4_DROME	R	R	Y
tr:I7J4D2:I7J4D2_CAEEL	R	K	Y
tr:I7LFF2:I7LFF2_CAEEL	R	K	Y
tr:I7K4K5:I7K4K5_CAEEL	R	K	Y
sp:P90947:HMP1_CAEEL	R	K	Y
tr:A5JYX3:A5JYX3_CAEEL	R	K	Y

	whole protein		domain-based		contact-based	
protein	ident	simil	ident	simil	ident	simil
PDB:4ehp:chainB:P35221	1.0	1.0	1.0	1.0	1.0	1.0
tr:H0YBB8:H0YBB8_HUMAN	0.12	0.13	0.26	0.31	1.0	1.0
tr:B4DKT9:B4DKT9_HUMAN	0.86	0.86	1.0	1.0	1.0	1.0
tr:F8W845:F8W845_HUMAN	0.89	0.89	1.0	1.0	1.0	1.0
tr:G3XAM7:G3XAM7_HUMAN	0.9	0.92	1.0	1.0	1.0	1.0
tr:B4E2G8:B4E2G8_HUMAN	0.98	0.98	1.0	1.0	1.0	1.0
sp:P35221:CTNA1_HUMAN	1.0	1.0	1.0	1.0	1.0	1.0
tr:Q5U302:Q5U302_RAT	0.99	1.0	1.0	1.0	1.0	1.0
tr:Q545R0:Q545R0_MOUSE	0.99	1.0	1.0	1.0	1.0	1.0
sp:P26231:CTNA1_MOUSE	0.99	1.0	1.0	1.0	1.0	1.0
sp:P35220:CTNA_DROME	0.61	0.86	0.71	0.92	1.0	1.0
tr:I1WYI4:I1WYI4_DROME	0.6	0.85	0.71	0.92	1.0	1.0
tr:I7J4D2:I7J4D2_CAEEL	0.35	0.7	0.48	0.84	0.67	0.88
tr:I7LFF2:I7LFF2_CAEEL	0.36	0.72	0.48	0.84	0.67	0.88
tr:I7K4K5:I7K4K5_CAEEL	0.36	0.72	0.48	0.84	0.67	0.88
sp:P90947:HMP1_CAEEL	0.36	0.73	0.48	0.84	0.67	0.88
tr:A5JYX3:A5JYX3_CAEEL	0.36	0.73	0.48	0.84	0.67	0.88

alpha-Cat (FBgn0010215) associated phenotypes

cell lethal, cell migration defective, heat sensitive, increased cell number, lethal - all die before end of pupal stage, lethal - all die during embryonic stage, lethal - all die during pupal stage, neuroanatomy defective, partially, somatic clone, some die during pupal stage

(Information from FlyBase)

alpha-Cat (UniProt:P35220) annotation

Function: Associates with the cytoplasmic domain of a variety of cadherins. The association of catenins to cadherins produces a complex which is linked to the actin filament network, and which seems to be of primary importance for cadherins cell-adhesion properties. Can associate with the armadillo protein.

Subcellular location: Cytoplasm, cytoskeleton Cell junction, adherens junction Cell membrane ; Peripheral membrane protein ; Cytoplasmic side Cell junction. Note=Found only at cell-cell boundaries.

Developmental stage: Present at all stages, but reached the highest levels during early to mid-embryogenesis.

(Information from UniProt)

hmp-1 (WBGene00001978) associated phenotypes

body morphology variant, embryonic lethal, lethal, protein subcellular localization variant, sterile

(Information from WormBase)

hmp-1 (UniProt:P90947) annotation

Function: Required for cell migration during body enclosure and cell shape changes during body elongation. (PubMed:9531567).
Subunit: May interact with hmp-2.
Subcellular location: Cell junction, adherens junction (PubMed:9531567).
Tissue specificity: Epidermal cells. (PubMed:9531567).
Developmental stage: Present in all embryonic blastomeres at early stages of development (at protein level). (PubMed:9531567).
Disruption phenotype: Worms arrest their elongation at the 1.25X to 1.5X stage. (PubMed:9531567).

(Information from UniProt)

Cystathionine beta-synthase

Best gene implication in ageing for this target family came from gene Q9VRD9 annotated in UniProt release 2014_02. Annotation GO 8340 (determination of adult lifespan) was Inferred from Mutant Phenotype

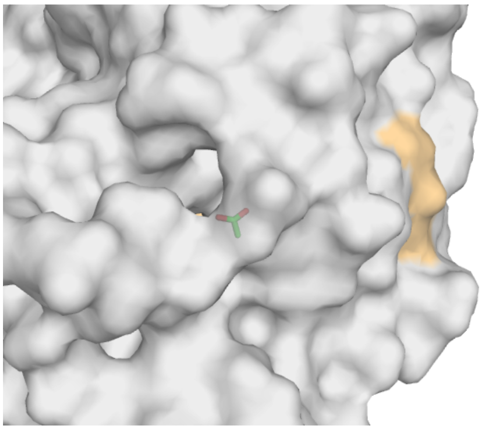

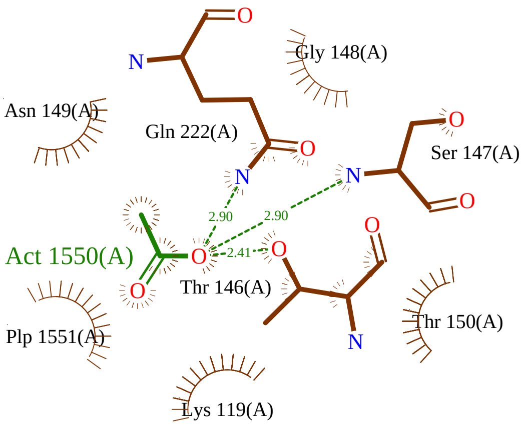

protein	amino acids contacts (binding site)
PDB:4coo:chainA:P35520	K T S G N T I V Q
tr:F5H2U1:F5H2U1_HUMAN	K T S G N T I V Q
sp:P35520:CBS_HUMAN	K T S G N T I V Q
sp:P32232:CBS_RAT	K T S G N T I V Q
sp:Q91WT9:CBS_MOUSE	K T S G N T I V Q
tr:Q9VRD9:Q9VRD9_DROME	K T S G N T I I Q
tr:Q9N4K2:Q9N4K2_CAEEL	K T S G N T I V Q
sp:P32582:CBS_YEAST	K T S G N T I I Q
tr:E9P980:E9P980_YEASX	K T S G N T I I Q

	whole protein		domain-based		contact-based	
protein	ident	simil	ident	simil	ident	simil
PDB:4coo:chainA:P35520	1.0	1.0	1.0	1.0	1.0	1.0
tr:F5H2U1:F5H2U1_HUMAN	0.84	0.84	0.97	0.97	1.0	1.0
sp:P35520:CBS_HUMAN	1.0	1.0	1.0	1.0	1.0	1.0
sp:P32232:CBS_RAT	0.82	0.92	0.91	0.97	1.0	1.0
sp:Q91WT9:CBS_MOUSE	0.82	0.92	0.93	0.98	1.0	1.0
tr:Q9VRD9:Q9VRD9_DROME	0.47	0.77	0.65	0.89	0.89	0.97
tr:Q9N4K2:Q9N4K2_CAEEL	0.24	0.39	0.56	0.84	1.0	1.0
sp:P32582:CBS_YEAST	0.36	0.64	0.54	0.83	0.89	0.97
tr:E9P980:E9P980_YEASX	0.35	0.64	0.53	0.83	0.89	0.97

Cbs (FBgn0031148) associated phenotypes

RU486 conditional, long lived, short lived

(Information from FlyBase)

Cbs (UniProt:Q9VRD9) annotation
Cofactor: pyridoxal 5'-phosphate Evidence=(RuleBase:RU361204);

(Information from UniProt)

Phosphoenolpyruvate carboxykinase, cytosolic [GTP]

Best gene implication in ageing for this target family came from gene Q9Z2V4 via mapping the annotation from Ensembl ENSMUSG00000027513 via mapping the annotation from EntrezGene 18534 via mapping the annotation from GenAgeModels 0497 annotated in GenAge release 17.

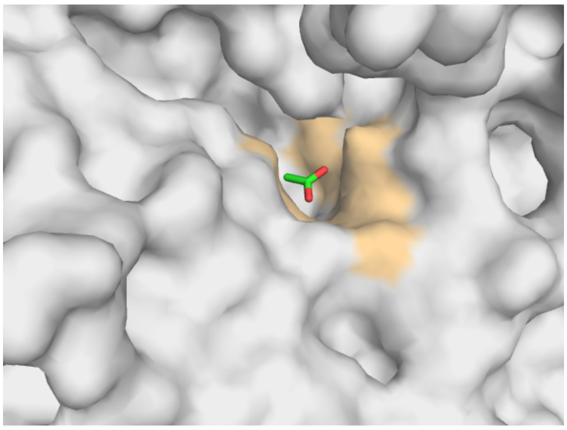

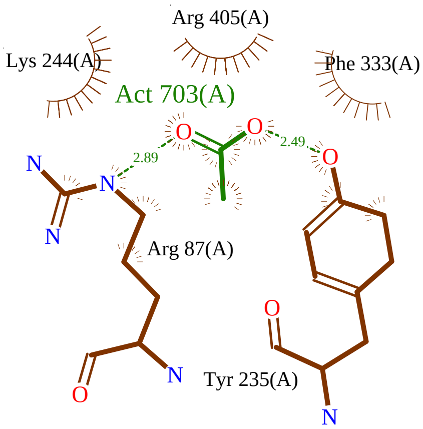

protein	amino acids contacts (binding site)
PDB:1khb:chainA:P35558	R Y K F R
PDB:1m51:chainA:P35558	R Y K F R
sp:P35558:PCKGC_HUMAN	R Y K F R
sp:P07379:PCKGC_RAT	R Y K F R
sp:Q9Z2V4:PCKGC_MOUSE	R Y K F R
tr:A8DYI3:A8DYI3_DROME	R Y K F R
tr:Q7JXB5:Q7JXB5_DROME	R Y K F R
sp:P20007:PCKG_DROME	R Y K F R
tr:Q7JKI1:Q7JKI1_CAEEL	R Y K F R
tr:Q7JKI3:Q7JKI3_CAEEL	R Y K F R
tr:Q8ITY0:Q8ITY0_CAEEL	R Y K F R
tr:Q7JKI2:Q7JKI2_CAEEL	R Y K F R
tr:O02286:O02286_CAEEL	R Y K F R
tr:O44906:O44906_CAEEL	R Y K F R

	whole protein		domain-based		contact-based	
protein	ident	simil	ident	simil	ident	simil
PDB:1khb:chainA:P35558	0.99	1.0	0.99	1.0	1.0	1.0
PDB:1m51:chainA:P35558	0.99	1.0	0.99	1.0	1.0	1.0
sp:P35558:PCKGC_HUMAN	1.0	1.0	1.0	1.0	1.0	1.0
sp:P07379:PCKGC_RAT	0.91	0.98	0.91	0.98	1.0	1.0
sp:Q9Z2V4:PCKGC_MOUSE	0.91	0.98	0.91	0.98	1.0	1.0
tr:A8DYI3:A8DYI3_DROME	0.6	0.8	0.61	0.82	1.0	1.0
tr:Q7JXB5:Q7JXB5_DROME	0.62	0.85	0.65	0.88	1.0	1.0
sp:P20007:PCKG_DROME	0.61	0.84	0.65	0.88	1.0	1.0
tr:Q7JKI1:Q7JKI1_CAEEL	0.47	0.72	0.51	0.75	1.0	1.0
tr:Q7JKI3:Q7JKI3_CAEEL	0.55	0.81	0.57	0.84	1.0	1.0
tr:Q8ITY0:Q8ITY0_CAEEL	0.5	0.73	0.53	0.77	1.0	1.0
tr:Q7JKI2:Q7JKI2_CAEEL	0.56	0.84	0.58	0.86	1.0	1.0
tr:O02286:O02286_CAEEL	0.54	0.82	0.58	0.86	1.0	1.0
tr:O44906:O44906_CAEEL	0.53	0.8	0.57	0.85	1.0	1.0

Pepck (UniProt:P20007) annotation
Function: Catalyzes the conversion of oxaloacetate (OAA) to phosphoenolpyruvate (PEP), the rate-limiting step in the metabolic pathway that produces glucose from lactate and other precursors derived from the citric acid cycle.
Cofactor: Mn(2+)Note=Binds 1 Mn(2+) ion per subunit. ;
Pathway: Carbohydrate biosynthesis; gluconeogenesis.
Subunit: Monomer.

(Information from UniProt)

Peptide methionine sulfoxide reductase

Best gene implication in ageing for this target family came from gene P08761 annotated in UniProt release 2014_02. Annotation GO 8340 (determination of adult lifespan) was Inferred from Mutant Phenotype

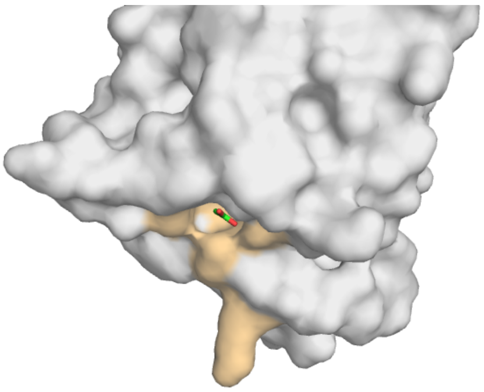

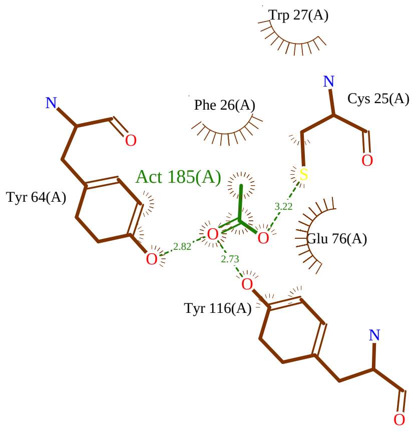

protein	amino acids contacts (binding site)						
PDB:3pil:chainA:P40029	C	F	W	Y	K	E	Y
tr:E5RIA9:E5RIA9_HUMAN	C	F	W	Y	K	E	Y
sp:Q9UJ68:MSRA_HUMAN	C	F	W	Y	K	E	Y
tr:M0RCJ2:M0RCJ2_RAT	C	F	W	Y	K	E	Y
tr:F1LSJ6:F1LSJ6_RAT	C	F	W	Y	K	E	Y
tr:M0RCC8:M0RCC8_RAT	C	F	W	Y	K	E	Y
sp:Q923M1:MSRA_RAT	C	F	W	Y	K	E	Y
sp:Q9D6Y7:MSRA_MOUSE	C	F	W	Y	K	E	Y
tr:Q7KUM8:Q7KUM8_DROME	C	F	W	Y	R	E	Y
sp:P08761:MSRA_DROME	C	F	W	Y	R	E	Y
tr:B5X0L2:B5X0L2_DROME	C	F	W	Y	R	E	Y
tr:Q6NLA4:Q6NLA4_DROME	C	F	W	Y	R	E	Y
tr:O02089:O02089_CAEEL	C	F	W	Y	K	E	Y
sp:P40029:MSRA_YEAST	C	F	W	Y	K	E	Y

protein	whole protein ident	simil	domain-based ident	simil	contact-based ident	simil
PDB:3pil:chainA:P40029	0.99	1.0	1.0	1.0	1.0	1.0
tr:E5RIA9:E5RIA9_HUMAN	0.2	0.49	0.25	0.62	1.0	1.0
sp:Q9UJ68:MSRA_HUMAN	0.26	0.55	0.34	0.72	1.0	1.0
tr:M0RCJ2:M0RCJ2_RAT	0.23	0.5	0.26	0.55	1.0	1.0
tr:F1LSJ6:F1LSJ6_RAT	0.21	0.44	0.26	0.55	1.0	1.0
tr:M0RCC8:M0RCC8_RAT	0.18	0.39	0.26	0.55	1.0	1.0
sp:Q923M1:MSRA_RAT	0.27	0.55	0.35	0.71	1.0	1.0
sp:Q9D6Y7:MSRA_MOUSE	0.27	0.55	0.34	0.71	1.0	1.0
tr:Q7KUM8:Q7KUM8_DROME	0.18	0.43	0.27	0.63	0.86	0.96
sp:P08761:MSRA_DROME	0.19	0.44	0.27	0.63	0.86	0.96
tr:B5X0L2:B5X0L2_DROME	0.19	0.44	0.27	0.63	0.86	0.96
tr:Q6NLA4:Q6NLA4_DROME	0.19	0.44	0.27	0.64	0.86	0.96
tr:O02089:O02089_CAEEL	0.2	0.47	0.26	0.63	1.0	1.0
sp:P40029:MSRA_YEAST	1.0	1.0	1.0	1.0	1.0	1.0

Eip71CD (FBgn0000565) associated phenotypes

RU486 conditional, increased cell number, long lived, oxidative stress response defective, size defective

(Information from FlyBase)

Eip71CD (UniProt:P08761) annotation

Function: Has an important function as a repair enzyme for proteins that have been inactivated by oxidation. Catalyzes the reversible oxidation-reduction of methionine sulfoxide in proteins to methionine. (PubMed:12145281).
Induction: By ecdysone.

(Information from UniProt)

Bloom syndrome protein

Best gene implication in ageing for this target family came from gene O18017 annotated in UniProt release 2014_02. Annotation GO 8340 (determination of adult lifespan) was Inferred from Mutant Phenotype

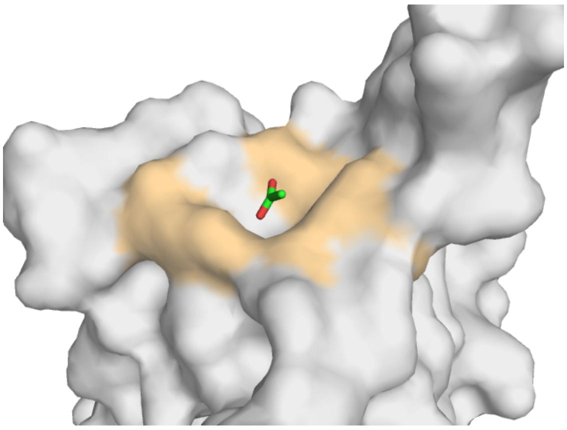

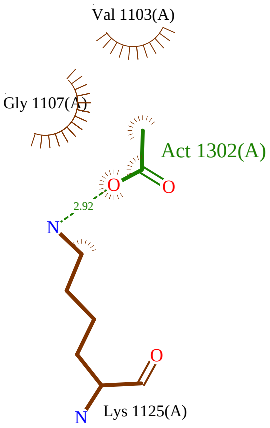

protein	amino acids contacts (binding site)
PDB:3we2:chainA:P54132	H V G K
PDB:3we3:chainA:P54132	H V G K
sp:P54132:BLM_HUMAN	H V G K
tr:D3ZQW1:D3ZQW1_RAT	H - G K
tr:E9PZ97:E9PZ97_MOUSE	H - G K
sp:O88700:BLM_MOUSE	H - G K
sp:Q9VGI8:BLM_DROME	L - S H
sp:O18017:BLM_CAEEL	M - - H
sp:P35187:SGS1_YEAST	I - - H

	whole protein		domain-based		contact-based	
protein	ident	simil	ident	simil	ident	simil
PDB:3we2:chainA:P54132	1.0	1.0	1.0	1.0	1.0	1.0
PDB:3we3:chainA:P54132	1.0	1.0	1.0	1.0	1.0	1.0
sp:P54132:BLM_HUMAN	1.0	1.0	1.0	1.0	1.0	1.0
tr:D3ZQW1:D3ZQW1_RAT	0.75	0.91	0.91	0.96	0.75	0.5
tr:E9PZ97:E9PZ97_MOUSE	0.76	0.91	0.87	0.96	0.75	0.5
sp:O88700:BLM_MOUSE	0.76	0.91	0.87	0.96	0.75	0.5
sp:Q9VGI8:BLM_DROME	0.22	0.54	0.29	0.69	0.0	0.27
sp:O18017:BLM_CAEEL	0.2	0.45	0.23	0.63	0.0	0.0
sp:P35187:SGS1_YEAST	0.21	0.56	0.27	0.67	0.0	0.0

Blm (FBgn0002906) associated phenotypes

DNA repair defective, chemical sensitive, dominant, increased cell death, maternal effect, mitotic cell cycle defective, partially, radiation sensitive

(Information from FlyBase)

Blm (UniProt:Q9VGI8) annotation

Function: Participates in DNA replication and repair. Exhibits a magnesium-dependent ATP-dependent DNA-helicase activity that unwinds single- and double-stranded DNA in a 3'-5' direction (By similarity).
Subcellular location: Nucleus

(Information from UniProt)

him-6 (WBGene00001865) associated phenotypes

XXX progeny, autosomal nondisjunction meiosis, embryonic lethal, germline mortal, high incidence male progeny, male fertility reduced, male sterile, nullo X gamete, reduced brood size

(Information from WormBase)

him-6 (UniProt:O18017) annotation

Function: Participates in DNA replication and repair. Exhibits a magnesium-dependent ATP-dependent DNA-helicase activity that unwinds single- and double-stranded DNA in a 3'-5' direction (By similarity).
Subcellular location: Nucleus

(Information from UniProt)

Growth factor receptor-bound protein 2

Best gene implication in ageing for this target family came from gene P62994 annotated in UniProt release 2014_02. Annotation GO 7568 (aging) was Inferred from Physical Interaction

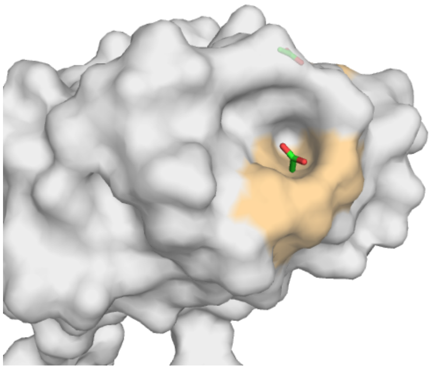

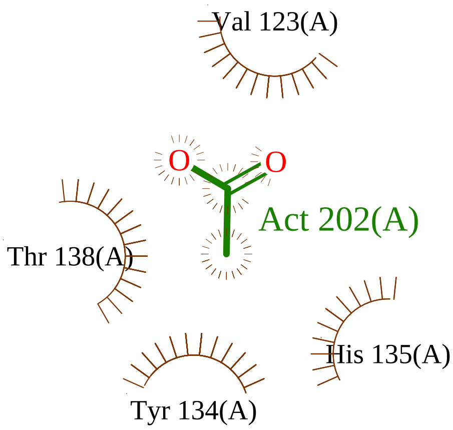

protein	amino acids contacts (binding site)						
PDB:4p9z:chainA:P62993	D	L	R	V	Y	H	T
sp:P62993:GRB2_HUMAN	D	L	R	V	Y	H	T
tr:B0LPF3:B0LPF3_HUMAN	D	L	R	V	Y	H	T
sp:P62994:GRB2_RAT	D	L	R	V	Y	H	T
tr:B1AT92:B1AT92_MOUSE	D	L	R	V	Y	H	T
sp:Q60631:GRB2_MOUSE	D	L	R	V	Y	H	T
tr:Q3U5I5:Q3U5I5_MOUSE	D	L	R	V	Y	H	T
sp:Q08012:DRK_DROME	D	L	R	V	Y	H	A
sp:P29355:SEM5_CAEEL	E	L	R	V	Y	H	A

	whole protein		domain-based		contact-based	
protein	ident	simil	ident	simil	ident	simil
PDB:4p9z:chainA:P62993	1.0	1.0	1.0	1.0	1.0	1.0
sp:P62993:GRB2_HUMAN	1.0	1.0	1.0	1.0	1.0	1.0
tr:B0LPF3:B0LPF3_HUMAN	1.0	1.0	1.0	1.0	1.0	1.0
sp:P62994:GRB2_RAT	1.0	1.0	1.0	1.0	1.0	1.0
tr:B1AT92:B1AT92_MOUSE	0.93	0.94	0.97	0.98	1.0	1.0
sp:Q60631:GRB2_MOUSE	1.0	1.0	0.99	1.0	1.0	1.0
tr:Q3U5I5:Q3U5I5_MOUSE	1.0	1.0	0.99	1.0	1.0	1.0
sp:Q08012:DRK_DROME	0.64	0.86	0.64	0.86	0.86	0.91
sp:P29355:SEM5_CAEEL	0.55	0.81	0.56	0.84	0.71	0.84

drk (FBgn0004638) associated phenotypes

dominant, endocytosis defective, fully, learning defective, lethal - all die before end of pupal stage, memory defective, neuroanatomy defective, rescuable maternal effect, some die during pupal stage

(Information from FlyBase)

drk (UniProt:Q08012) annotation

Function: Required for proper signaling by sevenless. May act to stimulate the ability of Sos to catalyze Ras1 activation by linking sevenless and Sos in a signaling complex. (PubMed:8462097, PubMed:8462098).

Subunit: Interacts with autophosphorylated sev via SH2 domain and Sos, Dos and Dab via SH3 domains. Binds to tyrosine phosphorylated Dab via the SH2 domain. (PubMed:12128212, PubMed:8462097, PubMed:8462098, PubMed:9671493).

Subcellular location: Membrane (PubMed:8462098); Peripheral membrane protein (PubMed:8462098).

Tissue specificity: Found mainly in the developing eye and in the antennal disk. Also observed in other imaginal disks tested and in the embryo.

(Information from UniProt)

sem-5 (WBGene00004774) associated phenotypes

cell migration variant, egg laying variant, hermaphrodite sex muscle development variant, maternal effect lethal emb, rod like larval lethal, sex muscle morphology variant, vulval cell induction variant, vulvaless

(Information from WormBase)

sem-5 (UniProt:P29355) annotation

Function: Acts both in vulval induction and sex myoblast migration. Presumably interacts with the kinase receptor let-23 and with a target that modifies the Ras-like protein let-60.

(Information from UniProt)

Cyclin-dependent kinase 6

Best gene implication in ageing for this target family came from gene F1MA87 via mapping the annotation from RGD 621121 annotated in RGD 2014-03-11. Annotation GO subterm of 7568 (aging) was Inferred from Sequence Orthology

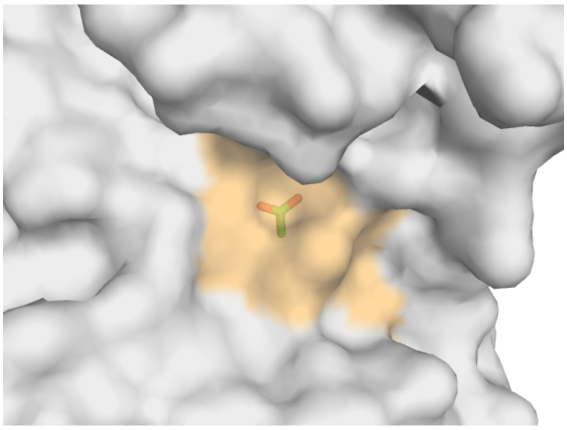

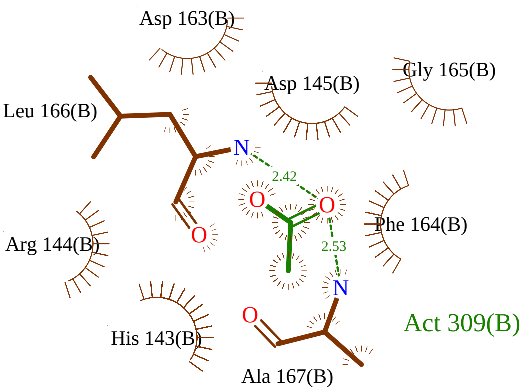

protein	amino acids contacts (binding site)
PDB:2euf:chainB:Q00534	H R D D F G L A
sp:Q00534:CDK6_HUMAN	H R D D F G L A
tr:F1MA87:F1MA87_RAT	H R D D F G L A
sp:Q64261:CDK6_MOUSE	H R D D F G L A
tr:Q0VBK8:Q0VBK8_MOUSE	H R D D F G L A
tr:Q94877:Q94877_DROME	H R D D F G L A
tr:Q7K306:Q7K306_DROME	H R D D F G L A
tr:G5EFI3:G5EFI3_CAEEL	H R D D F G L S
tr:Q9XTR1:Q9XTR1_CAEEL	H R D D F G L S
sp:P00546:CDK1_YEAST	H R D D F G L A

	whole protein		domain-based		contact-based	
protein	ident	simil	ident	simil	ident	simil
PDB:2euf:chainB:Q00534	1.0	1.0	1.0	1.0	1.0	1.0
sp:Q00534:CDK6_HUMAN	1.0	1.0	1.0	1.0	1.0	1.0
tr:F1MA87:F1MA87_RAT	0.97	0.99	0.98	0.99	1.0	1.0
sp:Q64261:CDK6_MOUSE	0.96	0.99	0.98	0.99	1.0	1.0
tr:Q0VBK8:Q0VBK8_MOUSE	0.96	0.99	0.98	0.99	1.0	1.0
tr:Q94877:Q94877_DROME	0.4	0.74	0.44	0.82	1.0	1.0
tr:Q7K306:Q7K306_DROME	0.4	0.74	0.44	0.82	1.0	1.0
tr:G5EFI3:G5EFI3_CAEEL	0.33	0.7	0.4	0.8	0.88	0.88
tr:Q9XTR1:Q9XTR1_CAEEL	0.28	0.61	0.4	0.8	0.88	0.88
sp:P00546:CDK1_YEAST	0.41	0.72	0.44	0.76	1.0	1.0

Cdk4 (FBgn0016131) associated phenotypes

decreased cell number, increased cell size, maternal effect, poor, radiation sensitive, size defective, small body, somatic clone, sterile

(Information from FlyBase)

cdk-4 (WBGene00000406) associated phenotypes
lethal, sterile

(Information from WormBase)

cdk-4 (UniProt:G5EFI3) annotation
Function: Serine/threonine-protein kinase which, in association with cyclin D-like protein cyd-1, is required for the progression through the G1 phase of the cell cycle during postembryonic development by phosphorylating and inhibiting lin-35 and fzr-1 (PubMed:10518501, PubMed:11684669, PubMed:25562820). In complex with cyd-1, involved in sex determination during gonadogenesis by regulating the asymmetric division of the somatic gonadal precursor cell (SGP) (PubMed:16198291). (PubMed:10518501, PubMed:11684669, PubMed:16198291, PubMed:25562820).
Cofactor: Mg(2+) Evidence=(UniProtKB:P24941);
Subunit: Interacts with cyd-1; the interaction is likely involved in regulating cdk-4 activity. (PubMed:10518501).
Developmental stage: Expression initiates during mid-embryogenesis primarily in post-proliferative hypodermal cells and neurons in the head, ventral cord and tail, then declines until hatching where it is mainly seen in seam cells. Expressed throughout larval development in several blast cell linages. In the P lineage, expression is restricted to proliferating cells, whereas it persists in somatic gonads and seam cells. Expressed in uterus and intestine and to a lesser extent in spermatheca. (PubMed:10518501).
Disruption phenotype: RNAi-mediated knockdown results in L4 stage arrest which is associated with uncoordinated movements and a protruding vulva. Impaired cell division of P blast cells, somatic gonad precursors Z1 and Z4 and intestinal cells. Severe defect in the proliferation of P blast cells, intestinal cells, vulva cell precursors and somatic gonad precursors. Mesoblast M cell division is normal. (PubMed:10518501).

(Information from UniProt)

cdk-4 (UniProt:Q9XTR1) annotation
Function: Serine/threonine-protein kinase which, in association with cyclin D-like protein cyd-1, is required for the progression through the G1 phase of the cell cycle during postembryonic development by phosphorylating and inhibiting lin-35 and fzr-1 (PubMed:10518501, PubMed:11684669, PubMed:25562820). In complex with cyd-1, involved in sex determination during gonadogenesis by regulating the asymmetric division of the somatic gonadal precursor cell (SGP) (PubMed:16198291). (PubMed:10518501, PubMed:11684669, PubMed:16198291, PubMed:25562820).
Cofactor: Mg(2+) Evidence=(UniProtKB:P24941);
Subunit: Interacts with cyd-1; the interaction is likely involved in regulating cdk-4 activity. (PubMed:10518501).
Developmental stage: Expression initiates during mid-embryogenesis primarily in post-proliferative hypodermal cells and neurons in the head, ventral cord and tail, then declines until hatching where it is mainly seen in seam cells. Expressed throughout larval development in several blast cell linages. In the P lineage, expression is restricted to proliferating cells, whereas it persists in somatic gonads and seam cells. Expressed in uterus and intestine and to a lesser extent in spermatheca. (PubMed:10518501).
Disruption phenotype: RNAi-mediated knockdown results in L4 stage arrest which is associated with uncoordinated movements and a protruding vulva. Impaired cell division of P blast cells, somatic gonad precursors Z1 and Z4 and intestinal cells. Severe defect in the proliferation of P blast cells, intestinal cells, vulva cell precursors and somatic gonad precursors. Mesoblast M cell division is normal. (PubMed:10518501).

(Information from UniProt)

DNA-binding protein inhibitor ID-2

Best gene implication in ageing for this target family came from gene P41136 annotated in UniProt release 2014_02. Annotation GO subterm of 7568 (aging) was Inferred from Mutant Phenotype

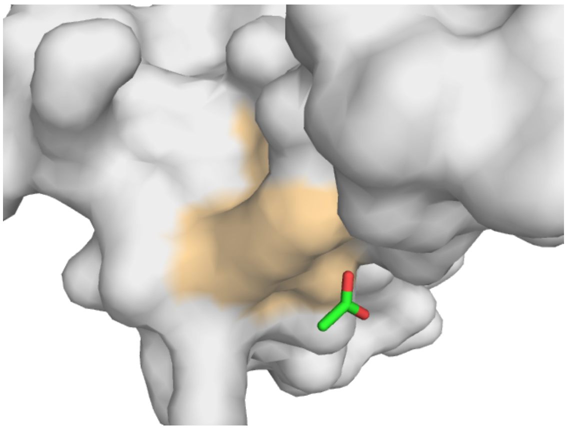

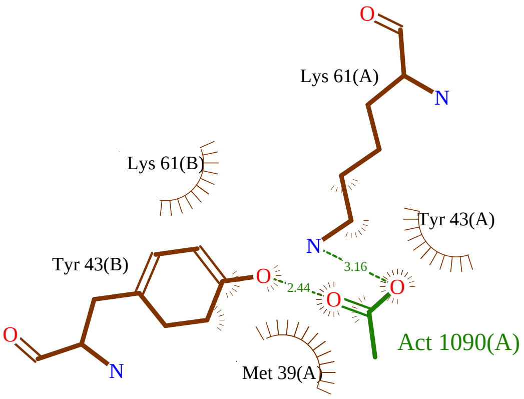

protein	amino acids contacts (binding site)
PDB:4aya:chainA:Q02363	M Y K
PDB:4aya:chainB:Q02363	M Y K
sp:Q02363:ID2_HUMAN	M Y K
tr:Q53T66:Q53T66_HUMAN	M Y K
sp:P41137:ID2_RAT	M Y K
tr:Q545T4:Q545T4_MOUSE	M Y K
sp:P41136:ID2_MOUSE	M Y K
sp:P18491:EMC_DROME	M L K

	whole protein		domain-based		contact-based	
protein	ident	simil	ident	simil	ident	simil
PDB:4aya:chainA:Q02363	0.93	0.98	1.0	1.0	1.0	1.0
PDB:4aya:chainB:Q02363	1.0	1.0	1.0	1.0	1.0	1.0
sp:Q02363:ID2_HUMAN	1.0	1.0	1.0	1.0	1.0	1.0
tr:Q53T66:Q53T66_HUMAN	1.0	1.0	1.0	1.0	1.0	1.0
sp:P41137:ID2_RAT	0.98	0.99	0.98	0.99	1.0	1.0
tr:Q545T4:Q545T4_MOUSE	0.99	1.0	0.98	0.99	1.0	1.0
sp:P41136:ID2_MOUSE	0.99	1.0	0.98	0.99	1.0	1.0
sp:P18491:EMC_DROME	0.22	0.49	0.57	0.85	0.67	0.83

emc (FBgn0000575) associated phenotypes

cell lethal, decreased cell number, dominant, partially lethal - majority die, somatic clone, some die during pupal stage, stress response defective

(Information from FlyBase)

emc (UniProt:P18491) annotation

Function: Participates in sensory organ patterning by antagonizing the neurogenic activity of the Achaete-scute complex (AS-C). It lacks a basic DNA-binding domain but is able to form heterodimers with other HLH proteins, thereby inhibiting DNA binding. May sequester proneural proteins in complexes inefficient for DNA interaction. EMC also affects vein differentiation. Inhibits the activity of AS-C proteins by forming an non-DNA binding heterodimer.
Subunit: Heterodimer with other HLH proteins.
Subcellular location: Nucleus.

(Information from UniProt)

Dual specificity mitogen-activated protein kinase kinase 1

Best gene implication in ageing for this target family came from gene Q02750 annotated in UniProt release 2014_02. Annotation GO subterm of 7568 (aging) was Inferred from Mutant Phenotype

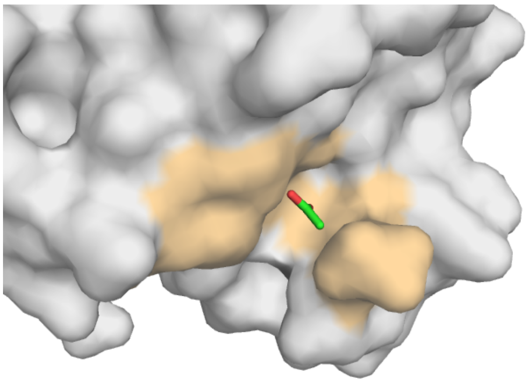

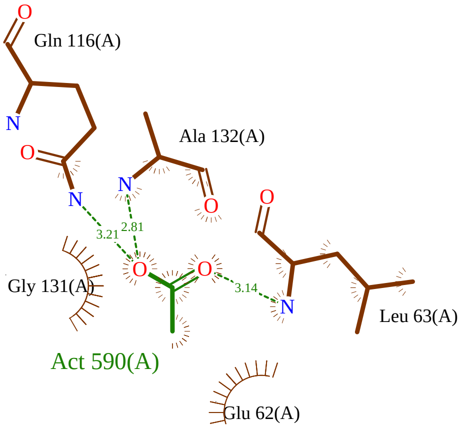

protein	amino acids contacts (binding site)
PDB:3pp1:chainA:Q02750	E L Q H G A
sp:Q02750:MP2K1_HUMAN	E L Q H G A
tr:A4QPA9:A4QPA9_HUMAN	E L Q H G A
sp:Q01986:MP2K1_RAT	E L Q H G A
tr:Q3TMJ8:Q3TMJ8_MOUSE	E L Q H G A
sp:P31938:MP2K1_MOUSE	E L Q H G A
sp:Q24324:DSOR1_DROME	E L K H G A
sp:Q10664:MEK2_CAEEL	E L A H G A
sp:P08018:PBS2_YEAST	R I E H G A

	whole protein		domain-based		contact-based	
protein	ident	simil	ident	simil	ident	simil
PDB:3pp1:chainA:Q02750	1.0	1.0	1.0	1.0	1.0	1.0
sp:Q02750:MP2K1_HUMAN	1.0	1.0	1.0	1.0	1.0	1.0
tr:A4QPA9:A4QPA9_HUMAN	1.0	1.0	1.0	1.0	1.0	1.0
sp:Q01986:MP2K1_RAT	0.99	1.0	1.0	1.0	1.0	1.0
tr:Q3TMJ8:Q3TMJ8_MOUSE	0.99	1.0	1.0	1.0	1.0	1.0
sp:P31938:MP2K1_MOUSE	0.99	1.0	1.0	1.0	1.0	1.0
sp:Q24324:DSOR1_DROME	0.6	0.82	0.75	0.93	0.83	0.92
sp:Q10664:MEK2_CAEEL	0.48	0.74	0.62	0.88	0.83	0.86
sp:P08018:PBS2_YEAST	0.2	0.36	0.45	0.74	0.5	0.86

Dsor1 (FBgn0010269) associated phenotypes
dominant, lethal - all die before end of pupal stage, neuroanatomy defective, polyphasic, some die during immature adult stage, some die during pupal stage

(Information from FlyBase)

Dsor1 (UniProt:Q24324) annotation
Function: Required downstream of Raf in the sevenless (sev), torso (tor), and Drosophila EGF receptor homolog (DER) signal transduction pathways. Involved in both positive regulation (at the posterior terminus) and negative regulation (at the anterior domain) of tll, as in other terminal class gene products, maybe via the ERK-A kinase. (PubMed:8381718).
Developmental stage: Expressed both maternally and zygotically. (PubMed:8381718).
Ptm: Phosphorylation on Ser/Thr by MAP kinase kinase kinases regulates positively the kinase activity.

(Information from UniProt)

mek-2 (WBGene00003186) associated phenotypes

AWA odorant chemotaxis defective, AWC odorant chemotaxis defective, bacterially unswollen, constipated, germ cell arrest, germ nuclei rachis, lethal, metabolic pathway variant, no oocytes, pachytene region organization variant, pathogen susceptibility increased, rod like larval lethal, sterile, vulval cell lineage variant, vulvaless

(Information from WormBase)

mek-2 (UniProt:Q10664) annotation

Function: Functions in the let-60 Ras signaling pathway; acts downstream of lin-45 raf kinase, but before the sur-1/mpK-1 gene product in controlling vulval cell differentiation.
Enzyme regulation: Activated by tyrosine and threoninephosphorylation catalyzed by MAP kinase kinase kinases.

(Information from UniProt)

Triosephosphate isomerase

Best gene implication in ageing for this target family came from gene Q10657 annotated in UniProt release 2014_02. Annotation GO 8340 (determination of adult lifespan) was Inferred from Mutant Phenotype

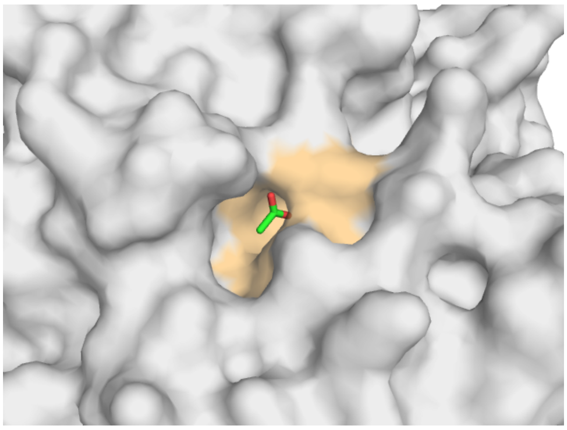

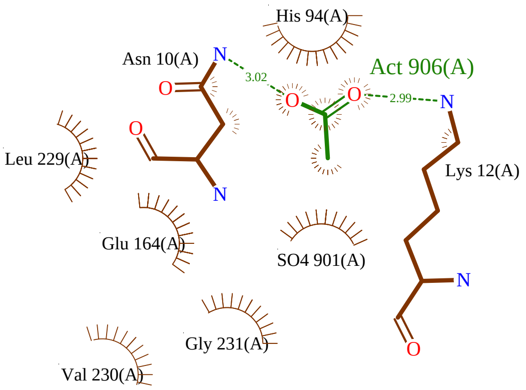

protein	amino acids contacts (binding site)
PDB:1mo0:chainA:Q10657	N K H E L V G
sp:P60174:TPIS_HUMAN	N K H E L V G
tr:M0R4U4:M0R4U4_RAT	N K H E L V G
sp:P48500:TPIS_RAT	N K H E L V G
sp:P17751:TPIS_MOUSE	N K H E L V G
sp:P29613:TPIS_DROME	N K H E L V G
sp:Q10657:TPIS_CAEEL	N K H E L V G
sp:P00942:TPIS_YEAST	N K H E L V G

	whole protein		domain-based		contact-based	
protein	ident	simil	ident	simil	ident	simil
PDB:1mo0:chainA:Q10657	0.96	0.96	1.0	1.0	1.0	1.0
sp:P60174:TPIS_HUMAN	0.54	0.75	0.62	0.87	1.0	1.0
tr:M0R4U4:M0R4U4_RAT	0.55	0.77	0.58	0.81	1.0	1.0
sp:P48500:TPIS_RAT	0.61	0.85	0.62	0.86	1.0	1.0
sp:P17751:TPIS_MOUSE	0.51	0.71	0.62	0.87	1.0	1.0
sp:P29613:TPIS_DROME	0.63	0.86	0.63	0.86	1.0	1.0
sp:Q10657:TPIS_CAEEL	1.0	1.0	1.0	1.0	1.0	1.0
sp:P00942:TPIS_YEAST	0.54	0.84	0.54	0.84	1.0	1.0

Tpi (FBgn0086355) associated phenotypes

bang sensitive, chemical conditional, chemical resistant, chemical sensitive, fast, heat sensitive, heat

stress response defective, neuroanatomy defective, oxidative stress response defective, short lived, stress response defective

(Information from FlyBase)

Tpi (UniProt:P29613) annotation
Pathway: Carbohydrate biosynthesis; gluconeogenesis.
Pathway: Carbohydrate degradation; glycolysis; D-glyceraldehyde 3- phosphate from glycerone phosphate: step 1/1.
Subunit: Homodimer.
Developmental stage: Present in substantial amounts in oocytes, declines in abundance in early embryos, and begins to increase during mid-embryogenesis. Levels peak in the third instar larva then decline during pupal stages, rising again near the time of eclosion. (PubMed:1720860).

(Information from UniProt)

tpi-1 (UniProt:Q10657) annotation
Pathway: Carbohydrate biosynthesis; gluconeogenesis.
Pathway: Carbohydrate degradation; glycolysis; D-glyceraldehyde 3- phosphate from glycerone phosphate: step 1/1.
Subunit: Homodimer.

(Information from UniProt)

Histone deacetylase 1

Best gene implication in ageing for this target family came from gene Q94517 annotated in UniProt release 2014_02. Annotation GO 8340 (determination of adult lifespan) was Inferred from Mutant Phenotype

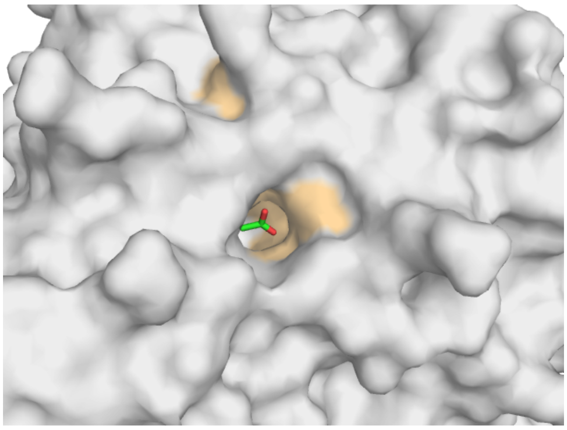

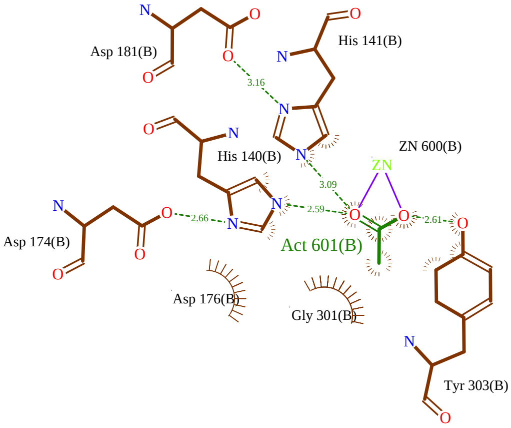

protein	amino acids contacts (binding site)
PDB:4bkx:chainB:Q13547	H H D H D G Y
sp:O15379:HDAC3_HUMAN	H H D H D G Y
tr:B3KRS5:B3KRS5_HUMAN	H H D H D G Y
sp:Q13547:HDAC1_HUMAN	H H D H D G Y
tr:Q6IT96:Q6IT96_HUMAN	H H D H D G Y
sp:Q92769:HDAC2_HUMAN	H H D H D G Y
tr:J3KPW7:J3KPW7_HUMAN	H H D H D G Y
sp:Q4QQW4:HDAC1_RAT	H H D H D G Y
tr:F7ENH8:F7ENH8_RAT	H H D H D G Y
tr:B1WBY8:B1WBY8_RAT	H H D H D G Y
sp:P70288:HDAC2_MOUSE	H H D H D G Y
tr:Q58E49:Q58E49_MOUSE	H H D H D G Y
sp:O09106:HDAC1_MOUSE	H H D H D G Y
tr:D3YYI8:D3YYI8_MOUSE	H H D H D G Y
sp:Q94517:HDAC1_DROME	H H D H D G Y
tr:I1V501:I1V501_DROME	H H D H D G Y
sp:O17695:HDA1_CAEEL	H H D H D G Y
tr:G5ECH0:G5ECH0_CAEEL	H H D H D G Y
sp:P32561:RPD3_YEAST	H H D H D G Y

	whole protein		domain-based		contact-based	
protein	ident	simil	ident	simil	ident	simil
PDB:4bkx:chainB:Q13547	1.0	1.0	1.0	1.0	1.0	1.0
sp:O15379:HDAC3_HUMAN	0.51	0.76	0.62	0.88	1.0	1.0
tr:B3KRS5:B3KRS5_HUMAN	0.81	0.89	0.88	0.92	1.0	1.0
sp:Q13547:HDAC1_HUMAN	1.0	1.0	1.0	1.0	1.0	1.0
tr:Q6IT96:Q6IT96_HUMAN	1.0	1.0	1.0	1.0	1.0	1.0
sp:Q92769:HDAC2_HUMAN	0.84	0.95	0.93	0.98	1.0	1.0
tr:J3KPW7:J3KPW7_HUMAN	0.71	0.79	0.93	0.98	1.0	1.0
sp:Q4QQW4:HDAC1_RAT	0.99	1.0	1.0	1.0	1.0	1.0
tr:F7ENH8:F7ENH8_RAT	0.84	0.94	0.93	0.98	1.0	1.0
tr:B1WBY8:B1WBY8_RAT	0.75	0.84	0.93	0.98	1.0	1.0
sp:P70288:HDAC2_MOUSE	0.84	0.94	0.93	0.98	1.0	1.0
tr:Q58E49:Q58E49_MOUSE	0.99	1.0	1.0	1.0	1.0	1.0
sp:O09106:HDAC1_MOUSE	0.99	1.0	1.0	1.0	1.0	1.0
tr:D3YYI8:D3YYI8_MOUSE	0.99	1.0	0.99	1.0	1.0	1.0
sp:Q94517:HDAC1_DROME	0.7	0.85	0.86	0.96	1.0	1.0
tr:I1V501:I1V501_DROME	0.69	0.84	0.85	0.96	1.0	1.0
sp:O17695:HDA1_CAEEL	0.59	0.83	0.7	0.92	1.0	1.0
tr:G5ECH0:G5ECH0_CAEEL	0.57	0.82	0.69	0.91	1.0	1.0
sp:P32561:RPD3_YEAST	0.51	0.74	0.64	0.9	1.0	1.0

Rpd3 (FBgn0015805) associated phenotypes

decreased cell number, dominant, drug conditional, enhancer of variegation, germline clone, increased cell death, lethal - all die before end of pupal stage, lethal - all die before end of second instar larval stage, long lived, maternal effect, mitotic cell cycle defective, neuroanatomy defective, pair rule phenotype, partially lethal - majority die, polyphasic, poor, somatic clone, some die during pupal stage, some die during second instar larval stage, suppressor of variegation

(Information from FlyBase)

Rpd3 (UniProt:Q94517) annotation

Function: Catalyzes the deacetylation of lysine residues on the N- terminal part of the core histones (H2A, H2B, H3 and H4). Histone deacetylation may constitute a tag for epigenetic repression and plays an important role in transcriptional regulation, cell cycle progression and developmental events. For instance, deacetylation of histone H3 may be a prerequisite for the subsequent recruitment of the histone methyltransferase Su(var)3-9 to histones. Involved in position-effect variegation (PEV).
Subunit: Interacts with Su(var)3-9. Component of a form of the Esc/E(z) complex present specifically during early embryogenesis which is composed of Caf1, esc, E(z), Su(z)12, Pcl and Rpd3.

The Esc/E(z) complex may also associate with Pcl and Rpd3 during early embryogenesis. This complex is distinct from the PRC1 complex, which contains many other PcG proteins like Pc, Ph, Psc, Su(z)2. The 2 complexes however cooperate and interact together during the first 3 hours of development to establish PcG silencing. Interacts with the histone methyltransferase Su(var)3-9. Component of a complex that contains at least Rpd3, CoRest and Su(var)3-3/Hdm. Component of the DREAM complex at least composed of Myb, Caf1, mip40, mip120, mip130, E2f2, Dp, Rbf, Rbf2, lin-52, Rpd3 and l(3)mbt. Interacts with neuronal repressor Ttk88. (PubMed:11571273, PubMed:12408863, PubMed:12533794, PubMed:12697833, PubMed:15306652, PubMed:15545624).
Subcellular location: Nucleus ECO:0000305.

(Information from UniProt)

Rpd3 (UniProt:I1V501) annotation
Subcellular location: Nucleus ECO:0000256—PIRNR:PIRNR037913.

(Information from UniProt)

hda-1 (WBGene00001834) associated phenotypes
giant oocytes, gonad morphology variant, somatic gonad morphology variant, sterile, tail morphology variant

(Information from WormBase)

hda-1 (UniProt:O17695) annotation
Function: Responsible for the deacetylation of lysine residues on the N-terminal part of the core histones (H2A, H2B, H3 and H4). Histone deacetylation gives a tag for epigenetic repression and plays an important role in transcriptional regulation, cell cycle progression and developmental events. Histone deacetylases act via the formation of large multiprotein complexes. Involved in the endoderm determination possibly by repressing end-1 expression. Also involved in vulval development, possibly by repressing lag-2 expression. (PubMed:11742996, PubMed:11940660).
Subunit: Interacts with pop-1. ECO:0000269—PubMed:11742996.
Subcellular location: Nucleus. Cytoplasm. Note=Except in the germ line where it is also cytoplasmic.
Tissue specificity: Ubiquitously expressed throughout embryonic and postembryonic development. (PubMed:11940660).
Developmental stage: Expressed both maternally and zygotically. (PubMed:11940660).
Ptm: Sumoylated. (PubMed:15990876).

(Information from UniProt)

hda-3 (WBGene00001836) associated phenotypes
egg laying variant, fertility reduced

(Information from WormBase)

hda-3 (UniProt:G5ECH0) annotation
Subcellular location: Nucleus ECO:0000256—PIRNR:PIRNR037913.

(Information from UniProt)

Kelch-like ECH-associated protein 1

Best gene implication in ageing for this target family came from gene Q8IGL4 via mapping the annotation from Ensembl FBgn0038475 via mapping the annotation from EntrezGene 42062 via mapping the annotation from GenAgeModels 0859 annotated in GenAge release 17.

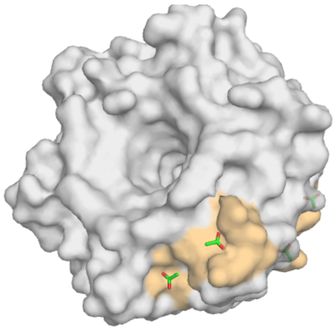

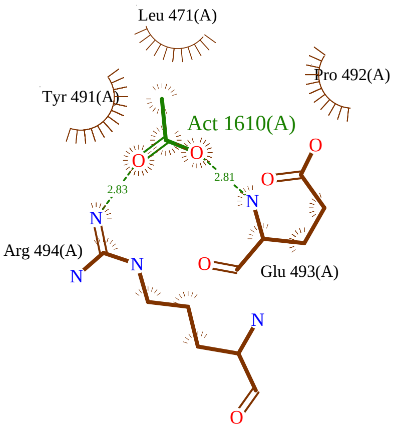

protein	amino acids contacts (binding site)
PDB:3zgc:chainA:Q14145	Y V G R P D G N P V R H C I H H R I L Y F G T R Y P E R E R I S Y Q Y S T
PDB:3zgd:chainA:Q14145	Y V G R P D G N P V R H C I H H R I L Y F G T R Y P E R E R I S Y Q Y S T
PDB:4l7b:chainA:Q14145	Y V G R P D G N P V R H C I H H R I L Y F G T R Y P E R E R I S Y Q Y S T
PDB:4l7c:chainA:Q14145	Y V G R P D G N P V R H C I H H R I L Y F G T R Y P E R E R I S Y Q Y S T
PDB:4l7d:chainA:Q14145	Y V G R P D G N P V R H C I H H R I L Y F G T R Y P E R E R I S Y Q Y S T
sp:Q14145:KEAP1_HUMAN	Y V G R P D G N P V R H C I H H R I L Y F G T R Y P E R E R I S Y Q Y S T
tr:G3V8U2:G3V8U2_RAT	Y V G R P D G N P V R H C I H H R I L Y F G T R Y P E R E R I S Y Q Y S T
tr:Q3UWI4:Q3UWI4_MOUSE	Y V G R P D G N P V R H C I H H R I L Y F G T R Y P E R E R I S Y Q Y S T
sp:Q9Z2X8:KEAP1_MOUSE	Y V G R P D G N P V R H C I H H R I L Y F G T R Y P E R E R I S Y Q Y S T
tr:Q8IGL4:Q8IGL4_DROME	F F G R I G S S A V R A M E Y H K L L Y F G N R H P E N E S L S F Q Y S I
tr:Q9VEN5:Q9VEN5_DROME	F F G R I G S S A V R A M E Y H K L L Y F G N R H P E N E S L S F Q Y S I
tr:A9UNI1:A9UNI1_DROME	F F G R I G S S A V R A M E Y H K L L Y F G N R H P E N E S L S F Q Y S I
tr:Q7KSF5:Q7KSF5_DROME	F F G R I G S S A V R A M E Y H K L L Y F G N R H P E N E S L S F Q Y S I

	whole protein		domain-based		contact-based	
protein	ident	simil	ident	simil	ident	simil
PDB:3zgc:chainA:Q14145	0.99	1.0	0.99	1.0	1.0	1.0
PDB:3zgd:chainA:Q14145	0.99	1.0	0.99	1.0	1.0	1.0
PDB:4l7b:chainA:Q14145	0.99	1.0	0.99	1.0	1.0	1.0
PDB:4l7c:chainA:Q14145	0.99	1.0	0.99	1.0	1.0	1.0
PDB:4l7d:chainA:Q14145	0.99	1.0	0.99	1.0	1.0	1.0
sp:Q14145:KEAP1_HUMAN	1.0	1.0	1.0	1.0	1.0	1.0
tr:G3V8U2:G3V8U2_RAT	0.94	0.98	0.97	0.99	1.0	1.0
tr:Q3UWI4:Q3UWI4_MOUSE	0.6	0.62	0.97	0.99	1.0	1.0
sp:Q9Z2X8:KEAP1_MOUSE	0.94	0.98	0.97	0.99	1.0	1.0
tr:Q8IGL4:Q8IGL4_DROME	0.31	0.5	0.46	0.74	0.46	0.65
tr:Q9VEN5:Q9VEN5_DROME	0.37	0.64	0.46	0.74	0.46	0.65
tr:A9UNI1:A9UNI1_DROME	0.36	0.63	0.46	0.74	0.46	0.65
tr:Q7KSF5:Q7KSF5_DROME	0.36	0.63	0.46	0.74	0.46	0.65

Keap1 (FBgn0038475) associated phenotypes

developmental rate defective, dominant, large body, long lived, male limited, oxidative stress response defective

(Information from FlyBase)

Werner syndrome ATP-dependent helicase

Best gene implication in ageing for this target family came from gene Q19546 annotated in UniProt release 2014_02. Annotation GO 8340 (determination of adult lifespan) was Inferred from Mutant Phenotype

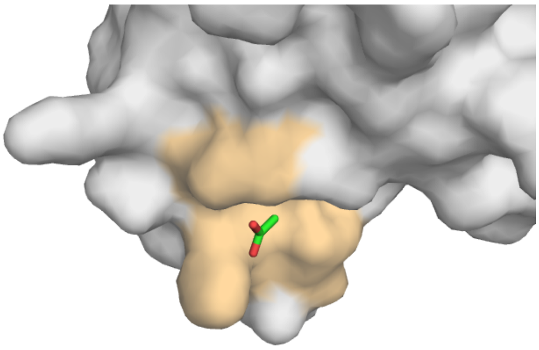

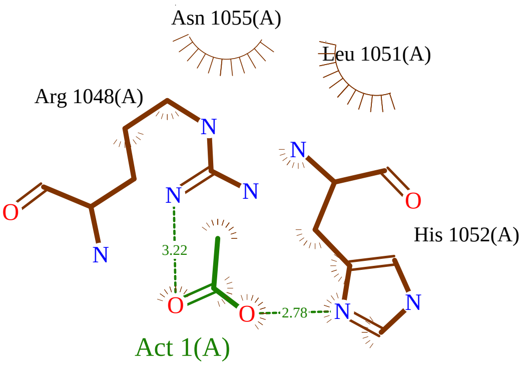

protein	amino acids contacts (binding site)
PDB:3aaf:chainA:Q14191	R L H N
sp:Q14191:WRN_HUMAN	R L H N
tr:F1LTH9:F1LTH9_RAT	R L G I
tr:Q8BWH5:Q8BWH5_MOUSE	R L G S
sp:O09053:WRN_MOUSE	R L G S
sp:Q19546:WRN_CAEEL	E L K K
sp:P35187:SGS1_YEAST	P - - -

	whole protein		domain-based		contact-based	
protein	ident	simil	ident	simil	ident	simil
PDB:3aaf:chainA:Q14191	1.0	1.0	1.0	1.0	1.0	1.0
sp:Q14191:WRN_HUMAN	1.0	1.0	1.0	1.0	1.0	1.0
tr:F1LTH9:F1LTH9_RAT	0.69	0.88	0.76	0.93	0.5	0.43
tr:Q8BWH5:Q8BWH5_MOUSE	0.2	0.26	0.2	0.28	0.5	0.67
sp:O09053:WRN_MOUSE	0.7	0.88	0.75	0.93	0.5	0.67
sp:Q19546:WRN_CAEEL	0.19	0.49	0.28	0.68	0.25	0.66
sp:P35187:SGS1_YEAST	0.16	0.5	0.2	0.62	0.0	0.0

wrn-1 (WBGene00006944) associated phenotypes

organism hypersensitive ionizing radiation

(Information from WormBase)

wrn-1 (UniProt:Q19546) annotation

Function: Essential for the formation of DNA replication focal centers; stably associates with foci elements generating binding sites for RP-A. Exhibits a magnesium-dependent ATP-dependent DNA- helicase activity. May be involved in the control of genomic stability (By similarity).
Subcellular location: Nucleus

(Information from UniProt)

Mitogen-activated protein kinase 14

Best gene implication in ageing for this target family came from gene O61443 annotated in UniProt release 2014_02. Annotation GO 8340 (determination of adult lifespan) was Inferred from Mutant Phenotype

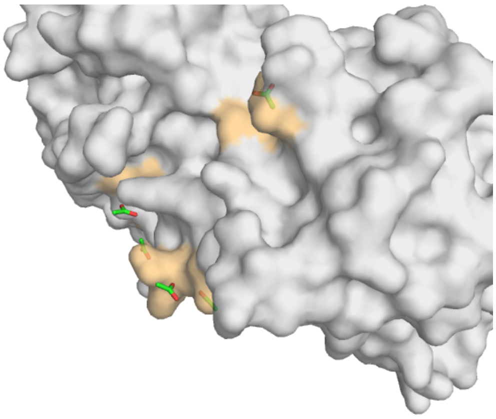

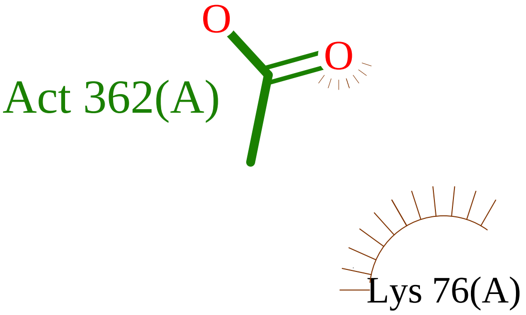

protein	amino acids contacts (binding site)
PDB:2zaz:chainA:Q16539	P P H Y K D V M A S A D A V N E L K L V A D P Y S F R
PDB:3kq7:chainA:Q16539	P P H Y K D V M A S A D A V N E L K L V A D P Y S F R
PDB:4ewq:chainA:Q16539	P P H Y K D V M A S A D A V N E L K L V A D P Y S F R
PDB:4f9w:chainA:Q16539	P P H Y K D V M A S A D A V N E L K L V A D P Y S F R
tr:L7RSM2:L7RSM2_HUMAN	P P H Y K D V M A S A D A V N E L K L V A D P Y S F R
sp:Q16539:MK14_HUMAN	P P H Y K D V M A S A D A V N E L K L V A D P Y S F R
tr:G3V617:G3V617_RAT	P P H Y K D V M A S A D A V N E L K L V A D P Y S F R
tr:Q56A33:Q56A33_RAT	P P H Y K D V M A S A D A V N E L K L V A D P Y S F R
sp:P47811:MK14_MOUSE	P P H Y K D V M A S A D A V N E L K L V A D P Y S F R
tr:Q5U421:Q5U421_MOUSE	P P H Y K D V M A S A D A V N E L K L V A D P Y S F R
sp:O61443:MK14B_DROME	A P H Y K D V M A S A G A V N E L R L T A A L Y S F N
sp:Q17446:PMK1_CAEEL	E P H Y R D V M A S A D A V N E L K L I A E E M N D R
sp:P32485:HOG1_YEAST	E T L Y K D I Q T S A G L I N D L K C V A D A F H F A

protein	whole protein ident	whole protein simil	domain-based ident	domain-based simil	contact-based ident	contact-based simil
PDB:2zaz:chainA:Q16539	1.0	1.0	1.0	1.0	1.0	1.0
PDB:3kq7:chainA:Q16539	1.0	1.0	1.0	1.0	1.0	1.0
PDB:4ewq:chainA:Q16539	1.0	1.0	1.0	1.0	1.0	1.0
PDB:4f9w:chainA:Q16539	1.0	1.0	1.0	1.0	1.0	1.0
tr:L7RSM2:L7RSM2_HUMAN	0.96	0.99	0.96	0.99	1.0	1.0
sp:Q16539:MK14_HUMAN	1.0	1.0	1.0	1.0	1.0	1.0
tr:G3V617:G3V617_RAT	0.99	1.0	0.99	1.0	1.0	1.0
tr:Q56A33:Q56A33_RAT	0.95	0.99	0.95	0.99	1.0	1.0
sp:P47811:MK14_MOUSE	0.99	1.0	0.99	1.0	1.0	1.0
tr:Q5U421:Q5U421_MOUSE	0.96	0.99	0.95	0.99	1.0	1.0
sp:O61443:MK14B_DROME	0.68	0.89	0.69	0.89	0.74	0.81
sp:Q17446:PMK1_CAEEL	0.62	0.85	0.62	0.85	0.7	0.81
sp:P32485:HOG1_YEAST	0.41	0.68	0.49	0.82	0.44	0.61

p38b (FBgn0024846) associated phenotypes

RU486 conditional, circadian rhythm defective, decreased cell size, developmental rate defective, drug conditional, heat stress response defective, immune response defective, locomotor behavior defective, long lived, melanotic mass phenotype, neuroanatomy defective, nutrition conditional, osmotic stress response defective, oxidative stress response defective, partially lethal - majority die, progressive, short lived, small body, starvation stress response defective

(Information from FlyBase)

p38b (UniProt:O61443) annotation

Function: Kinase involved in dpp signal transduction pathway in the process of wing morphogenesis when the levels of dpp are enhanced or inhibited. May down-regulate insect immunity gene expression after prolonged infection. (PubMed:10022918, PubMed:9584193).
Cofactor:Mg(2+)
Enzyme regulation: Activated by threonine and tyrosine phosphorylation by Mkk3. (PubMed:9584193).

Subcellular location: Nucleus.
Tissue specificity: At mid-embryogenesis, highest expression is seen in developing anterior and posterior midguts. Almost ubiquitous expression throughout all development.
Developmental stage: Expressed both maternally and zygotically in the embryo, expression seen in all developmental stages.
Domain: The TXY motif contains the threonine and tyrosine residues whose phosphorylation activates the MAP kinases.
Ptm: Dually phosphorylated on Thr-183 and Tyr-185, which activates the enzyme.

(Information from UniProt)

pmk-1 (WBGene00004055) associated phenotypes

cell stress response variant, drug induced gene expression variant, gene expression level reduced, lethal, life span variant, organism oxidative stress response hypersensitive, pathogen induced gene expression variant, pathogen susceptibility increased, sterile

(Information from WormBase)

pmk-1 (UniProt:Q17446) annotation

Function: Responds to activation by environmental stress and pro- inflammatory cytokines by phosphorylating downstream targets. Functions downstream of the MAPKK sek-1 and the MAPKKK nsy-1 as the MAP kinase required for pathogen resistance. (PubMed:11703092, PubMed:12142542).
Cofactor: Mg(2+) Evidence=(PubMed:11703092);
Enzyme regulation: Activated by phosphorylation on threonine and tyrosine. Inhibited by pyridinyl-imidazole related compounds. (PubMed:11703092).
Domain: The TXY motif contains the threonine and tyrosine residues whose phosphorylation activates the MAP kinases.
Ptm: Dually phosphorylated on Thr-191 and Tyr-193, which activates the enzyme.

(Information from UniProt)

NAD-dependent protein deacetylase sirtuin-2

Best gene implication in ageing for this target family came from gene Q9I7I7 annotated in UniProt release 2014_02. Annotation GO 8340 (determination of adult lifespan) was Inferred from Mutant Phenotype

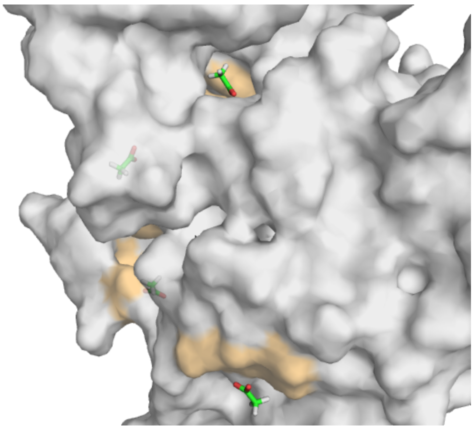

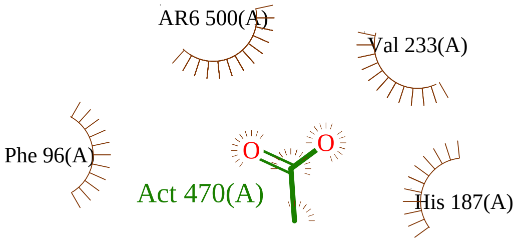

protein	amino acids contacts (binding site)
PDB:3zgv:chainA:Q8IXJ6	F R E H V Q S M G G G W L
sp:Q8IXJ6:SIR2_HUMAN	F R E H V Q S M G G G W L
sp:Q5RJQ4:SIR2_RAT	F R E H V Q T M G G G W L
tr:Q3UJK6:Q3UJK6_MOUSE	F R E H V Q T M G G G W L
sp:Q8VDQ8:SIR2_MOUSE	F R E H V Q T M G G G W L
sp:Q9I7I7:SIRT2_DROME	F R T H V E A F T R S F L
sp:P53686:HST2_YEAST	F R E H V A - - - - - V H

	whole protein		domain-based		contact-based	
protein	ident	simil	ident	simil	ident	simil
PDB:3zgv:chainA:Q8IXJ6	1.0	1.0	1.0	1.0	1.0	1.0
sp:Q8IXJ6:SIR2_HUMAN	1.0	1.0	1.0	1.0	1.0	1.0
sp:Q5RJQ4:SIR2_RAT	0.78	0.87	0.9	0.97	0.92	0.95
tr:Q3UJK6:Q3UJK6_MOUSE	0.79	0.87	0.9	0.97	0.92	0.95
sp:Q8VDQ8:SIR2_MOUSE	0.88	0.97	0.9	0.98	0.92	0.95
sp:Q9I7I7:SIRT2_DROME	0.43	0.72	0.52	0.81	0.38	0.66
sp:P53686:HST2_YEAST	0.29	0.57	0.39	0.7	0.38	0.09

Sirt2 (FBgn0038788) associated phenotypes
heat sensitive, lethal - all die before end of pupal stage, some die during pupal stage

(Information from FlyBase)

Sirt2 (UniProt:Q9I7I7) annotation
Function: NAD-dependent protein deacetylase (By similarity). May be involved in the regulation of life span. (, PubMed:17159295).
Cofactor: Zn(2+)Note=Binds 1 zinc ion per subunit. ;
Disruption phenotype: Causes lethality during development. Induced silencing shortens life span. (PubMed:17159295).

(Information from UniProt)

Histone acetyltransferase KAT6A

Best gene implication in ageing for this target family came from gene Q92794 annotated in UniProt release 2014_02. Annotation GO subterm of 7568 (aging) was Inferred from Mutant Phenotype

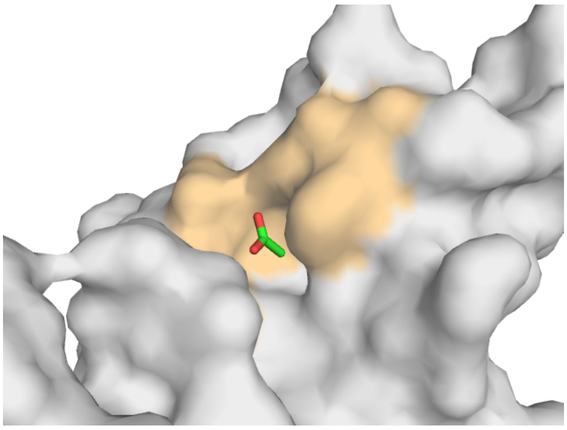

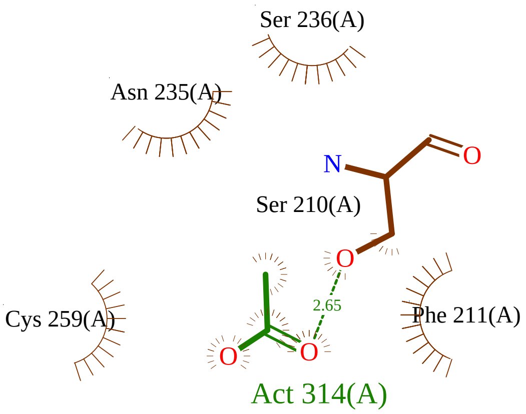

protein	amino acids contacts (binding site)
PDB:3v43:chainA:Q92794	S F N S C
tr:A5PLL3:A5PLL3_HUMAN	S F N S C
tr:A5PKX7:A5PKX7_HUMAN	S F N S C
sp:Q92794:KAT6A_HUMAN	S F N S C
tr:Q3V1G6:Q3V1G6_MOUSE	S F N S C
tr:Q3UPM9:Q3UPM9_MOUSE	S F N S C
tr:G3X940:G3X940_MOUSE	S F N S C
tr:D0IQI5:D0IQI5_DROME	T H I S C
tr:Q9W1A9:Q9W1A9_DROME	T H I S C
tr:G5EBH5:G5EBH5_CAEEL	E N D S G
tr:K8ERR8:K8ERR8_CAEEL	E N D S G
sp:Q08649:ESA1_YEAST	- - H D -

	whole protein		domain-based		contact-based	
protein	ident	simil	ident	simil	ident	simil
PDB:3v43:chainA:Q92794	0.98	1.0	1.0	1.0	1.0	1.0
tr:A5PLL3:A5PLL3_HUMAN	0.41	0.41	1.0	1.0	1.0	1.0
tr:A5PKX7:A5PKX7_HUMAN	0.57	0.57	1.0	1.0	1.0	1.0
sp:Q92794:KAT6A_HUMAN	1.0	1.0	1.0	1.0	1.0	1.0
tr:Q3V1G6:Q3V1G6_MOUSE	0.52	0.55	0.97	0.99	1.0	1.0
tr:Q3UPM9:Q3UPM9_MOUSE	0.57	0.62	0.97	0.99	1.0	1.0
tr:G3X940:G3X940_MOUSE	0.89	0.96	0.97	0.99	1.0	1.0
tr:D0IQI5:D0IQI5_DROME	0.13	0.26	0.23	0.56	0.4	0.42
tr:Q9W1A9:Q9W1A9_DROME	0.16	0.54	0.23	0.56	0.4	0.42
tr:G5EBH5:G5EBH5_CAEEL	0.12	0.45	0.11	0.54	0.2	0.26
tr:K8ERR8:K8ERR8_CAEEL	0.12	0.45	0.11	0.54	0.2	0.26
sp:Q08649:ESA1_YEAST	0.07	0.16	0.03	0.08	0.0	0.0

enok-RA (UniProt:D0IQI5) annotation
Subcellular location: Nucleus ECO:0000256—RuleBase:RU361211.

(Information from UniProt)

enok (FBgn0034975) associated phenotypes
female semi-sterile, germline clone, neuroanatomy defective, size defective, somatic clone

(Information from FlyBase)

enok (UniProt:Q9W1A9) annotation
Subcellular location: Nucleus ECO:0000256—RuleBase:RU361211.

(Information from UniProt)

lsy-12 (WBGene00045419) associated phenotypes
egg laying defective, locomotion variant, loss of asymmetry ASE, protruding vulva, reduced brood size, sick

(Information from WormBase)

lsy-12 (UniProt:G5EBH5) annotation
Subcellular location: Nucleus ECO:0000256—RuleBase:RU361211.

(Information from UniProt)

lsy-12 (UniProt:K8ERR8) annotation
Subcellular location: Nucleus ECO:0000256—RuleBase:RU361211.

(Information from UniProt)

Mitogen-activated protein kinase kinase kinase 5

Best gene implication in ageing for this target family came from gene P23561 via mapping the annotation from Ensembl YLR362W via mapping the annotation from EntrezGene 851076 via mapping

the annotation from GenAgeModels 1609 annotated in GenAge release 17.

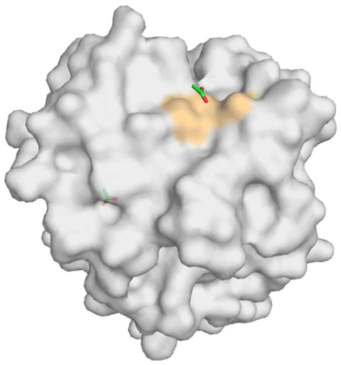

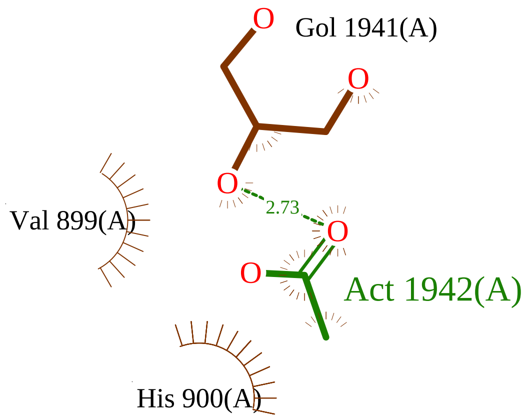

protein	amino acids contacts (binding site)				
PDB:4bib:chainA:Q99683	E	R	Q	V	H
PDB:4bid:chainA:Q99683	E	R	Q	V	H
sp:Q99683:M3K5_HUMAN	E	R	Q	V	H
tr:D3ZW27:D3ZW27_RAT	E	R	Q	V	H
tr:D3ZZH6:D3ZZH6_RAT	E	R	Q	V	H
tr:E9PWG9:E9PWG9_MOUSE	E	R	Q	V	H
sp:O35099:M3K5_MOUSE	E	R	Q	V	H
tr:Q24458:Q24458_DROME	D	R	Q	K	H
tr:Q7KSB7:Q7KSB7_DROME	D	R	Q	K	H
tr:Q8I6M5:Q8I6M5_DROME	D	R	Q	K	H
tr:Q9VDS9:Q9VDS9_DROME	D	R	Q	K	H
tr:Q4QQD0:Q4QQD0_DROME	D	R	Q	K	H
tr:B9EQQ2:B9EQQ2_DROME	D	R	Q	K	H
tr:Q21029:Q21029_CAEEL	E	R	Q	T	H
sp:P23561:STE11_YEAST	D	M	G	T	T

	whole protein		domain-based		contact-based	
protein	ident	simil	ident	simil	ident	simil
PDB:4bib:chainA:Q99683	1.0	1.0	1.0	1.0	1.0	1.0
PDB:4bid:chainA:Q99683	1.0	1.0	1.0	1.0	1.0	1.0
sp:Q99683:M3K5_HUMAN	1.0	1.0	1.0	1.0	1.0	1.0
tr:D3ZW27:D3ZW27_RAT	0.91	0.96	0.97	0.99	1.0	1.0
tr:D3ZZH6:D3ZZH6_RAT	0.92	0.97	0.97	0.99	1.0	1.0
tr:E9PWG9:E9PWG9_MOUSE	0.94	0.97	0.98	0.99	1.0	1.0
sp:O35099:M3K5_MOUSE	0.94	0.98	0.98	0.99	1.0	1.0
tr:Q24458:Q24458_DROME	0.22	0.38	0.65	0.88	0.6	0.67
tr:Q7KSB7:Q7KSB7_DROME	0.33	0.59	0.65	0.88	0.6	0.67
tr:Q8I6M5:Q8I6M5_DROME	0.36	0.68	0.65	0.88	0.6	0.67
tr:Q9VDS9:Q9VDS9_DROME	0.36	0.68	0.65	0.88	0.6	0.67
tr:Q4QQD0:Q4QQD0_DROME	0.36	0.68	0.65	0.88	0.6	0.67
tr:B9EQQ2:B9EQQ2_DROME	0.36	0.68	0.65	0.88	0.6	0.67
tr:Q21029:Q21029_CAEEL	0.31	0.65	0.56	0.82	0.8	0.84
sp:P23561:STE11_YEAST	0.1	0.28	0.28	0.59	0.0	0.59

Pk92B (FBgn0014006) associated phenotypes

heat sensitive

(Information from FlyBase)

nsy-1 (WBGene00003822) associated phenotypes

aldicarb hypersensitive, anoxia resistant, benzaldehyde chemotaxis variant, butanone chemotaxis variant, cell stress response variant, egg laying defective, egg laying response to food variant, gene expression level reduced, genetic pathway activation variant, lethal, levamisole hypersensitive, loss

of asymmetry AWC, odorant adaptation variant, organism high sodium chloride hypersensitive, organism stress response variant, pathogen induced gene expression variant, pathogen susceptibility increased, pentanedione chemotaxis variant, protein phosphorylation reduced, pyrazine chemotaxis variant, sterile, transgene expression reduced

(Information from WormBase)

Potassium/sodium hyperpolarization-activated cyclic nucleotide-gated channel 4

Best gene implication in ageing for this target family came from gene Q8IGX2 annotated in UniProt release 2014_02. Annotation GO 8340 (determination of adult lifespan) was Inferred from Mutant Phenotype

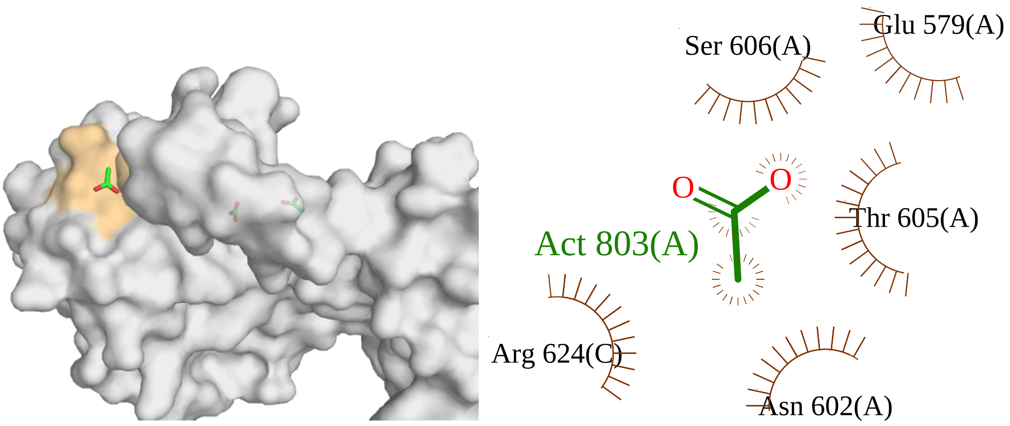

protein	amino acids contacts (binding site)
PDB:4kl1:chainA:Q9Y3Q4	E N T S H G V K L A D Y
sp:Q9Y3Q4:HCN4_HUMAN	E N T S H G V K L A D Y
sp:Q9JKA7:HCN4_RAT	E N T S H G V K L A D Y
tr:B2RY58:B2RY58_MOUSE	E N T S H G V K L A D Y
tr:Q8IGX2:Q8IGX2_DROME	E N S D E G V S L S D Y
tr:A1Z9P0:A1Z9P0_DROME	E N S D E G V S L S D Y
tr:Q56JH9:Q56JH9_DROME	E N S D E G V S L S D Y
tr:Q56JH8:Q56JH8_DROME	E N S D E G V S L S D Y
tr:F7VJU3:F7VJU3_DROME	E N S D E G V S L S D Y
tr:Q9Y1J9:Q9Y1J9_DROME	E N S D E G V S L S D Y
tr:Q56JI0:Q56JI0_DROME	E N S D E G V S L S D Y
tr:B7YZE7:B7YZE7_DROME	E N S D E G V S L S D Y
tr:A1Z9N8:A1Z9N8_DROME	E N S D E G V S L S D Y
tr:Q56JH7:Q56JH7_DROME	E N S D E G V S L S D Y
tr:B7YZE8:B7YZE8_DROME	E N S D E G V S L S D Y
tr:A1Z9N7:A1Z9N7_DROME	E N S D E G V S L S D Y

	whole protein		domain-based		contact-based	
protein	ident	simil	ident	simil	ident	simil
PDB:4kl1:chainA:Q9Y3Q4	0.99	1.0	0.99	0.99	1.0	1.0
sp:Q9Y3Q4:HCN4_HUMAN	1.0	1.0	1.0	1.0	1.0	1.0
sp:Q9JKA7:HCN4_RAT	0.96	0.98	1.0	1.0	1.0	1.0
tr:B2RY58:B2RY58_MOUSE	0.95	0.98	1.0	1.0	1.0	1.0
tr:Q8IGX2:Q8IGX2_DROME	0.19	0.28	0.68	0.91	0.58	0.7
tr:A1Z9P0:A1Z9P0_DROME	0.28	0.42	0.68	0.91	0.58	0.7
tr:Q56JH9:Q56JH9_DROME	0.27	0.41	0.68	0.91	0.58	0.7
tr:Q56JH8:Q56JH8_DROME	0.27	0.41	0.68	0.91	0.58	0.7
tr:F7VJU3:F7VJU3_DROME	0.27	0.41	0.68	0.91	0.58	0.7
tr:Q9Y1J9:Q9Y1J9_DROME	0.26	0.47	0.68	0.91	0.58	0.7
tr:Q56JI0:Q56JI0_DROME	0.21	0.38	0.68	0.91	0.58	0.7
tr:B7YZE7:B7YZE7_DROME	0.21	0.38	0.68	0.91	0.58	0.7
tr:A1Z9N8:A1Z9N8_DROME	0.21	0.38	0.68	0.91	0.58	0.7
tr:Q56JH7:Q56JH7_DROME	0.21	0.37	0.68	0.91	0.58	0.7
tr:B7YZE8:B7YZE8_DROME	0.21	0.37	0.68	0.91	0.58	0.7
tr:A1Z9N7:A1Z9N7_DROME	0.21	0.37	0.68	0.91	0.58	0.7

Ih (FBgn0263397) associated phenotypes

conditional, hypoactive, neurophysiology defective, optomotor response defective, proboscis extension reflex defective, short lived, sleep defective, uncoordinated

(Information from FlyBase)

Kelch-like ECH-associated protein 1

Best gene implication in ageing for this target family came from gene Q95SQ1 via mapping the annotation from Ensembl FBgn0038475 via mapping the annotation from EntrezGene 42062 via mapping the annotation from GenAgeModels 0859 annotated in GenAge release 17.

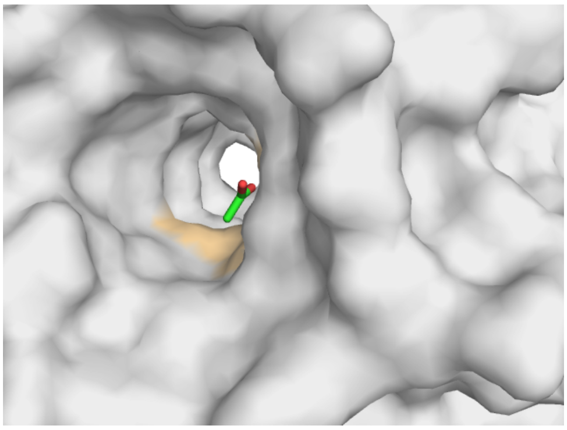

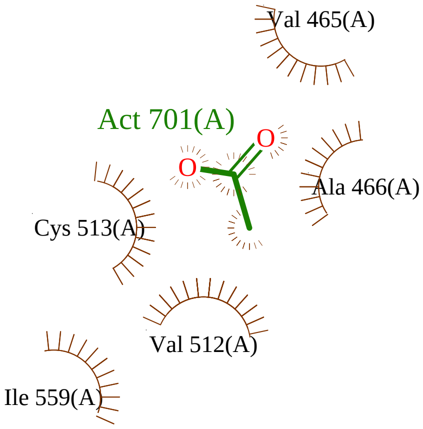

protein	amino acids contacts (binding site)				
PDB:3wn7:chainA:Q9Z2X8	V	A	V	C	I
sp:Q14145:KEAP1_HUMAN	V	A	V	C	I
tr:G3V8U2:G3V8U2_RAT	V	A	V	C	I
tr:Q3UWI4:Q3UWI4_MOUSE	V	A	V	C	I
sp:Q9Z2X8:KEAP1_MOUSE	V	A	V	C	I
tr:Q95SQ1:Q95SQ1_DROME	V	V	V	A	L
tr:Q8IGL4:Q8IGL4_DROME	V	V	V	A	L
tr:Q9VEN5:Q9VEN5_DROME	V	V	V	A	L
tr:A9UNI1:A9UNI1_DROME	V	V	V	A	L
tr:Q7KSF5:Q7KSF5_DROME	V	V	V	A	L

protein	whole protein ident	simil	domain-based ident	simil	contact-based ident	simil
PDB:3wn7:chainA:Q9Z2X8	1.0	1.0	1.0	1.0	1.0	1.0
sp:Q14145:KEAP1_HUMAN	0.94	0.98	0.97	0.99	1.0	1.0
tr:G3V8U2:G3V8U2_RAT	0.98	1.0	0.97	0.99	1.0	1.0
tr:Q3UWI4:Q3UWI4_MOUSE	0.63	0.63	1.0	1.0	1.0	1.0
sp:Q9Z2X8:KEAP1_MOUSE	1.0	1.0	1.0	1.0	1.0	1.0
tr:Q95SQ1:Q95SQ1_DROME	0.18	0.3	0.48	0.77	0.4	0.4
tr:Q8IGL4:Q8IGL4_DROME	0.3	0.5	0.53	0.85	0.4	0.4
tr:Q9VEN5:Q9VEN5_DROME	0.37	0.64	0.53	0.85	0.4	0.4
tr:A9UNI1:A9UNI1_DROME	0.36	0.63	0.53	0.85	0.4	0.4
tr:Q7KSF5:Q7KSF5_DROME	0.36	0.64	0.53	0.85	0.4	0.4

Keap1 (FBgn0038475) associated phenotypes

developmental rate defective, dominant, large body, long lived, male limited, oxidative stress response defective

(Information from FlyBase)
